# Identification of Functional Modules and Key Pathways Associated with Innervation in Graft Bone—CGRP Regulates the Differentiation of Bone Marrow Mesenchymal Stem Cells via p38 MAPK and Wnt6/*β*-Catenin

**DOI:** 10.1155/2023/1154808

**Published:** 2023-08-16

**Authors:** Ziqian Wu, Xudong Wang, Jingcun Shi, Anand Gupta, Yuhan Zhang, Bingqing Zhang, Yang Cao, Lei Wang

**Affiliations:** ^1^Department of Oral and Maxillofacial Surgery—Head & Neck Oncology, Shanghai Ninth People's Hospital, Shanghai Jiao Tong University School of Medicine, Shanghai 200011, China; ^2^Department of Stomatology, Oriental Hospital, Tongji University, 200120, Shanghai, China; ^3^Department of Dentistry, Government Medical College & Hospital, 160030, Chandigarh, India; ^4^Clinical Epidemiology and Biostatistics, School of Medical Sciences, Faculty of Medicine and Health, Örebro University, 70182, Örebro, Sweden; ^5^Unit of Integrative Epidemiology, Institute of Environmental Medicine, Karolinska Institutet, 17177, Stockholm, Sweden; ^6^Department of Stomatology, Fengcheng Hospital, Fengxian District, Shanghai 201411, China

## Abstract

Bone resorption occurs after bone grafting, however, contemporaneous reconstruction of the innervation of the bone graft is a potential treatment to maintain the bone mass of the graft. The innervation of bone is an emerging research topic. To understand the potential molecular mechanisms of bone innervation after bone grafting, we collected normal iliac bone tissue as well as bone grafts with or without innervation from nine patients 1 year after surgery and performed RNA sequencing. We identified differentially expressed genes) from these samples and used the gene ontology and Kyoto Encyclopedia of Genes and Genomes databases for functional enrichment and signaling pathway analysis. In parallel, we established protein–protein interaction networks to screen functional modules. Based on bioinformatic results, we validated *in vitro* the osteogenic differentiation potential of rat bone marrow mesenchymal stem cells (BMMSCs) after calcitonin gene-related peptide (CGRP) stimulation and the expression of p38 MAPK and Wnt6/*β*-catenin pathways during osteogenesis. Our transcriptome analysis of bone grafts reveals functional modules and signaling pathways of innervation which play a vital role in the structural and functional integration of the bone graft. Simultaneously, we demonstrate that CGRP regulates the differentiation of BMMSCs through p38 MAPK and Wnt6/*β*-catenin.

## 1. Introduction

Autograft bone reconstruction is considered the mainstay of treatment for large maxillofacial bone defects caused by tumors, trauma, congenital malformations, and other diseases. However, nonvascularized bone grafts are exposed to severe bone resorption and even postoperative osteoporosis inevitably occurs after vascularized bone grafting using microsurgical techniques [1, 2]. Currently, autografts (e.g., iliac crest autografts) remain the gold standard for the treatment of large bone defects due to their good shape, vascularization, and mechanical strength [3]. A variety of factors may affect the postoperative resorption of bone grafts, with crosstalk between bone and nerves being one of the current hot research topics. The peripheral nervous system (PNS), an important component of the skeletal microenvironment, is involved in bone metabolism and repair through a large number of signals generated by the secretion of neurotransmitters such as substance P (SP), nerve growth factor (NGF), calcitonin gene-related peptide (CGRP), and SSEMA3A [4, 5]. In the skeletal system, bone formation and maintenance processes are intricately regulated by osteoblasts, responsible for bone formation, and osteoclasts, responsible for bone resorption. Imbalances between these two cell types play a pivotal role in the pathogenesis and etiology of certain bone metabolic disorders, such as osteoporosis [6, 7]. Our previous studies confirmed that the nervous system regulates bone homeostasis mainly through BMMSCs, where the sympathetic nervous system (SNS) can inhibit bone remodeling by suppressing BMMSCs, and NGF and SP can promote bone formation through sensory nerves [8–11]. Recognizing the significant role of the nervous system in preserving skeletal homeostasis, our research team implemented neural reconstruction, coupled with the vascularization of bone grafts, as a strategy to mitigate postoperative osteoporosis. Remarkably, our observations revealed that bone grafts repaired through this novel approach successfully preserved the original bone mass and structural integrity following the surgical procedure [12].

In recent years, RNA sequencing (RNA-seq) has emerged as a powerful tool for comprehensive analysis of the global transcriptome, providing insights into physiological states and various bone disorders including osteoporosis, osteoarthritis, and bone-related tumors. At present, zebrafish and mice serve as predominant models for investigating the intricate interplay between bone and nerves. However, more sophisticated and refined models are warranted to gain deeper insights into the intricate molecular interactions underlying bone-nerve crosstalk [13]. Building upon previous research findings, our research team implemented a novel approach involving simultaneous reconstruction of the vascular and neural systems in the iliac flap to reconstruct the mandible. This innovative model provides an ideal platform for studying nerve-bone interactions based on the human body itself. In the present study, we obtained three sets of bone tissue samples from patients enrolled in our clinical trial INVITATION (Clinicaltrials.gov ID: NCT03889587) for subsequent RNA-seq analysis. The primary objective was to uncover functional modules and signaling pathways associated with the pathophysiological mechanisms underlying the effects of innervation on bone. Furthermore, we aimed to validate the underlying molecular mechanisms *in vitro*.

## 2. Materials and Methods

### 2.1. Patient Sample Collection Procedures

A total of nine patients participating in the INVITATION clinical trial (Clinicaltrials.gov ID: NCT03889587) were included in this study after providing informed consent (Table 1). The study employed specific inclusion and exclusion criteria. The inclusion criteria were as follows: (1) age between 18 and 60 years, irrespective of gender; (2) presence of jaw defects resulting from tumor, trauma, or congenital malformation; (3) segmental mandibular defects measuring 5–9 cm without preoperative lower lip numbness; (4) absence of menopausal osteoporosis and uncontrolled diabetes mellitus with fasting blood glucose levels ranging from 3.9 to 6.1 mmol/l; (5) no use of medications affecting bone metabolisms, such as bisphosphonates or calcium supplements; (6) provision of signed informed consent. The exclusion criteria consisted of (1) advanced cancer; (2) patients with poor physical condition who were unable to undergo vascularized bone flap repair.

During the process of mandibular reconstruction, normal iliac bone fragments produced during ilioplasty were collected as the presurgical control bone group (CO). Additionally, a nerve suture was performed between the ilioinguinal nerve innervating the iliac bone and the inferior alveolar nerve or the greater auricular nerve to restore innervation to the graft, creating the innervated bone graft group (IN). The conventional iliac bone without a nerve suture served as the noninnervated bone graft group (NI). Samples were obtained from both groups by extracting bone tissue at the site of the grafted bone using a loop drill during dental implantation, 1 year after mandibular reconstruction. All were collected fresh bone tissue, immediately cut into pieces smaller than 0.5 cm, immersed in a solution of RNA later five times the volume of the tissue, and frozen in liquid nitrogen. The samples were stored at −80°C and transported using dry ice.

### 2.2. Animal Model

The 4 week-old SD rats and 6 week nude mice used in the experiments were provided by the animal experiment center of the Ninth People's Hospital, Shanghai. The study was approved by the Ethics Committee of the Ninth People's Hospital, Shanghai Jiao Tong University School of Medicine (SH9H-2018-T95-2).

### 2.3. RNA Sequencing

From the patient's bone tissues, total RNA was extracted. A NanoDropTM ND-1000 was used to evaluate the amount and quality of the extracted RNA. RNA integrity was evaluated using denaturing agarose gel electrophoresis. The KAPA Stranded RNA-Seq Library Prep Kit (Illumina) was used to create the library after the total RNA samples were enriched by oligodT (rRNA removal). Using an Illumina NovaSeq 6000 sequencer, the generated library was first identified using an Agilent 2100 Bioanalyzer. The ShuPu Biotechnology company carried out RNA-seq (Shanghai, China). Gene ontology (GO) and Kyoto Encyclopedia of Genes and Genomes (KEGG) pathway annotation was carried out using Cluster Profiler (version 3.16) on differentially expressed gene (DEGs) (*Supplementary 1*, and *Supplementary 2*) [14].

### 2.4. Construction of Protein–Protein Interaction (PPI) Network and Screening of Modules

DEGs were searched in STRING platform version 11.5 (http://string-db.org/cgi/input.pl). A score (median confidence) > 0.9 was set as the cutoff criterion for the selected PPI. The PPI network was constructed using Cytoscape software version 3.9.1 (http://www.cytoscape.org/). The MCODE [15] was used to analyze the modules of the PPI network with the default parameter settings (degree cutoff = 2, node score cutoff = 0.2, k-core = 2, max. depth = 100, *etc.*).

### 2.5. Immunofluorescence Assay

Iliac bone tissue was collected and fixed in 4% paraformaldehyde for 24 hr or overnight. Then, it was placed in EDTA (pH 7.4, 10%, or 0.5 m) for 21 days and embedded in paraffin. One of the sections was selected for immunofluorescence staining of CGRP-associated receptors, and sections were stained with anti-CRLR (ab84467, Abcam) overnight at 4°C using a standard protocol. Then, the corresponding secondary antibodies were added to the sections for 1 hr while protected from light. Sections were restained with 4′,6-diamidino-2-phenylindole (DAPI, Invitrogen). Sample images were observed and captured by fluorescence microscopy (Nikon, Japan).

### 2.6. Isolation and Culture of Rat BMMSCs Primary Cells and Cell Passages

Male SD rats were dislocated and executed at 4 weeks, soaked in 75% alcohol for 10 min, and placed on an ultraclean table after UV lamp disinfection. The femur and tibia of the rats were separated with sterile surgical instruments, the blood was washed with sterile PBS, the bilateral epiphyses were cutoff with sterile scissors, the culture medium was extracted with a 10 ml syringe to rinse the bone marrow cavity, and the bone marrow was washed out completely in a 50 ml centrifuge tube for 5 min. The cell suspension was centrifuged at 1,000 rpm for 5 min, and the supernatant was discarded. The cells were resuspended with culture medium, repeatedly blown into a single cell suspension, and then inoculated into a 10 cm culture dish and incubated in a 37°C, 5% CO_2_ incubator. Cell colonies were formed until the cell density reached 80%–90%, and then the cells were passaged to the 3rd generation (P3 generation) for use in the experiment.

### 2.7. Subcutaneous Osteogenesis in Nude Mice

P3 generation rat BMMSCs (rBMMSCs) were inoculated into culture dishes and cultured to about 80% fusion, and the test group was switched to culture with osteogenic induction solution containing CGRP (10^−8^ M), while the control group was cultured with osteogenic induction solution only. Nude mice were loaded with 1 × 10^7^ cells per material.

### 2.8. Alkaline Phosphatase Staining and Semiquantitative Analysis

P3-generation rBMMSCs were inoculated in 24-well plates at 2 × 10^4^ cells/well, and the cells were walled and divided into two groups for osteogenesis induction. An Alkaline Phosphatase (ALP) kit (Biyuntian, Shanghai) was stained, its absorbance value at 405 nm was measured, and the ALP activity was calculated as OD/mg of total protein. In the other part, a BCA kit (Biyuntian, Shanghai) was used to detect OD values of the samples at 562 nm to calculate the protein concentration.

### 2.9. Alizarin Red Staining

P3-generation rBMMSCs were inoculated in 24-well plates at 2 × 10^4^ cells/well, and the cells were walled and divided into two groups for osteogenesis induction. An ALP kit (Biyuntian, Shanghai) was stained, its absorbance value at 405 nm was measured, and the ALP activity was calculated as OD/mg of total protein. In the other part, a BCA kit (Biyuntian, Shanghai) was used to detect OD values of the samples at 562 nm to calculate the protein concentration.

### 2.10. Quantitative Polymerase Chain Reaction Assay

The p3 generation BMMSCs were stimulated with 10^−8^ M CGRP for 7 days using quantitative polymerase chain reaction (qPCR) to assess osteogenesis-related gene expression, and dose dependent manner of CGRP is described in *Supplementary 3*. The GAPDH gene was used as the internal reference gene, and the primers used were ALP, runt-related transcription factor 2 (RUNX2), SMAD1, Wnt 6, *β*-catenin, osteoprotegerin (OPN), and osteocalcin (OCN), with the sequences shown in Table 2.

### 2.11. Western Blot Analyses

Cells for the experimental assay were collected and washed twice with PBS, then the cells were lysed with a mixture of Ripa cell lysate (Biyuntian, Shanghai) and PMSF (Biyuntian, Shanghai) to prepare a lysis solution to extract total cellular protein. Equal amounts of cell lysate were separated on duplicate 8%–10% SDS-PAGE gels (Solebro, China) and transferred to PVDF membranes (Weiao, China). The membranes were blocked with 5% skimmed dry milk at the end of the transfer and incubated with the primary antibody: *β*-actin antibody (CST, USA), RUNX2 antibody (CST, USA), ALP antibody (Abcam, UK), Wnt6 antibody (Abcam, UK), p38 MAPK antibody (CST, USA), and Phospho-p38 MAPK antibody (CST, USA). The average expression levels of target proteins are relative to *β*-actin. All original WBs images are shown in *Supplementary 4*.

### 2.12. Statistical Analysis

The data were analyzed using the GraphPad Prism 7.0 program. All data were presented in the form (mean standard deviation). The difference between the groups was tested using analysis of variance. For multiple comparisons, the Bonferroni correction was applied. Statistical significance was defined as a two-sided *p*-value less than 0.05.

## 3. Results and Discussion

### 3.1. Identification of DEGs

**Figure 1:**
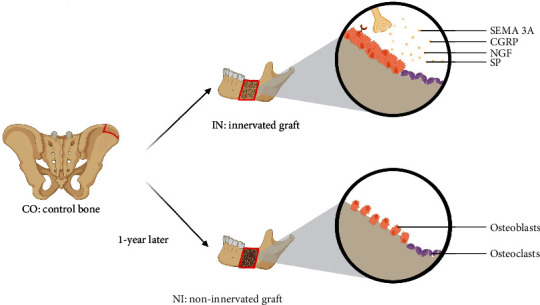
Mechanism of bone resorption prevention through maintenance of bone homeostasis by neurotransmitters in an innervated iliac bone graft model. CO, control bone, IN, innervated graft; NI, and noninnervated graft.

Peripheral nerves contribute to the bone healing and metabolic regulation through the secretion of neurotransmitters such as substance P (SP), NGF, calcitonin gene-related peptide (CGRP), and SEMA3A. Unraveling the mechanisms underlying nervous system-mediated regulation of bone homeostasis is crucial for advancements in bone grafting techniques and the understanding of bone-related diseases (Figure 1). To elucidate the specific physiological functions of innervation in bone, we conducted RNA-seq analysis on samples obtained from CO, IN, and NI. This comprehensive transcriptomic analysis aimed to characterize the impact of innervation on skeletal physiology.

**Figure 2:**
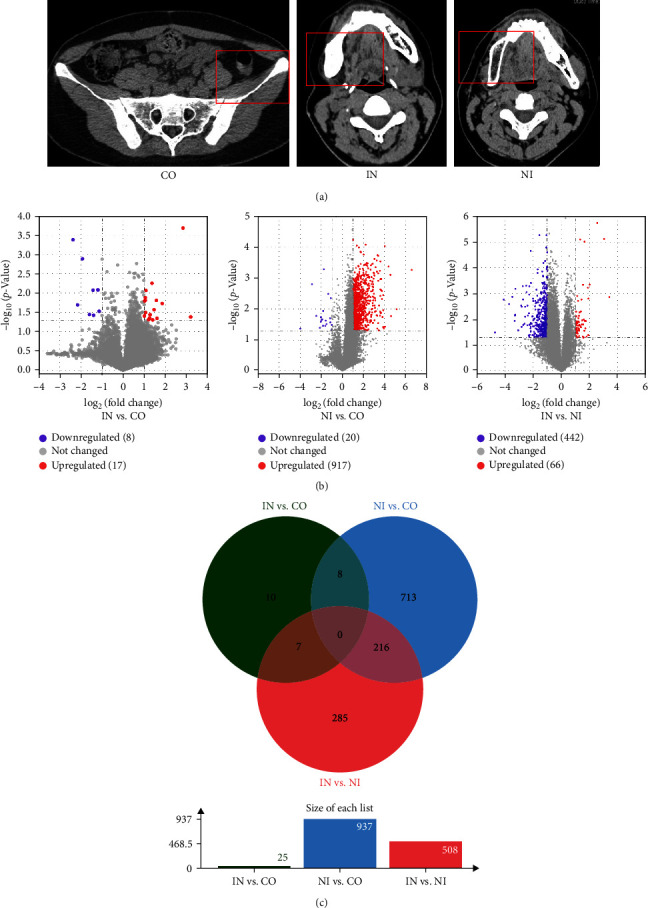
(a) CT findings show that significant bone resorption occurred in NI compared to IN and CO after 1 year. (b) The volcano plot for DEGs of the intercomparison among CO, IN, and NI. (c) Venn diagram of DEGs among CO, IN, and NI.

Our clinical observations have consistently demonstrated that bone graft without innervation invariably leads to the development of osteoporosis in contrast to the cases involving innervation. Notably, innervation appears to effectively preserve the original quality and structural stability of bone grafts (Figure 2(a)). This strongly suggests that innervation plays a pivotal role in maintaining the structural integrity and functional integration of bone grafts. However, a comprehensive understanding of the specific molecular mechanisms underlying this phenomenon is still lacking. To address this knowledge gap, we conducted RNA-seq analysis on bone tissue samples obtained from three distinct groups. The aim was to elucidate the transcriptional landscape of innervation-related processes in the skeletal system. Our transcriptome analysis yielded a total of 632 million paired-end sequence reads from bone tissue samples, with an average of 70 million reads per sample. All genes were filtered based on a log-fold change threshold of ≥1 or ≤−1, with a significance threshold of *p* ≤ 0.05. Surprisingly, only 25 DEGs were identified between IN and CO, including 17 upregulated and 8 downregulated genes. In stark contrast, a substantial number of DEGs were found between NI and CO., with a total of 937 DEGs identified, comprising 917 upregulated and 20 downregulated genes. Furthermore, a comparison between IN and NI revealed 508 DEGs, of which 66 were upregulated and 442 were downregulated. These differential gene expression patterns are visually represented in the volcano plot (Figure 2(b)) and Venn diagrams (Figure 2(c)).

### 3.2. GO and KEGG Analyses

**Figure 3:**
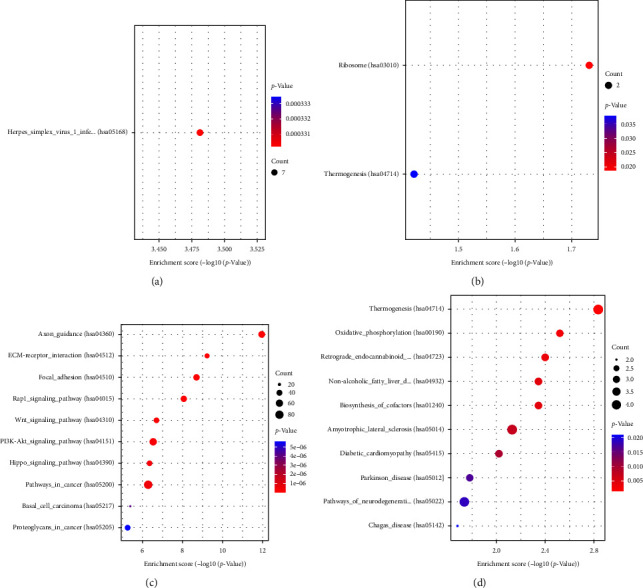
Kyoto Encyclopedia of Genes and Genomes (KEGG) pathway analysis of DEGs among CO, IN, and NI. (a) Upregulation of pathways in IN vs. CO, (b) downregulation of pathways in IN vs. CO, (c) upregulation of pathways in NI vs. CO, and (d) downregulation of pathways in NI vs. CO.

**Figure 4:**
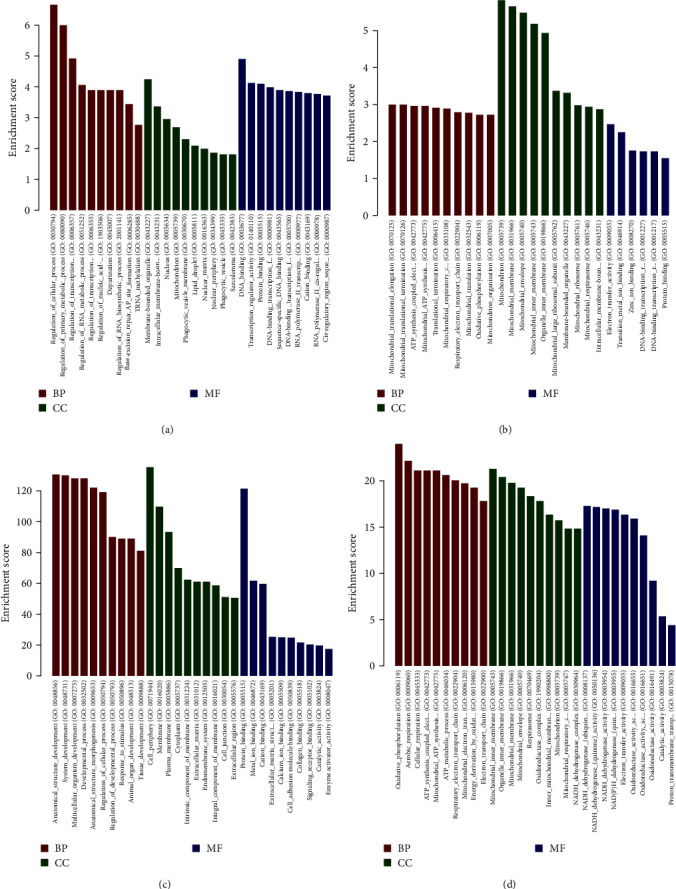
Top five enriched functional gene ontology (GO) analysis of DEGs among CO, IN, and NI. (a) Upregulation of genes in IN vs. CO, (b) downregulation of genes in IN vs. CO, (c) upregulation of genes in NI vs. CO, and (d) downregulation of genes in NI vs. CO. BP, biological process; CC, cellular component; MF, molecular function.

To gain insights into the underlying biological pathways influenced by innervation in bone grafts, we conducted functional enrichment analyses, including GO and KEGG pathway analyses, on the three sets of DEGs. Noteworthy, the KEGG pathway analysis comparing IN and CO revealed only three significantly enriched pathways: ribosome, thermogenesis, and herpes simplex virus 1 infection (Figures 3(a) and 3(b)). These pathways indicate a limited inflammatory response in grafted bones following neural repair. In contrast, the KEGG enrichment analysis of NI vs. CO identified 71 enriched pathways, comprising 16 downregulated and 55 upregulated pathways (Figures 3(c) and 3(d)). These pathways represent a cascade of interactions within the skeletal system that occur after the loss of innervation. They provide molecular insights into the alterations occurring during the process of graft bone nerve reconstruction. To further elucidate the role of innervation in bone grafts, we conducted GO analysis on the identified DEGs. The results highlight numerous molecular signaling pathways that are altered as a consequence of innervation disturbances within the skeletal system (Figure 4). More information about pathway enrichment is shown in *Supplementary 5* and *Supplementary 6*.

### 3.3. PPI Network Analysis and Functional Modules

**Figure 5:**
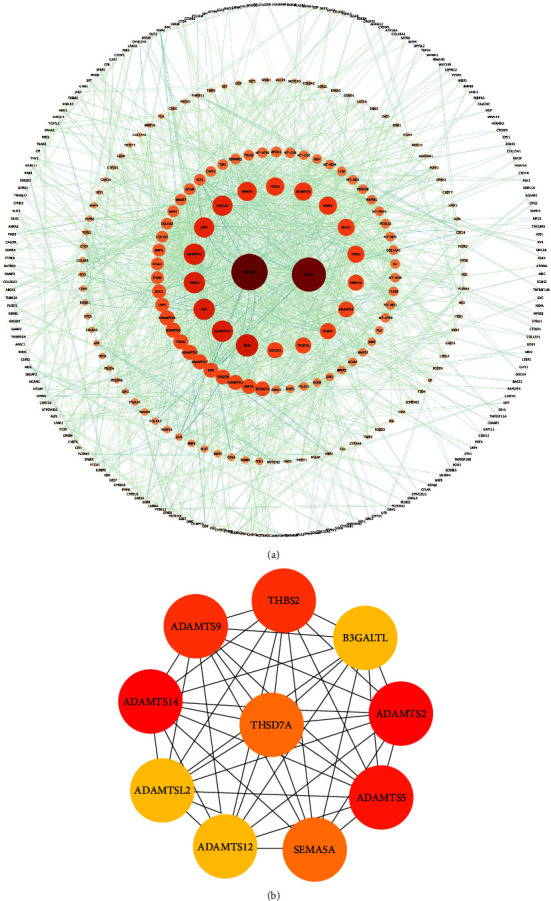
(a) PPI network of NI vs. CO. The higher degree is represented by a redder color. (b) The first 10 genes of the MMC method were chosen using the CytoHubba plugin. The more forward ranking is represented by a redder color.

To gain further insights into the molecular mechanisms underlying innervation in bone, we constructed a network consisting of 369 nodes and 1,704 edges for the comparison between NI and CO (Figure 5(a)). Subsequently, we applied the MCODE algorithm to analyze the entire PPI network, resulting in the identification of 22 modules (Table 3). By employing the maximum neighborhood component (MCC) approach and the CytoHubba plug-in, we selected the top 10 genes in the network based on their importance in the network topology. The selected genes, ranked in the following order, were: ADAMTS14, ADAMTS2, ADAMTS5, THBS2, ADAMTS9, SEMA5A, THSD7A, B3GALTL, ADAMTSL2, and ADAMTS12 (Figure 5(b)). Integrating the results from MCODE and CytoHubba analyses, we identified 10 hub genes that are likely to play crucial roles in innervation-mediated processes: ADAMTS14, ADAMTS2, ADAMTS5, THBS2, ADAMTS9, SEMA5A, THSD7A, B3GALTL, ADAMTSL2, and ADAMTS12. Moreover, to explore the signaling factors involved in the neural maintenance of bone homeostasis, we examined the gene functions within these modules and successfully identified two modules related to neurotransmitter signaling: the CGRP receptor-related module (RAMP2, CALCRL, and RAMP3) and the semaphorin receptor-related module (NRP2, PLXNA3, and PLXNA2).

### 3.4. Osteogenic Differentiation of BMMSCs under CGRP Stimulation

**Figure 6:**
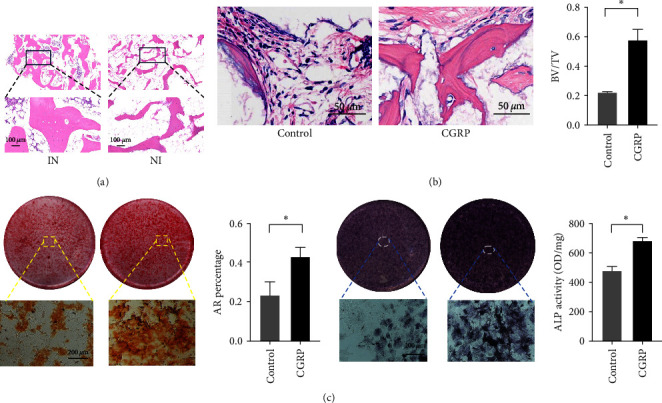
(a) HE staining of iliac bone sections in IN and NI groups, (b) the *in vivo* osteogenic effect of rBMMSCs with CGRP treated subcutaneously transplanted to nude mice (HE staining and BV/TV);  ^*∗*^*p* < 0.05, (c) effects of osteogenic differentiation of rBMMSCs cultured with CGRP detected by ALP assay (ALP staining and ALP semiquantitative analysis) and Alizarin red assay (Alizarin red staining and percentage of calcium nodule in ARS).

**Figure 7:**
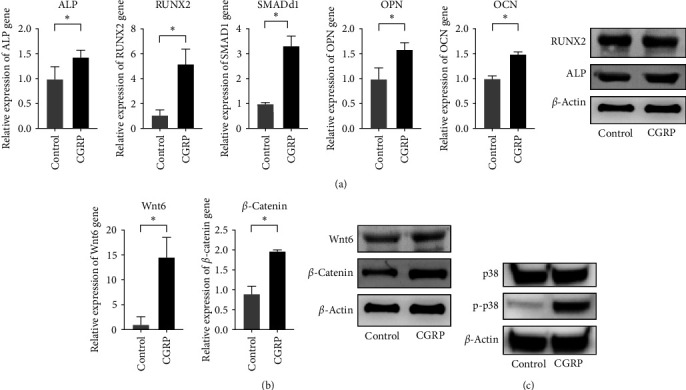
(a) The expression levels of osteogenic-related genes of rBMMSCs detected by PCR. The expression levels of RUNX2 and ALP proteins of rBMMSCs after CGRP stimulation detected by western blot; (b) expression levels of Wnt pathway-related genes and proteins in rBMMSCs treated with CGRP. The gene expression of Wnt6 and *β*-catenin by PCR ( ^*∗*^*p* < 0.05). Expression levels of Wnt6, *β*-catenin protein, and *β*-actin proteins by western blot; (c) western blot showing the phosphorylation of p38 MAPK pathway components in rBMMSCs treated with CGRP;  ^*∗*^*p* < 0.05.

An imbalance in bone homeostasis is the main reason for the development of osteoporosis. Vascularization of the transplanted bone is one of the key factors influencing postoperative absorption. And we performed hematoxylin and eosin (HE) staining on the IN and NI groups and successfully observed a significant number of vascular systems within the transplanted bone. This indicates that the transplanted bone underwent early in-situ healing rather than a process of creeping substitution associated with vascularized bone grafting (Figure 6(a)). Our previous bioinformatics result suggest that deficiency of the neurotransmitter CGRP is one of the main causes of osteoporosis. Decreased bone formation with denervation is almost certainly a consequence of the impaired osteoblast differentiation of BMMSCs. To compare the *in vivo* ectopic osteogenic ability of CGRP-stimulated BMMSCs after 14 days of stimulation, we inoculated them subcutaneously in nude mice. The HE staining results showed that the CGRP-stimulated stem cells exhibited stronger (*p* < 0.05) osteogenic ability after 8 weeks (Figure 6(b)). Meanwhile, we found that CGRP-stimulated rat BMMSCs (BMMSCs) showed higher ALP activity and higher production of calcium nodules with statistical significance (*p* < 0.05), which indicates the effect of CGRP on promoting osteogenic differentiation of BMMSCs (Figure 6(c)). BMMSCs osteogenic differentiation is a crucial event in new bone formation and bone quality maintenance. Osteoblasts synthesize and secrete a variety of bone-specific marker proteins, including ALP, RUNX2, OCN, and OPN, which play important roles in the maturation and biological function of osteoblasts and are responsible for regulating the expression of genes specific to the osteoblast phenotype. The bone morphogenetic protein (BMP) signaling pathway is one of the crucial pathways responsible for osteoblastic bone formation and SMAD1 is a key signal transducer in this pathway. Our experiment demonstrated that the expression levels of RUNX2, SMAD1, OPN, and OCN were significantly increased by the stimulation of CGRP. RUNX2 and SMAD1 were increased by 5.04 ± 1.25 and 3.31 ± 0.41 folds (*p* < 0.05), respectively; and OPN and OCN were increased by 1.58 ± 0.15 and 1.48 ± 0.05 folds (*p* < 0.05), respectively. Based on the results of PCR, we selected RUNX2, which was highly expressed under CGRP stimulation, as the main target gene for the study (Figure 7(a)). The protein expression levels of both RUNX2 and ALP were significantly increased after 7 days of CGRP stimulation, which is consistent with the results of PCR. All the results suggest that CGRP enhances the osteogenic capacity of BMMSCs.

### 3.5. Osteogenic Differentiation of BMMSCs under CGRP Stimulation

**Figure 8:**
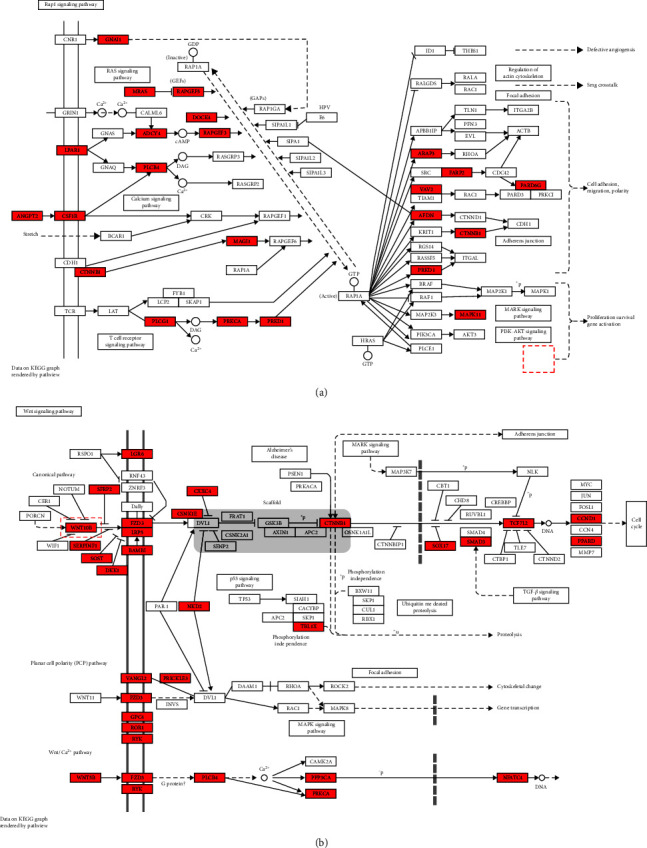
(a) Mapping of Rap1 signaling pathway in NI vs. CO shows that the p38 MAPK pathway is significantly involved. (b) Mapping of the Wnt signaling pathway in NI vs. CO shows that the canonical Wnt pathway is significantly involved in this process. Each gene is colored according to its expression level in the pathway and significantly upregulated genes are marked in red and significantly downregulated genes are marked in green.

**Figure 9:**
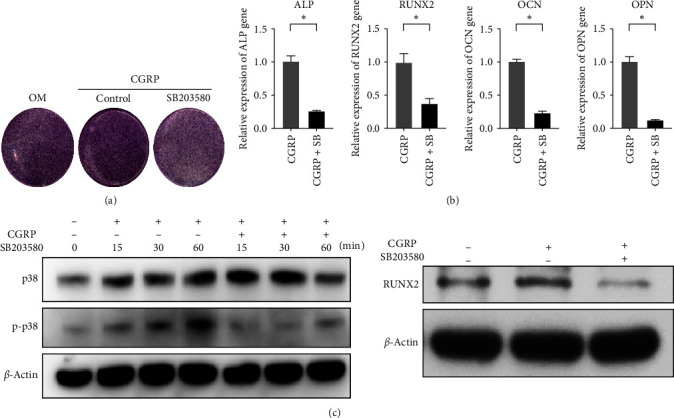
(a) ALP staining showing the changes of the osteogenic effects induced by CGRP after adding p38 inhibitor SB203580 (7 days); (b) the changes of the expression of osteogenic-related genes in rBMMSCs treated with CGRP after adding p38 inhibitor SB203580 by PCR. The expression changes of ALP, RUNX2, OCN, and OPN in rBMMSCs after 7 days' treatment (SB refers to SB203580 ( ^*∗*^*p* < 0.05); (c) expression levels of p38 pathway and osteogenic related proteins in rBMMSCs treated with CGRP after adding p38 inhibitor SB203580 by western blot. The phosphorylation levels of the p38 MAPK pathway and expression levels of RUNX2 protein with or without SB203580.

The results of the KEGG pathway analysis revealed a close association between osteoporosis following bone grafting and the Rap1 and Wnt signaling pathways. To gain a deeper understanding of these pathways, we conducted further annotation and analysis of the DEGs within them. We observed that the genes were primarily involved in the Wnt/*β*-catenin canonical signaling pathway and the p38 MAPK pathway, which is downstream of the Rap1 pathway (Figures 8(a) and 8(b)). Specifically, in the Wnt/*β*-catenin pathway, Wnt6 emerged as a potent endogenous regulator of mesenchymal stem cell fate, stimulating osteoblastogenesis through a *β*-catenin-dependent mechanism. Therefore, we selected Wnt6 for validation. Our experimental findings demonstrated that the expression levels of Wnt6 and *β*-catenin in rBMMSCs significantly increased after 7 days of CGRP stimulation. PCR results showed that the expression levels in the CGRP group were 14.60 ± 4.02 and 1.97 ± 0.02 times higher than those in the control group, respectively (*p* < 0.05). Western blot (WB) analysis corroborated these findings, indicating enhanced protein expression levels of Wnt6 and *β*-catenin after CGRP stimulation (Figure 7(b)). Rap1, a small GTPase acting as a cellular switch, may regulate osteoblast differentiation through ERK and p38-mediated signaling pathways. In our study, p38 MAPK, a downstream pathway of Rap1, was significantly enriched in nerve loss-induced osteoporosis. Hence, it is reasonable to deduce that CGRP promotes osteogenic differentiation of rBMMSCs primarily by activating the p38 MAPK pathway. This deduction is supported by the significantly increased levels of phosphorylated p38 protein (p-p38) observed in the control group after 1 hr of CGRP stimulation (Figure 7(c)). Furthermore, we inhibited the p38 pathway by adding SB203580, a specific inhibitor of this pathway, while maintaining CGRP stimulation, and investigated the osteogenic differentiation of rBMMSCs through ALP staining, PCR, and WB analysis. After 7 days, ALP staining was more pronounced in the samples with CGRP stimulation compared to those without. However, under the same osteogenic induction solution containing CGRP, the ALP staining effect was statistically significantly lighter than that of the control group without the inhibitor and even lighter than that of the osteogenic induction solution group without CGRP stimulation. This suggests that the osteogenic effect of CGRP is achieved by activating the p38 pathway, which serves as an important mediator of osteogenic differentiation, although its effects may extend beyond those induced by CGRP alone. Similarly, after 7 days of CGRP stimulation, the expression levels of osteogenic genes ALP, RUNX2, OCN, and OPN were significantly (*p* < 0.05) decreased when the p38 pathway was inhibited, with relative expressions of 0.25 ± 0.01, 0.37 ± 0.09, 0.23 ± 0.03, and 0.12 ± 0.03, respectively, compared to the samples without the inhibitor. These findings indicate that CGRP increases the expression of ALP, RUNX2, OCN, and OPN through the p38 pathway to promote osteogenic differentiation of rBMMSCs. Furthermore, the expression levels of phosphorylated p38 (p-p38) increased at 15, 30, and 60 min after CGRP stimulation, with the levels of phosphorylation increasing over time. RUNX2 expression also increased after 7 days of CGRP stimulation but was inhibited by the p38 pathway inhibitor, suggesting that CGRP promotes RUNX2 expression through the p38 pathway (Figure 9).

## 4. Discussion

Traditional approaches for repairing maxillofacial bone defects using nonvascularized free bone grafts often result in significant bone resorption and surgical failures, even when the grafts are adequately vascularized. However, our research team has observed that restoration of neural innervation in the transplanted bone can effectively maintain bone mass and preserve the original strength of the graft [12, 16]. While autologous bone grafting remains the current gold standard, emerging neovascularized tissue-engineered bone technology offers promising prospects. Previous studies have clarified the importance of vascularization, material structure, and mechanical stimulation on tissue-engineered bone. However, the specific molecular mechanisms of neutralization of the bone system, another key influence, are still not fully understood [5, 17]. Therefore, an in-depth investigation of the effects of neuralization on tissue-engineered bone and the associated molecular mechanisms remains a cutting-edge research direction in this field. The maintenance of bone tissue relies on the dynamic balance between osteoblasts and osteoclasts, with neurotransmitters playing a crucial role as essential components of the stem cell microenvironment. Exploring the complex interplay between neural components and bone tissue can enhance the design and optimization of innovative strategies for the tissue engineering approaches. In this study, we aim to elucidate the role of neural innervation in the transplanted bone at the transcriptional level by utilizing RNA-seq, a powerful tool for comprehensive analysis of the global transcriptome [18–20].

Through bioinformatic analysis, we identified a limited number of DEGs, specifically 25 DEGs, when comparing IN and CO. However, a larger number of DEGs, 937 in total, were found when comparing NI and CO. These findings underscore the significant contribution of innervation loss to osteoporosis at the transcriptional level. To gain further insights into the mechanistic role of innervation in bone, we conducted a KEGG enrichment analysis of the DEGs identified in the NI vs. CO comparison. Furthermore, among the top 10 upregulated pathways, five were associated with environmental information processing, indicating the involvement of nerves in maintaining bone metabolism through neurotransmitter release. In our study, we selected the top two pathways in the signaling pathway for further investigation. Moreover, we also discovered two neurotransmitters CGRP and SEMA with important potential roles in maintaining bone homeostasis through the PPI network. Notably, previous findings have demonstrated a close association between the Wnt/*β*-catenin pathway and cellular aging [21]. Meanwhile, in our experiments, we observed denervation leads to the “aging” of BMMSCs, characterized by reduced osteogenic differentiation, enhanced adipogenesis, and diminished self-renewal capacity, ultimately resulting in an imbalance in bone homeostasis. Mitochondrial dysfunction, cellular senescence, stem cell failure, and altered intercellular communication are all recognized indicators of aging, which align with the physiological properties of denervation-induced “aging” [22]. It is plausible that denervation may lead to alterations in other aging markers, including epigenetic modifications [23]. In aged mice, a significant decrease in CGRP levels was observed, whereas administration of CGRP showed a marked enhancement of bone formation and a decrease in the accumulation of adipose tissue in the bone marrow, suggesting that this aging may be associated with CGRP [24]. Our experiments demonstrate that the neuropeptide CGRP inhibits the “aging” of BMMSCs and enhances their osteogenic differentiation. The GO analysis comparing NI and CO samples revealed functional abnormalities in NADH dehydrogenase (ubiquinone) activity, NADH dehydrogenase (quinone) activity, NADH dehydrogenase, etc., and impaired activation of enzymes associated with mitochondrial aerobic respiration. These downregulated genes are primarily involved in ATP production, such as the tricarboxylic acid cycle, the respiratory chain, and oxidative phosphorylation. The downregulation of these key genes may directly reduce the cellular energy supply, which is considered one of the manifestations of cellular aging.

Activation of genes such as Wnt6 inhibits adipogenesis and promotes BMMSCs to differentiate toward osteogenesis. Chromatin analysis revealed H3K4me3 modifications near the Wnt ligand promoter, leading to Wnt gene repression, which is closely linked to biological functions like cell differentiation. EZH2, a catalytic subunit of polycomb repressor complex 2 (PRC2), acts as a methyltransferase of H3K4me3 and is involved in nonhistone methylation processes. Recent studies have shown elevated EZH2 expression in BMMSCs from ovariectomy-induced osteoporotic mouse models, correlating with their stemness [25]. EZH2 expression is regulated by upstream pathways, including p38 MAPK. Previous research has demonstrated the significant role of the p38 MAPK osteogenic pathway in migration and osteogenic differentiation [26]. Notably, our previous studies have demonstrated that p38 agonists have shown potential in promoting migration and osteogenic differentiation of BMMSCs derived from the neural crest [27, 28]. Integrating the expression profile data into the KEGG pathways, we observed that upregulated genes in the Rap1 signaling pathway mainly concentrated in its downstream p38 MAPK pathway, while upregulated genes in the Wnt signaling pathway were primarily involved in the canonical Wnt/*β*-catenin pathway. Taking into account our bioinformatic findings, which indicate high-CGRP expression in the innervated skeleton, as well as the involvement of the Wnt signaling pathway and p38 MAPK among other key pathways in neural regulation of bone homeostasis, we propose the following hypothesis: CGRP has been implicated in the regulation of the Wnt signaling pathway through its interaction with the p38 MAPK pathway. This regulatory mechanism is believed to play a crucial role in preventing bone aging and maintaining bone homeostasis by modulating the behavior of BMMSCs.

Our subsequent experimental investigations have yielded compelling evidence that CGRP-stimulated bone BMMSCs exhibit augmented osteogenic differentiation activity and capacity. Additionally, we have demonstrated *in vitro* that CGRP can induce phosphorylation of the p38 pathway. Notably, when we specifically inhibited the p38 pathway under CGRP-stimulated conditions, the expression of phosphorylated p38 (p-p38) was significantly suppressed and reduced. Furthermore, the expression of RUNX2, a key transcription factor closely associated with osteogenesis, which is highly expressed during the most active phase of osteogenic differentiation and considered critical for the differentiation of BMMSCs into osteoblasts during bone formation, was also diminished. These findings indicate that CGRP selectively enhances bone formation through the activation of the p38 MAPK pathway. Furthermore, preliminary findings suggest the involvement of Wnt6 in CGRP-stimulated osteogenesis, as evidenced by the upregulation of Wnt6/*β*-catenin expression in CGRP-stimulated rat BMMSCs. Additionally, there appears to be an interaction between the p38 MAPK and Wnt pathways, with previous studies demonstrating that Wnt4 enhances osteoblast differentiation through p38 MAPK-mediated signaling [29]. Thus, it is plausible that Wnt6 may act in coordination with the p38 pathway to regulate the transcription of RUNX2 and promote the expression of osteogenesis-related factors. However, further investigations are warranted to ascertain the precise relationship between the Wnt6/*β*-catenin signaling pathway and the p38 MAPK pathway, specifically whether they are juxtaposed, upstream, or downstream of each other.

CGRP has emerged as a pivotal regulator in the intricate network that governs skeletal physiology. It is widely distributed in bone tissues such as periosteum and bone marrow, and actively participates in the processes of nerve innervation, vascularization, and bone formation during skeletal development. Serving as a multifunctional neuropeptide, CGRP exhibits remarkable pleiotropy within the skeletal system. It plays a crucial role in orchestrating communication between osteoblasts and various other cell types, influencing not only osteogenesis, osteoclastogenesis, and adipogenesis, but also angiogenesis and nurogenesis. The profound anabolic effects of CGRP are indispensable for maintaining bone homeostasis, thereby establishing its indispensable position within the neurovascular-osteogenic regulatory network [30–34]. CGRP possesses the capacity to exert regulatory influence on bone metabolism through its ability to enhance the osteoblast differentiation and impede osteoclastogenesis. *In vitro*, investigations conducted on both animal and human models have consistently demonstrated the inhibitory effect of CGRP on the differentiation of monocytes into osteoclasts [29–31]. The underlying mechanisms involve the elevation of cAMP levels and increased expression of OPG, while concurrently suppressing receptor activator of nuclear factor *κ*B ligand (RANKL), the transcriptional repressor Jdp2, and actin polymerization, which collectively contribute to the inhibition of osteoclast formation [35]. The inhibitory effect of CGRP on osteoclasts has received significant recognition and has become a prominent research area.

Another key neurotransmitter identified in our study, semaphorins, extracellular signaling proteins, play a crucial role in bone formation and maintenance [36, 37]. Similar to CGRP, SEMA3A has a positive influence on sensory neural modulation of bone remodeling. Studies have shown that neuron-specific SEMA3A-deficient mice exhibit a low-bone mass phenotype due to reduced bone formation and increased bone resorption, whereas osteoblast-specific SEMA3A-deficient mice have normal bone formation and bone mass. The reduction of SEMA3A, which innervates bone nerves, is a significant factor contributing to decreased bone formation and increased bone resorption [38]. *In vitro* experiments have demonstrated that SEMA3A can induce the conversion of adipose mesenchymal stem cells to the osteogenic type and promote bone regeneration *in vivo* [39]. Furthermore, SEMA3A stimulates the osteogenic development of rBMMSCs in inflammatory conditions by inhibiting the Wnt/*β*-catenin signaling pathway [40]. Although extensive research has been conducted on CGRP, which has been studied for nearly four decades, investigation on SEMA3A is still in its early stages, requiring additional experimental evidence to unravel its potential mechanisms in bone biology.

Meanwhile, there are some limitations of this study that need to be pointed out. First, in selecting genes from the canonical Wnt signaling pathway, we only chose to validate Wnt6, however, there may be other genes within this pathway that also play similar roles. Meanwhile, our understanding of the intracellular activities between Wnt *β*-catenin -receptor binding and target gene transcription in the canonical Wnt pathway is incomplete. We only selected one gene for validation, and further investigations should aim to improve the comprehensive understanding of the underlying mechanisms involved. We should focus on analyzing the subcellular localization and phosphorylation of *β*-catenin proteins as well as posttranslational modifications and intermolecular interactions in the order of change after the activation of the Wnt pathway [41]. Second, we did not assess the functional roles of other signaling cascades within the MAPK pathway (such as JNK and ERK), despite the absence of significant differences observed in the bioinformatics analysis [42, 43]. Nevertheless, these pathways may still possess latent functions that warrant investigation. Moreover, we did not establish *in vitro* animal models of osteoporosis with nerve deficit, and future studies should incorporate such models to gain a more comprehensive understanding of the relationship between neuromodulation and bone metabolism-related diseases [44]. Furthermore, it is worth noting that in our study, we did not investigate the differentiation of BMMSCs into specific cell subpopulations such as chondrocytes, nerves, and myoblasts. This limitation may have hindered our comprehensive understanding of the complex network involved in innervating osteogenesis. However, we recognize that single-cell sequencing technology can be a powerful tool to address this limitation and provide valuable insights into the homeostatic regulation and specific cell subpopulations that contribute to innervating osteogenesis [45]. Through additional experiments and thorough analysis, we can shed more light on the precise functions and interdependencies of these pathways in neural communication and bone metabolism.

The DEGs data obtained in this study, not only enhance our understanding of the underlying mechanisms of innervation in grafted one, but also offer valuable insights into the roles of innervation in bone diseases and the advancement of tissue-engineered bone. Through bioinformatics analysis, we have successfully validated the positive impact of the neuropeptide CGRP on the differentiation of BMMSCs toward osteogenesis by engaging the p38 MAPK and Wnt6/*β*-catenin signaling pathways. The comprehensive dataset of DEGs obtained in this study significantly contributes to the elucidation of the intricate functional network topology underlying neurogenic crosstalk and bone metabolism-related diseases. It offers valuable insights into the molecular mechanisms that govern the interplay between innervation and bone homeostasis.

## 5. Conclusions

Our multibioinformatics approach reveals key mechanisms involved in the innervation in maintaining the bone homeostasis and demonstrates that innervation enhances the osteogenic capacity of BMMSCs by secreting CGRP to act on their p38 MAPK and Wnt6/*β*-catenin pathways, thereby maintaining bone homeostasis. This study provides new evidence for the mechanisms of bone innervation and subsequent clinical translation.

## Figures and Tables

**Table 1:** Sample information.

Number	Age	Diseases	Gender
CO1	36	Ameloblastoma	Male
CO2	27	Ossifying fibroma	Female
CO3	21	Ameloblastoma	Male
IN1	48	Malignant mesenchymal tumor	Female
IN2	24	Ameloblastoma	Female
IN3	21	Ameloblastoma	Male
NI1	26	Inflammatory myofibroblast tumor	Male
NI2	15	Osteoblastoma	Female
NI3	29	Ameloblastoma	Female

**Table 2:** qPCR primer sequences.

Gene	Forward primer	Reverse primer
GAPDH	CTCTGTGTGGATTGGTGGCT	CGCAGCTCAGTAACAGTCCG
ALP	GGAACGGATCTCGGGGTACA	ATGAGTTGGTAAGGCAGGGT
Wnt6	AGTTTGAGAGGAAGTTGGGATAG	GGTAACCGGTGGAATGAGATAG
*β*-Catenin	TACCGCTGGGACCCTACACAAC	GCGTGGTGATGGCGTAGAACAG
RUNX2	GGACCGACACAGCCATATAAA	GCCTCATTCCCTAACCTGAAA
SMAD1	GGGACTGCCTCATGTCATTTA	AGACTTCCTTCTGCTTGGAAC
OPN	CCAGCCAAGGACCAACTACA	AGTGTTTGCTGTAATGCGCC
OCN	CTGACAAAGCCTTCATGTCCAA	GCGCCGGAGTCTGTTCACTA

**Table 3:** The 22 functional modules from the NI vs. CO PPI network.

Cluster	Score	Nodes	Edges	Node IDs
1	15	15	210	ADAMTS6, THBS2, ADAMTS12, ADAMTS5, ADAMTS14, ADAMTSL2, B3GALTL, ADAMTS9, ADAMTS10, ADAMTS2, THSD1, ADAMTS17, THSD7A, THSD7B, SEMA5A

2	9.778	10	88	MT-ND2, MT-ND4, MT-ND1, MT-ATP8, MT-CO2, MT-CYB, MT-ATP6, MT-ND6, MT-ND5, MT-ND4L

3	8	8	56	ITGAV, ITGA11, ITGA10, ITGA2, ITGA5, JAM2, ITGB5, ITGA8

4	6.333	7	38	SMAD7, BAMBI, BMPR2, BMP2, ACVR1, BMP7, BMP4

5	5	7	30	COL5A1, LUM, PCOLCE, COL5A2, P4HA2, BMP1, COL3A1

6	5	5	20	PLCG1, PDGFRB, PDGFA, PDGFRA, SPHK1

7	4.5	5	18	OAS3, OAS2, IFI6, IFI44, IFI44L

8	4	4	12	FGFR3, SPRY2, SDC2, FGFR2

9	3.333	4	10	FZD8, LRP5, FZD4, SOST

10	3.043	24	70	MMP13, TIMP2, PLAT, MMP1, F2, IGF1, CHST6, SERPINE2, FGA, CDH2, FGG, LIF, OMD, TJP1, HEY1, GJA1, KAT2A, NOTCH3, MMP16, HEYL, WWTR1, HEY2, ACAN, RUNX2

11	3	3	6	LOX, LOXL2, LOXL4

12	3	3	6	ACP5, CTSK, TNFSF11

13	3	3	6	GAS1, BOC, PTCH1

14	3	3	6	RAMP2, CALCRL, RAMP3

15	3	3	6	CFH, CFB, CFI

16	3	3	6	KDELR2, CHN1, KDELR3

17	3	3	6	PER2, CSNK1E, PER3

18	3	3	6	NRP2, PLXNA3, PLXNA2

19	2.8	6	14	EPHA3, EPHA2, EFNB3, EFNA5, MMP2, EPHB4

20	2.667	4	8	CHST7, CHST3, CSPG4, BGN

21	2.667	4	8	CYP2C9, CYP3A4, CYP2C8, CYP2U1

22	2.25	9	18	MMP14, CTGF, LRP1, TWIST1, SNAI1, SMAD3, CD44, HSPG2, TGFB3

## Data Availability

All data generated and analyzed in this study are included in this article.

## Supplementary Materials

Supplementary 1 DEGs of IN vs. CO.

Supplementary 2 DEGs of NI vs. CO.

Supplementary 3 Dose dependent manner of CGRP.

Supplementary 4 Original western blots images.

Supplementary 5 Gene set enrichment analysis of IN vs. CO.

Supplementary 6 Gene set enrichment analysis of NI vs. CO.
