# Supplementary material for: Identification of Functional Modules and Key Pathways Associated with Innervation in Graft Bone—CGRP Regulates the Differentiation of Bone Marrow Mesenchymal Stem Cells via p38 MAPK and Wnt6/β-Catenin
Source: Stem Cells Int. 2023 Aug 16;2023:1154808. doi: 10.1155/2023/1154808 (PMC10447124; doi:10.1155/2023/1154808)
Supplement: Supplementary 3 — Dose dependent manner of CGRP. [file 1154808.f3.docx]

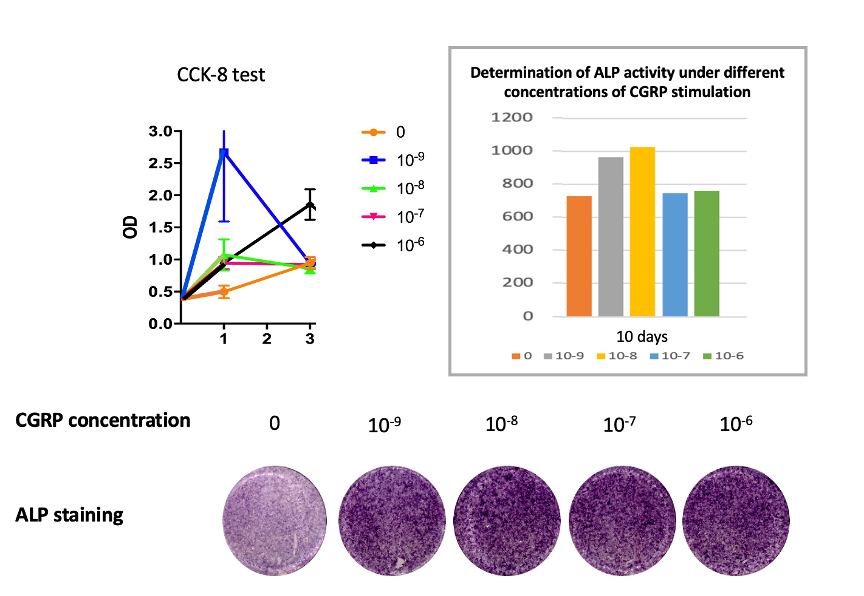


The CCK-8 assay was used to assess the proliferative activity of BMMSCs after 1, 2 and 3 days of stimulation with different concentrations of CGRP. ALP staining was used to assess the osteogenic activity of BMMSCs after 7 days of stimulation with different concentrations of CGRP. The results of the dose-dependent experiments indicated that 10^-8^ M CGRP is the optimal dose to promote the osteogenesis of BMMSCs.
