# Supplementary material for: Identification of Functional Modules and Key Pathways Associated with Innervation in Graft Bone—CGRP Regulates the Differentiation of Bone Marrow Mesenchymal Stem Cells via p38 MAPK and Wnt6/β-Catenin
Source: Stem Cells Int. 2023 Aug 16;2023:1154808. doi: 10.1155/2023/1154808 (PMC10447124; doi:10.1155/2023/1154808)
Supplement: Supplementary 4 — Original western blots images. [file 1154808.f4.docx]

# original western blots images

## Inhibitors runx2


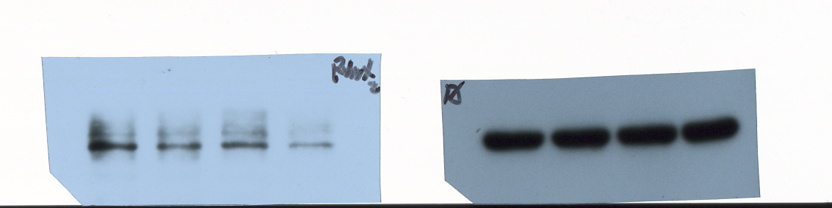


runx2，actin

## Inhibitor p38


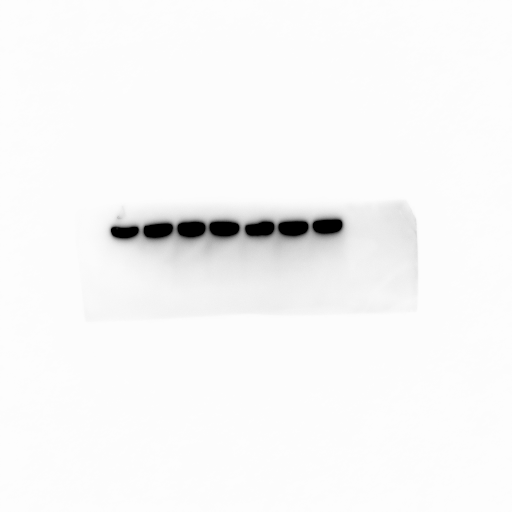


Actin


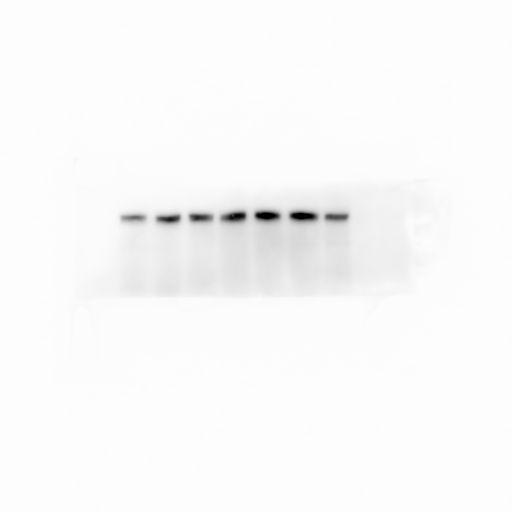


p38


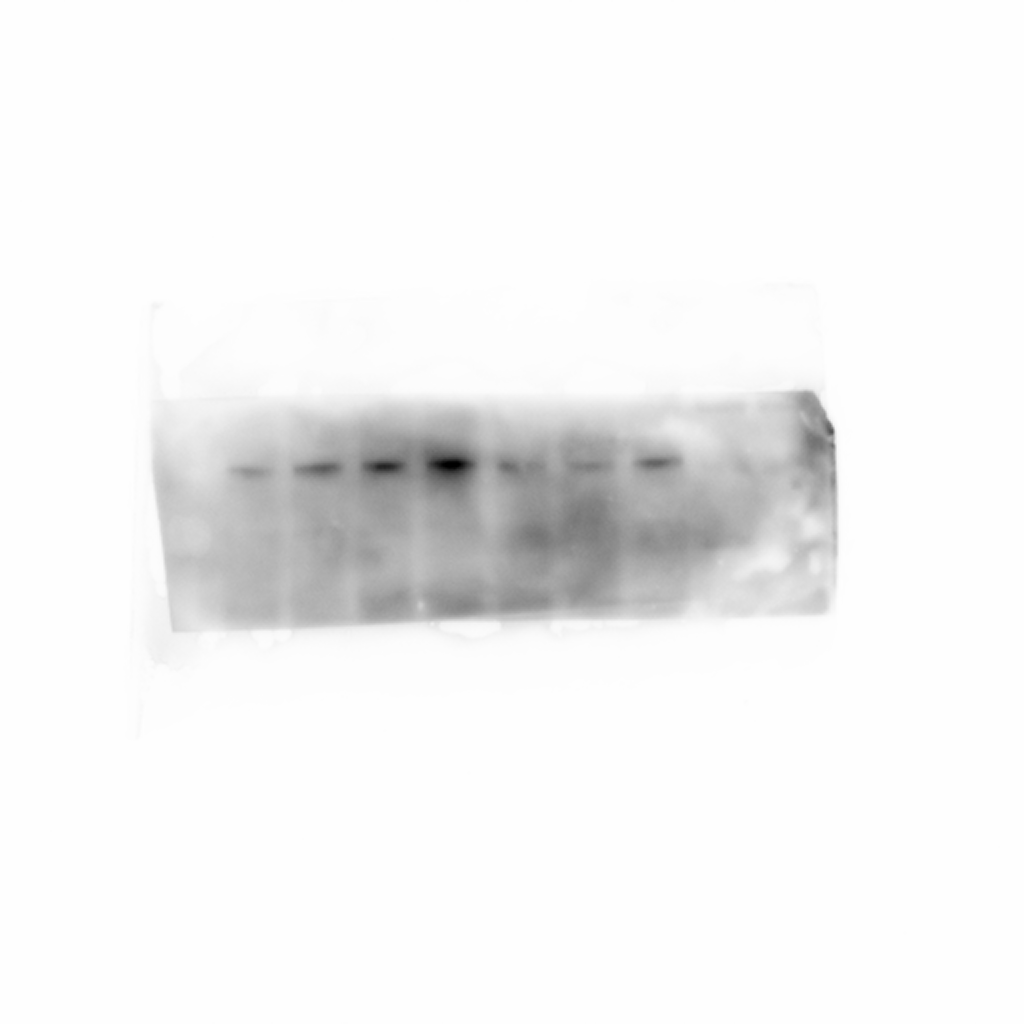


pp38

## p38，p-p38


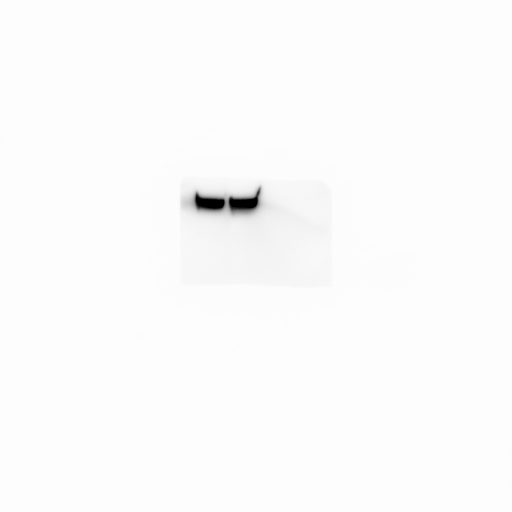


P38

Actin


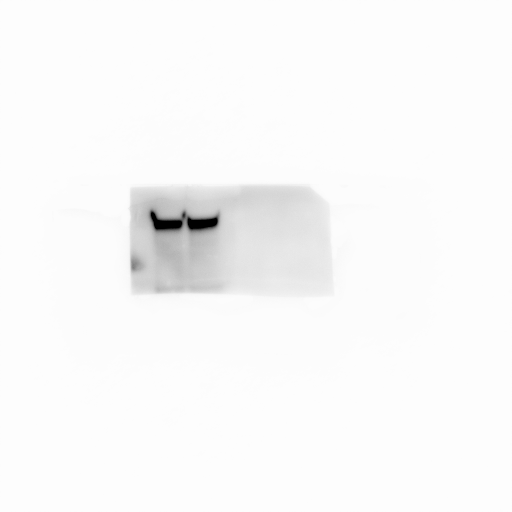


Actin


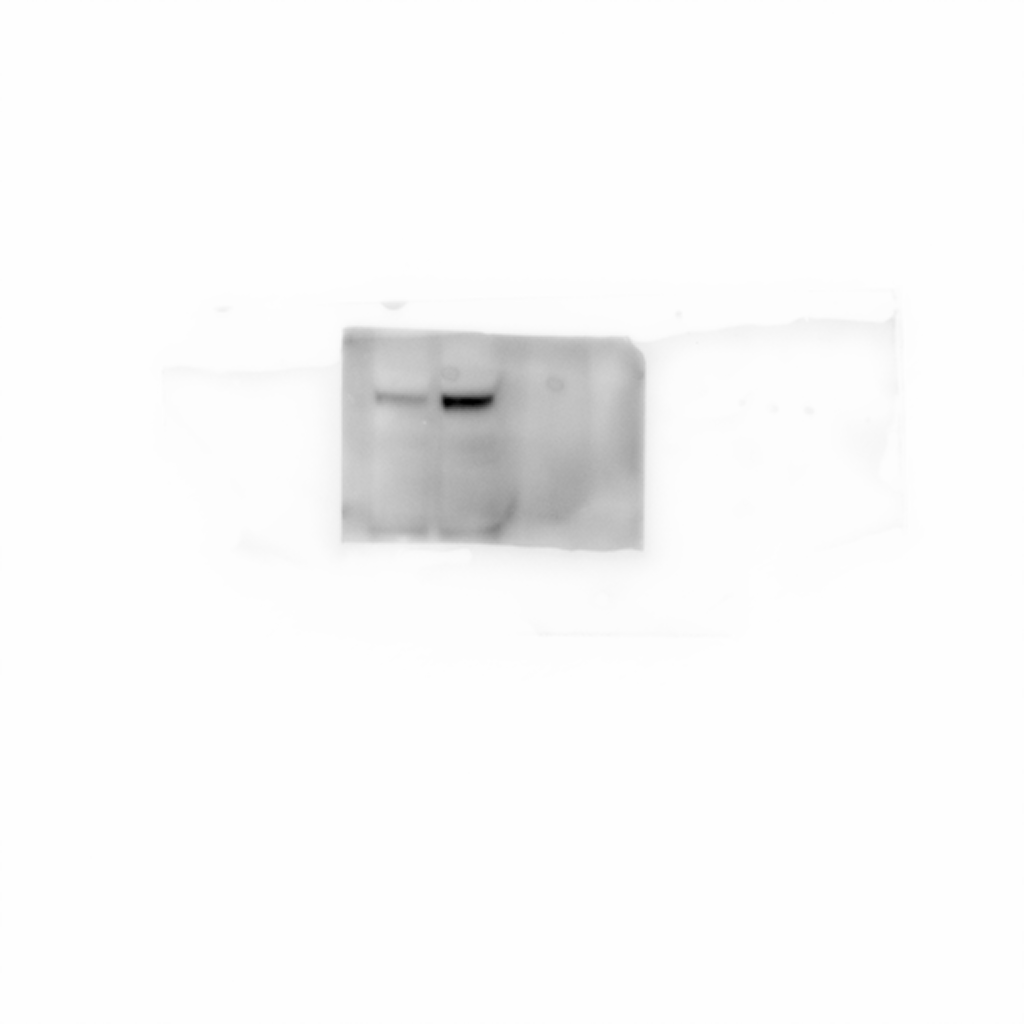


Pp38

## others


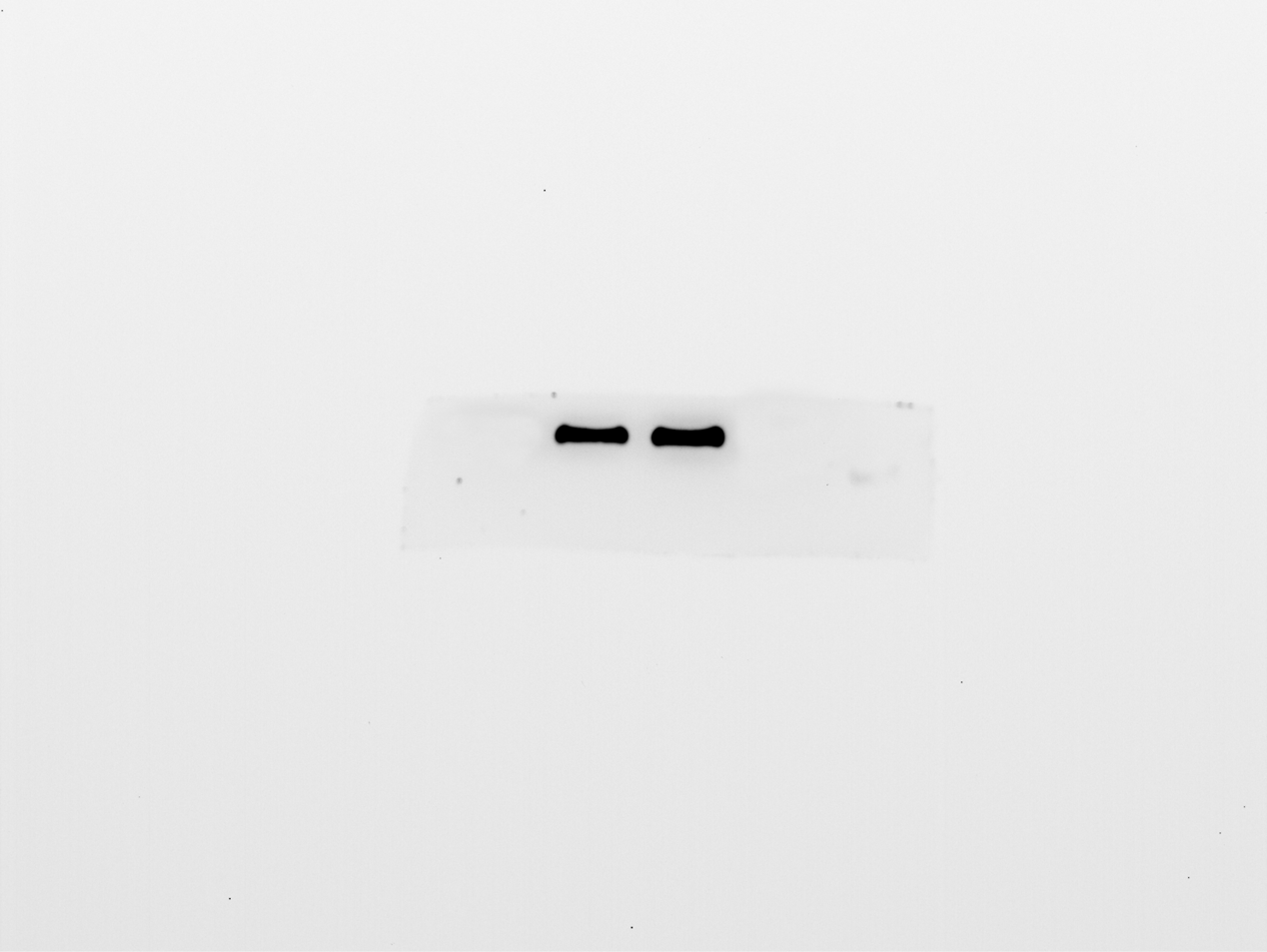


Actin


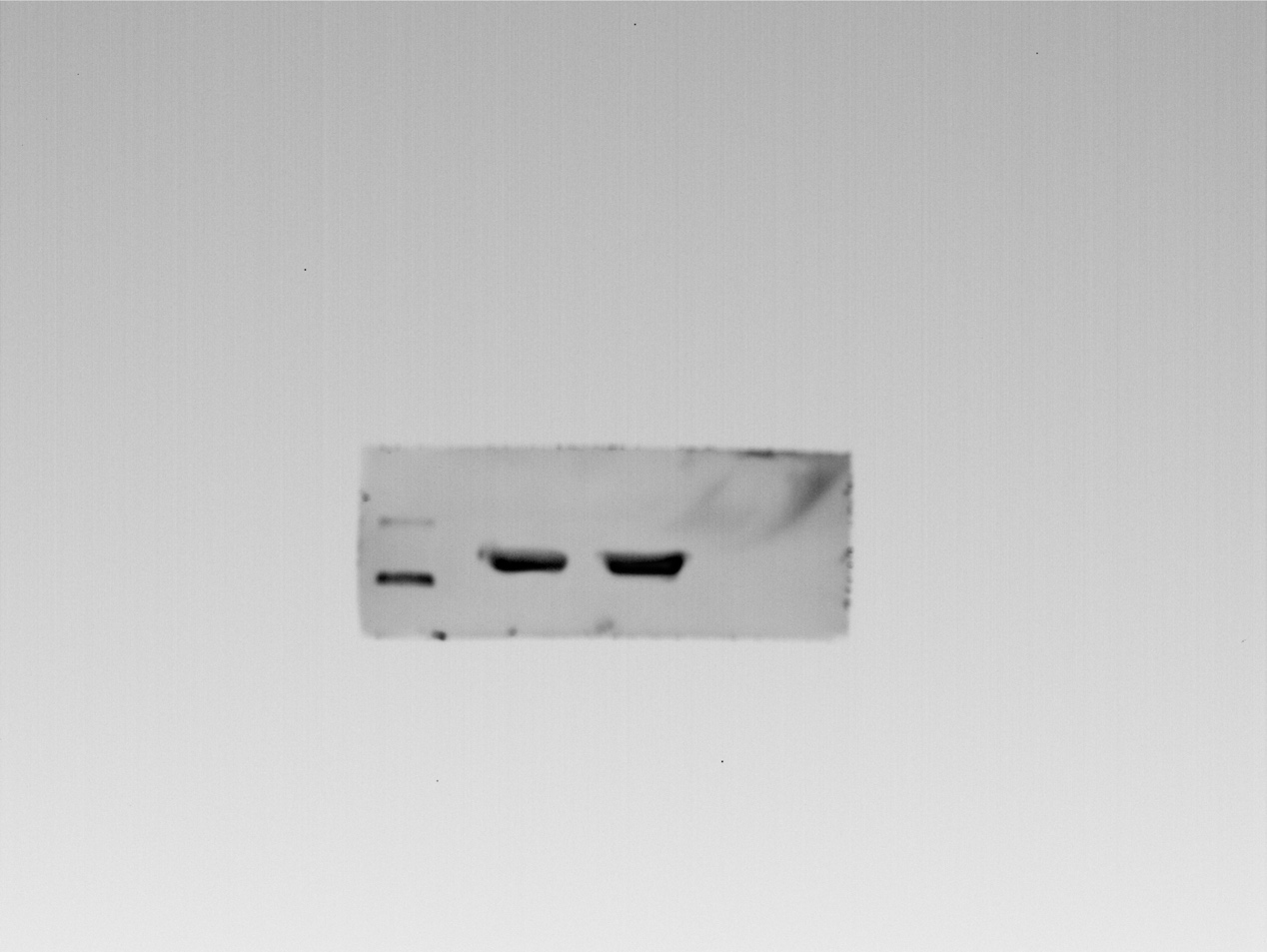


ALP


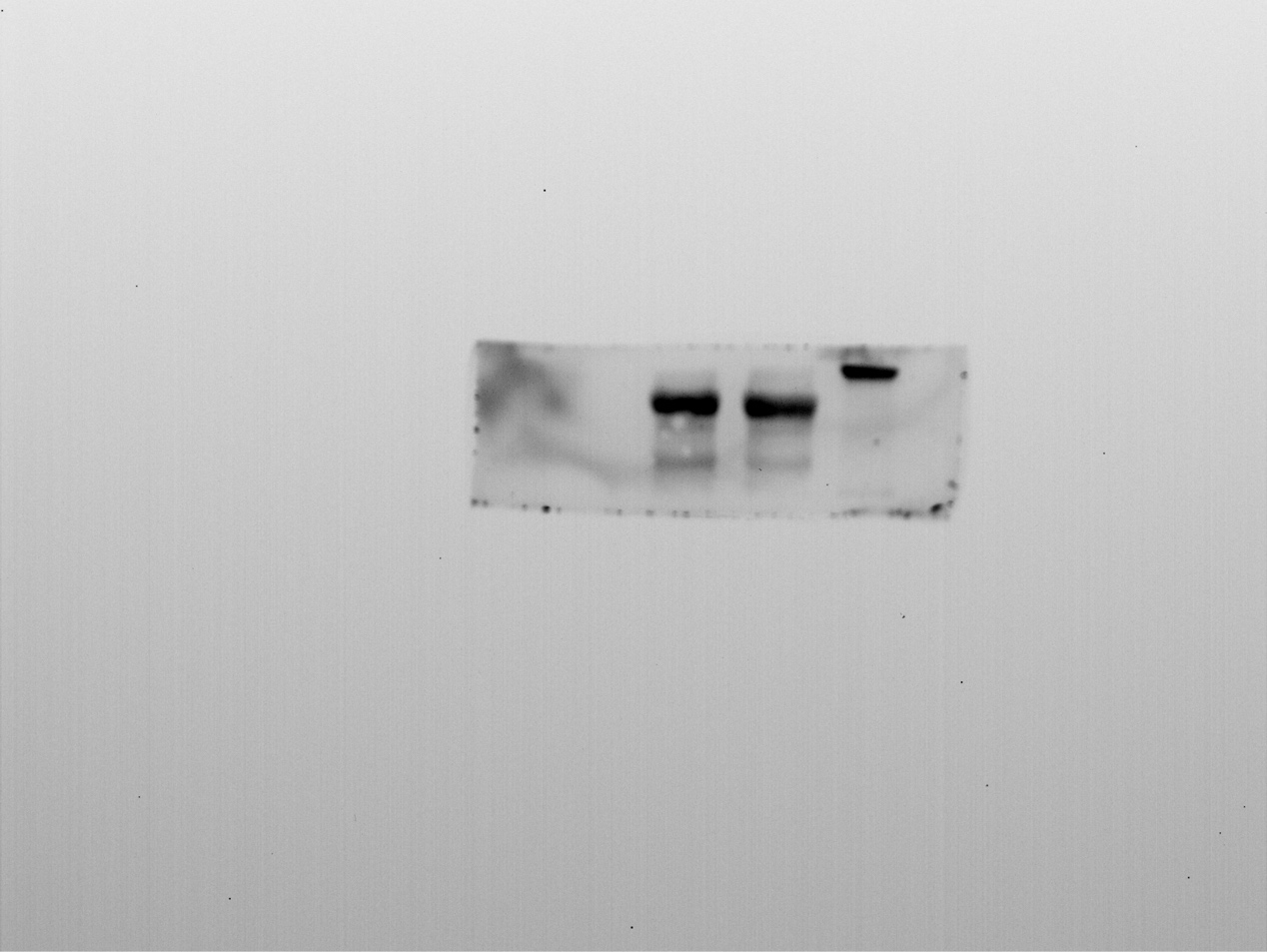
RUNX2


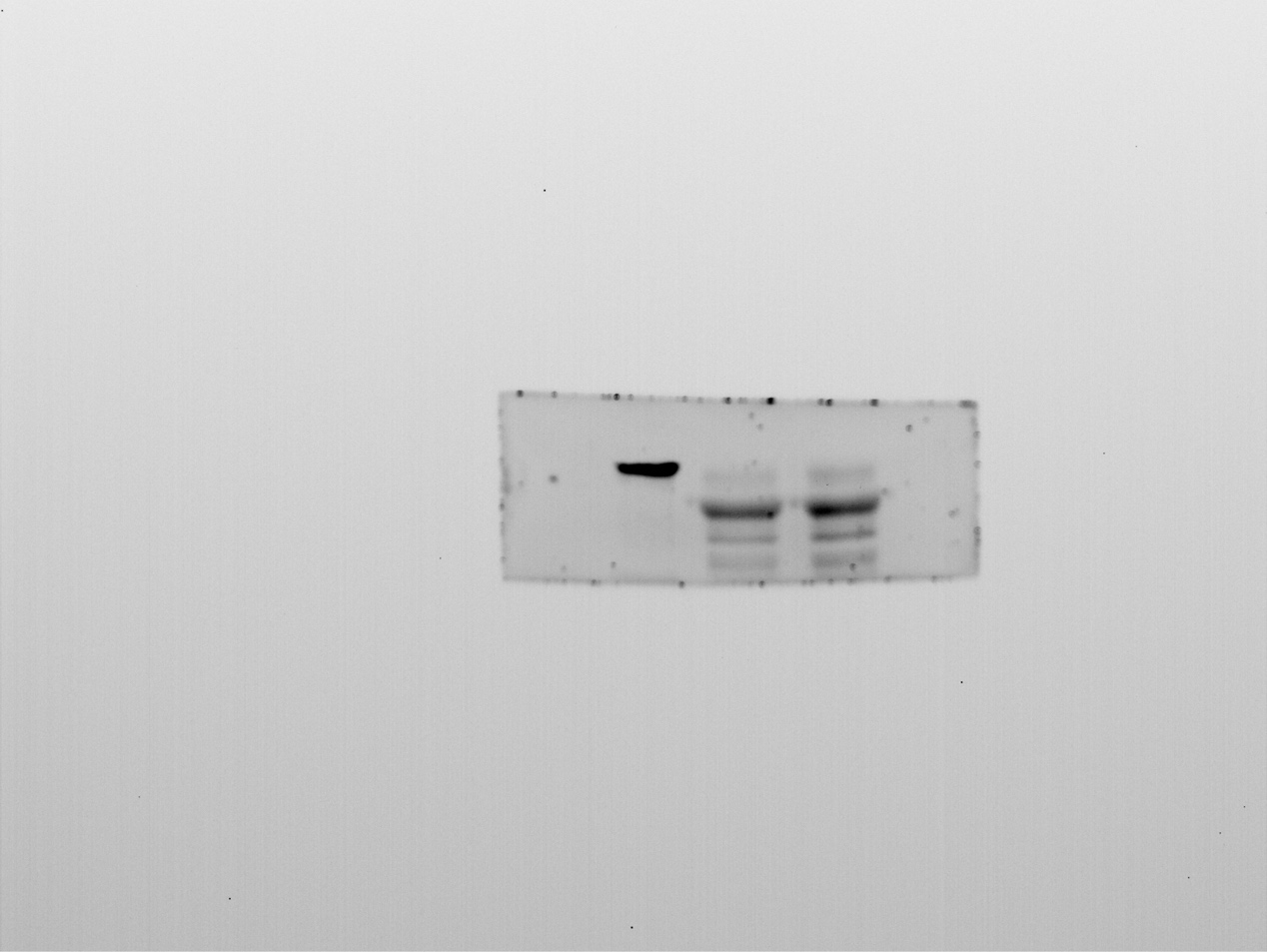


Wnt 6


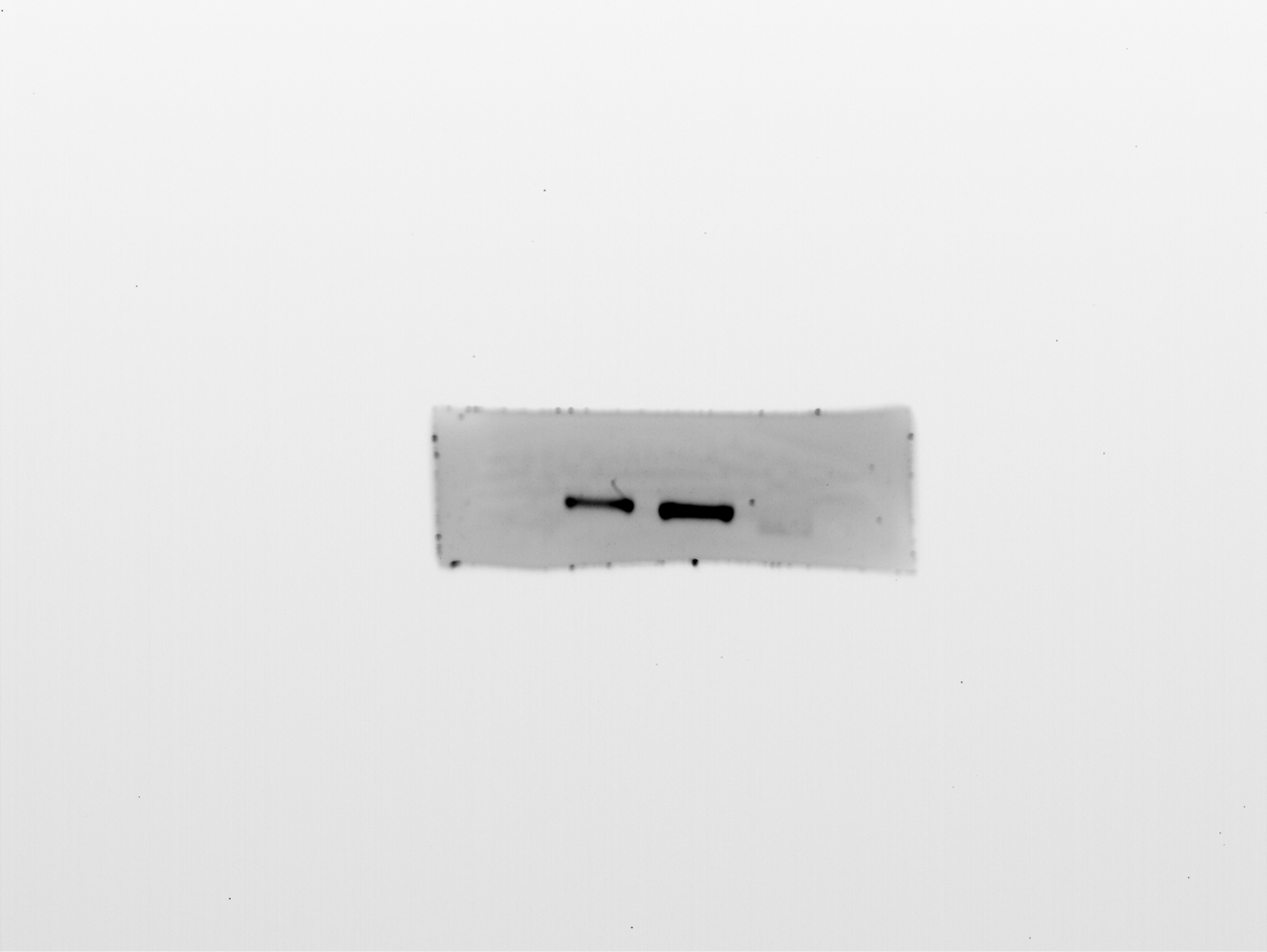


β-Catenin
