# Supplementary figures and images for: Identification of Functional Modules and Key Pathways Associated with Innervation in Graft Bone—CGRP Regulates the Differentiation of Bone Marrow Mesenchymal Stem Cells via p38 MAPK and Wnt6/β-Catenin
Source: Stem Cells Int. 2023 Aug 16;2023:1154808. doi: 10.1155/2023/1154808 (PMC10447124; doi:10.1155/2023/1154808)

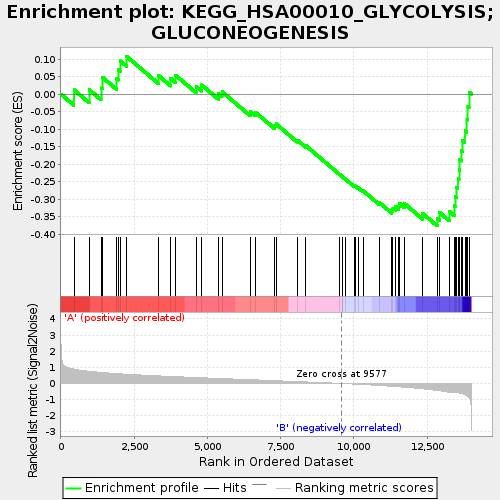

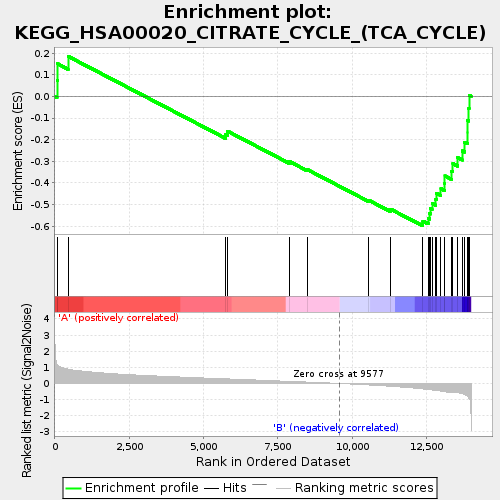

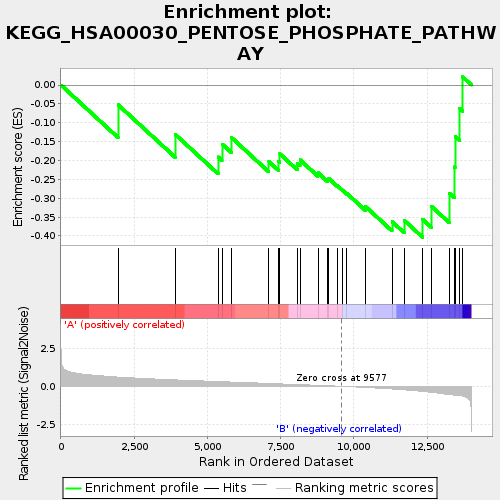

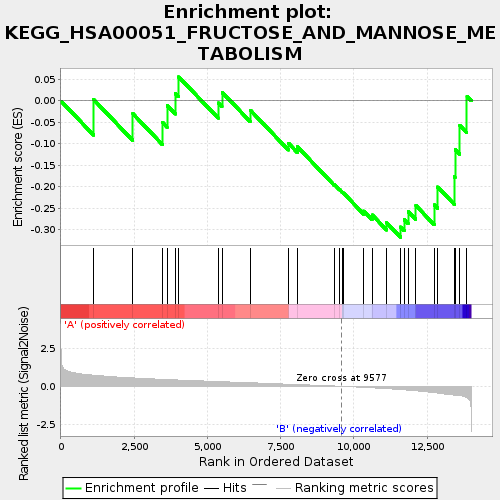

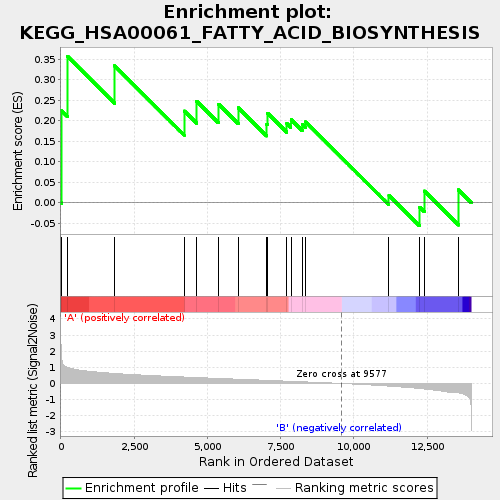

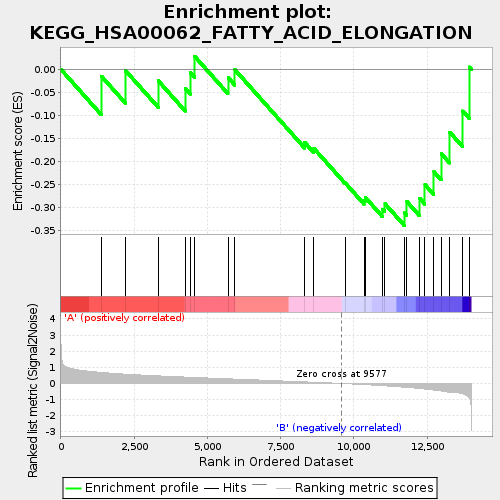

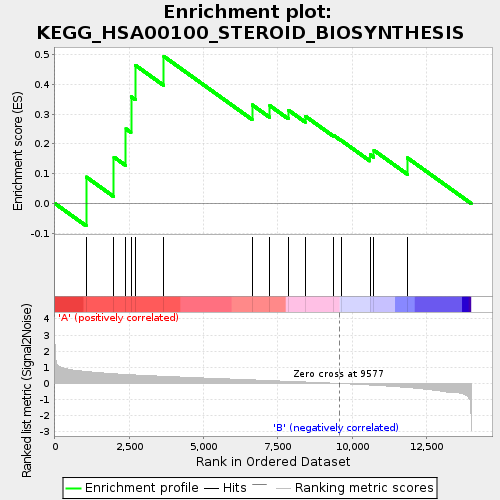

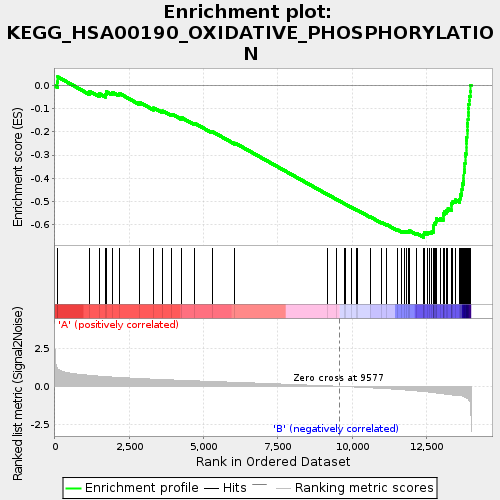

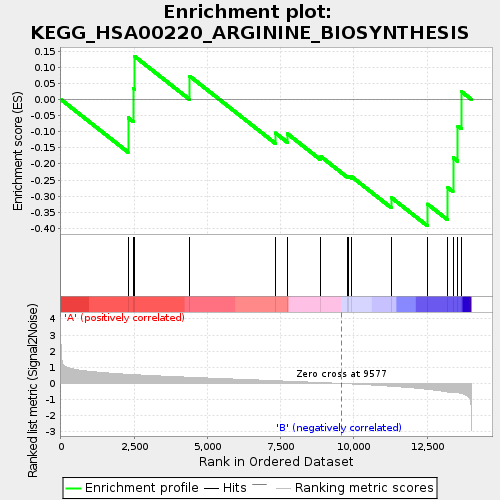

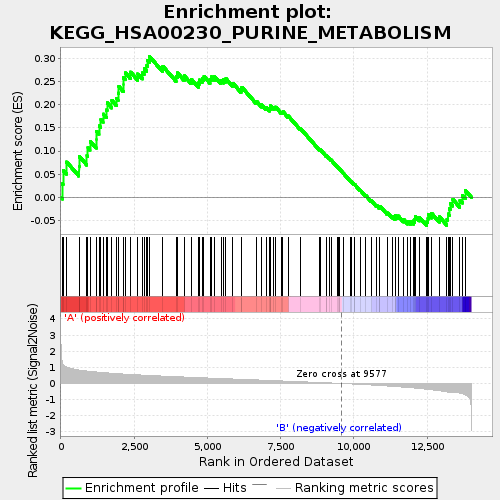

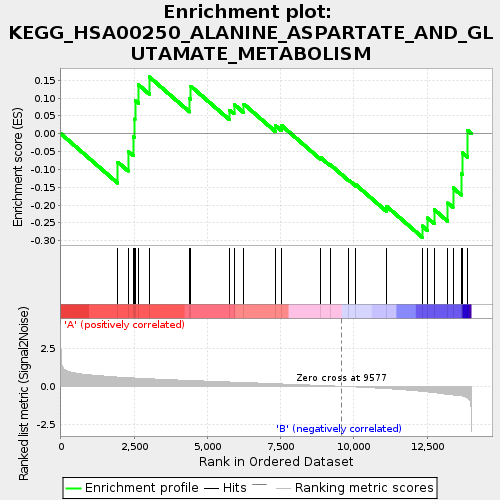

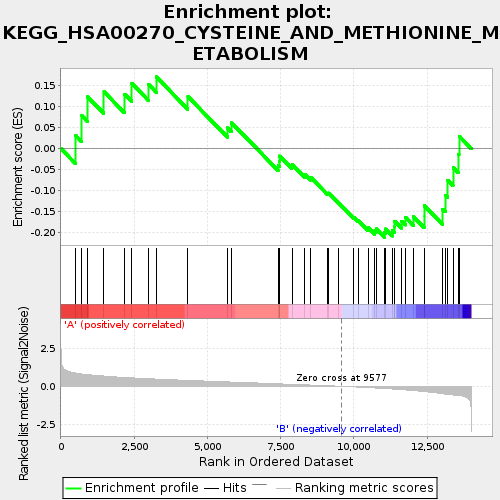

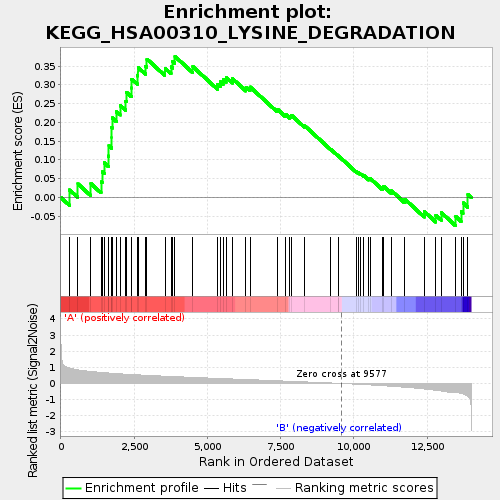

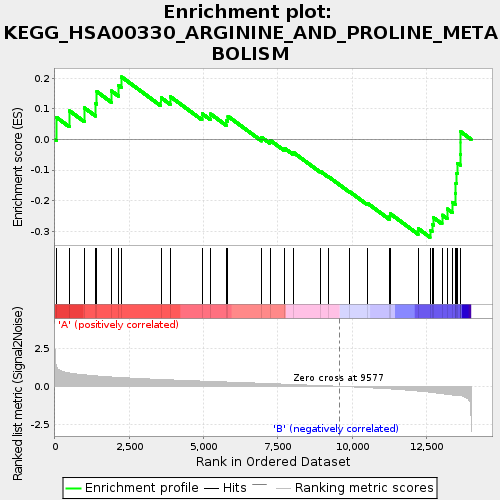

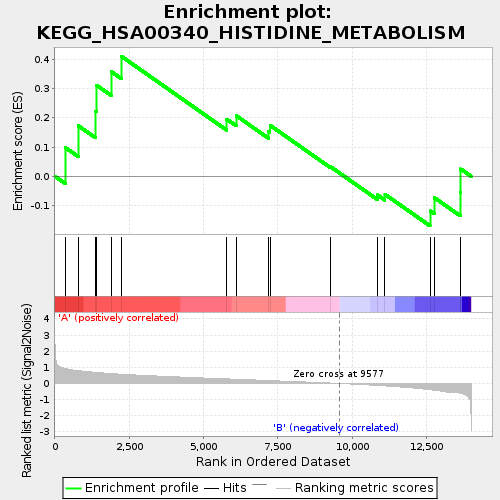

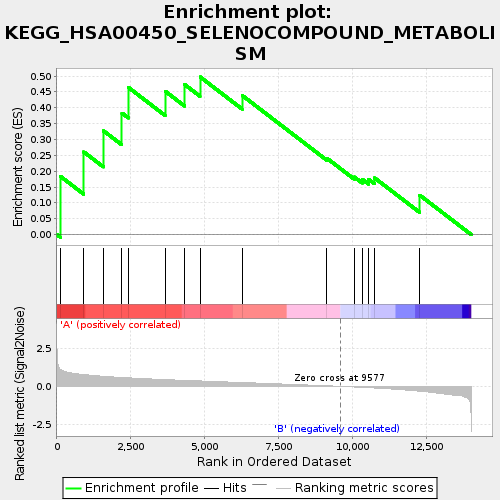

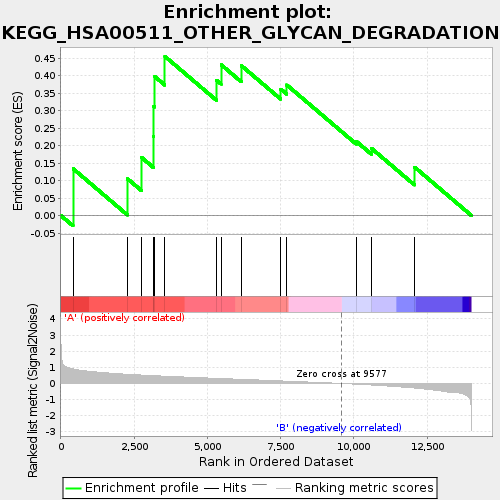

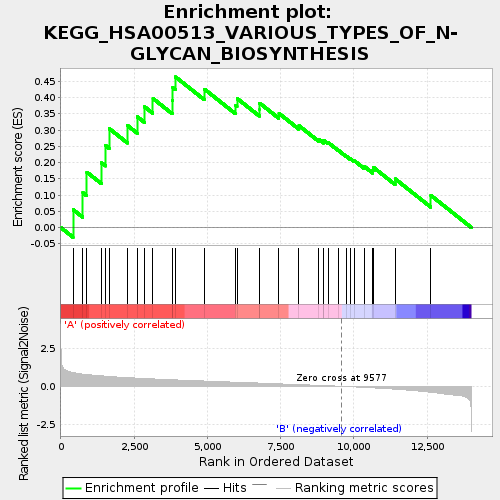

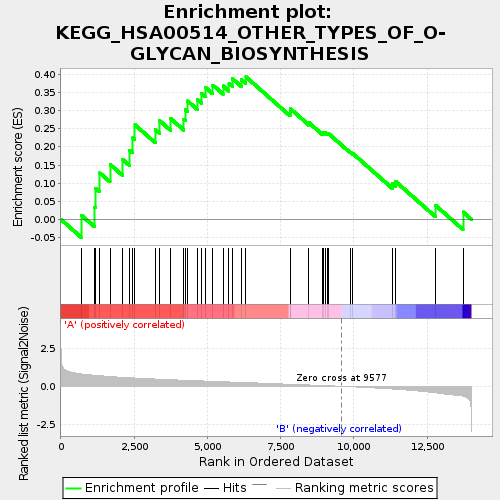

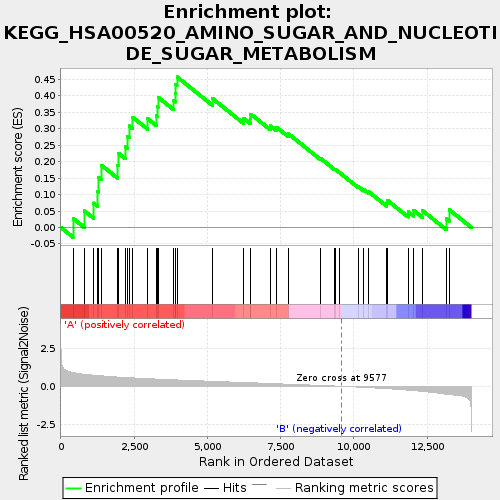

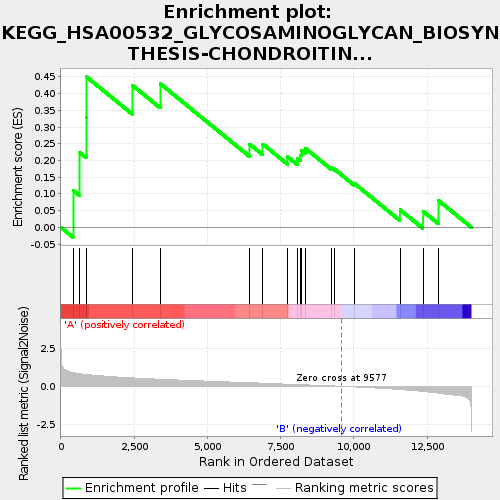

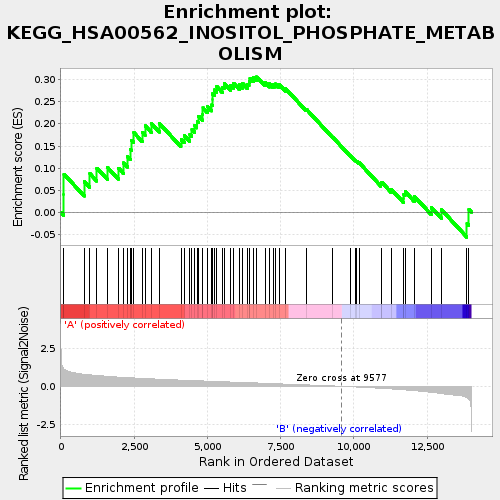

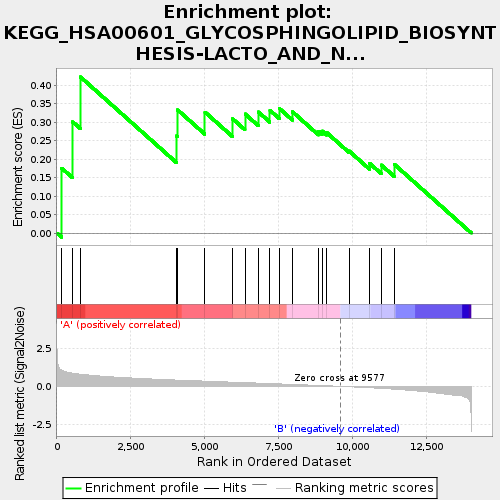

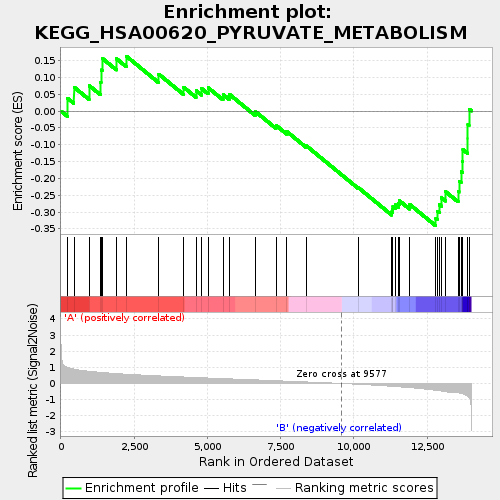

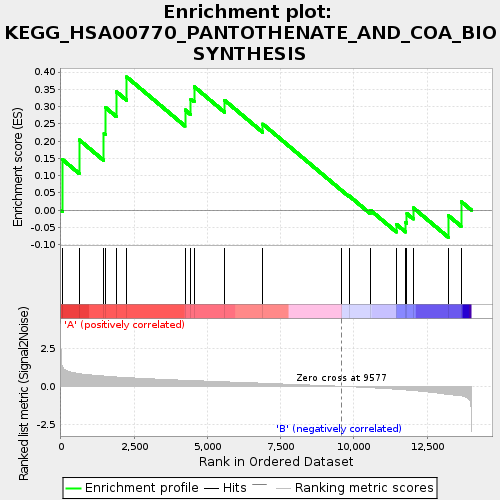

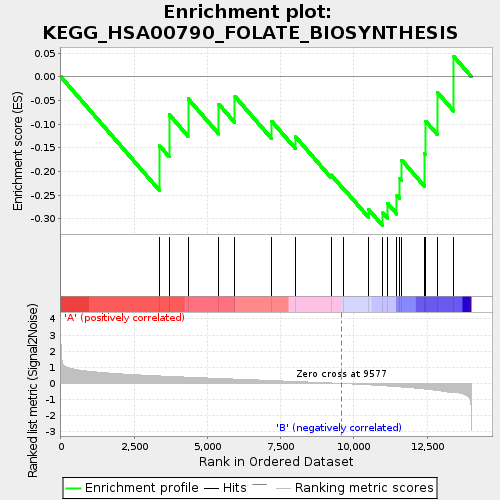

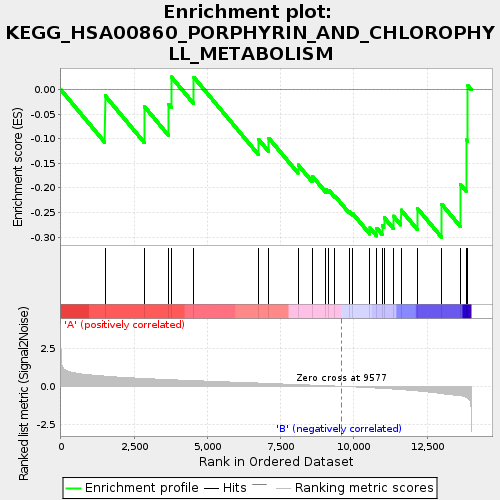

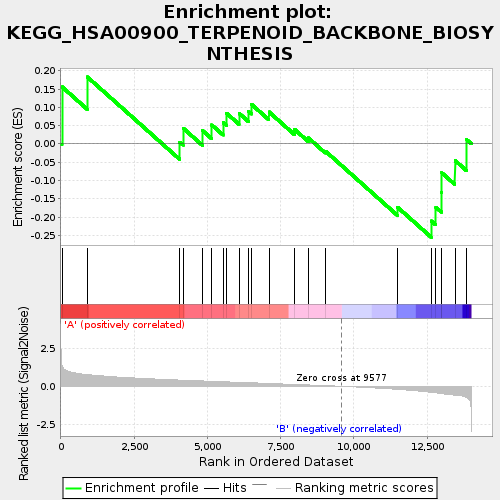

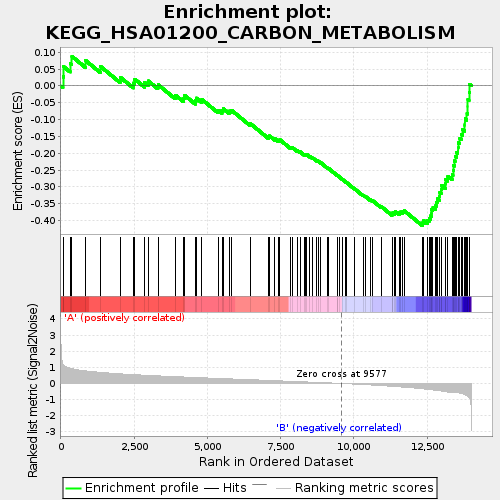

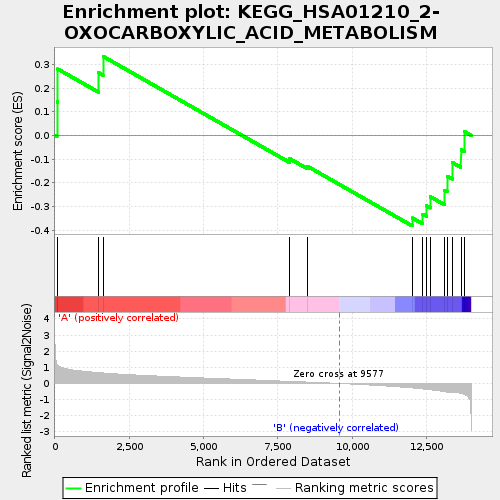

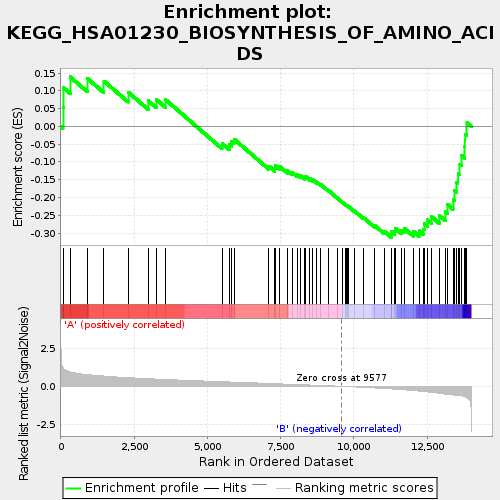

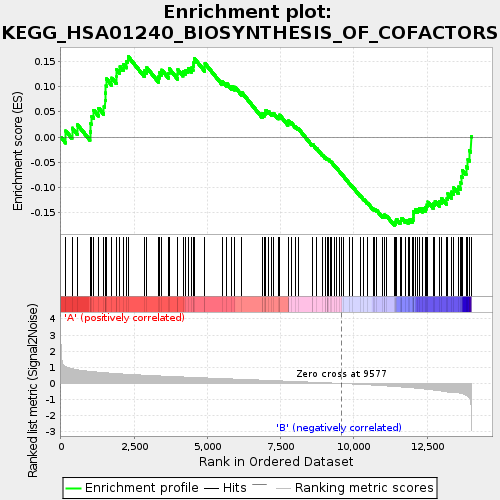

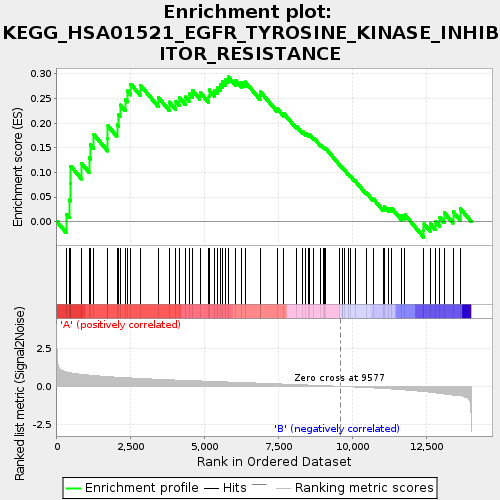

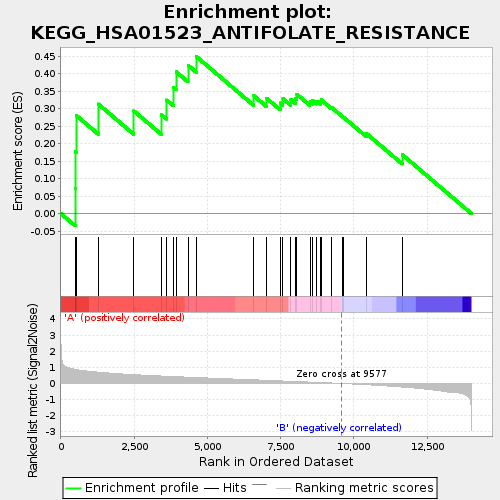

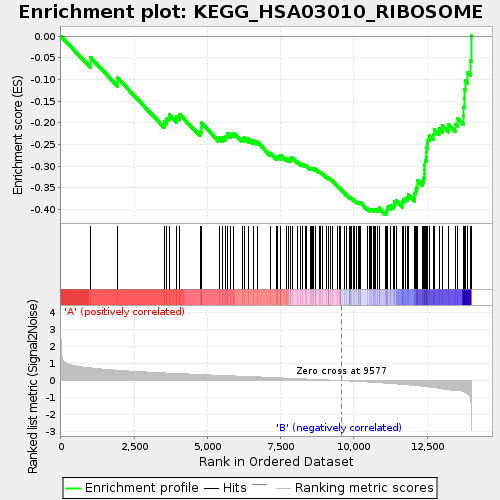

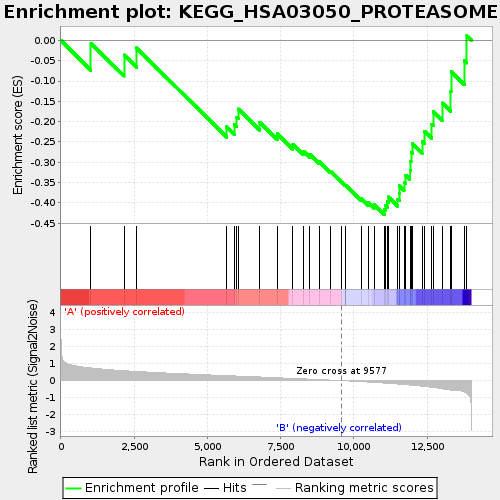

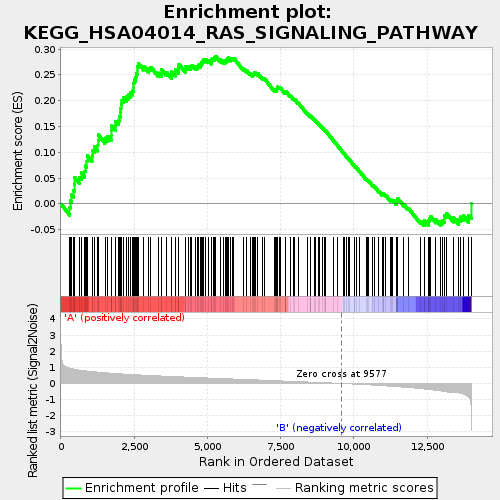

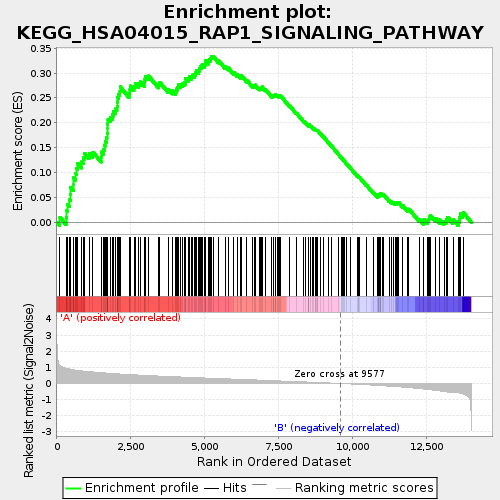

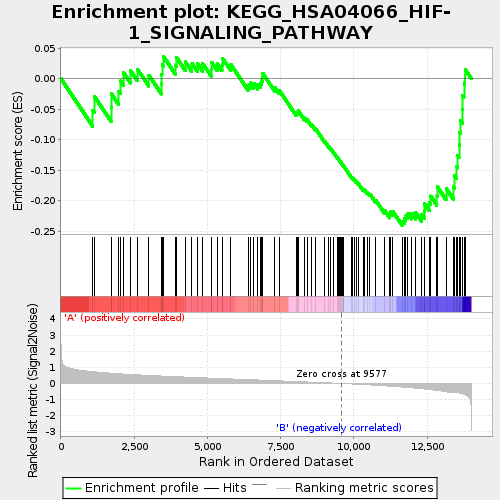

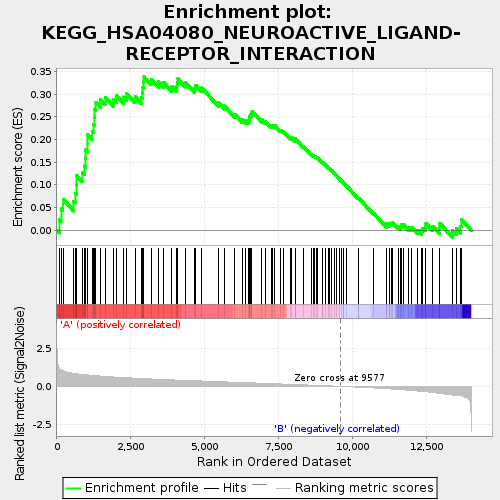

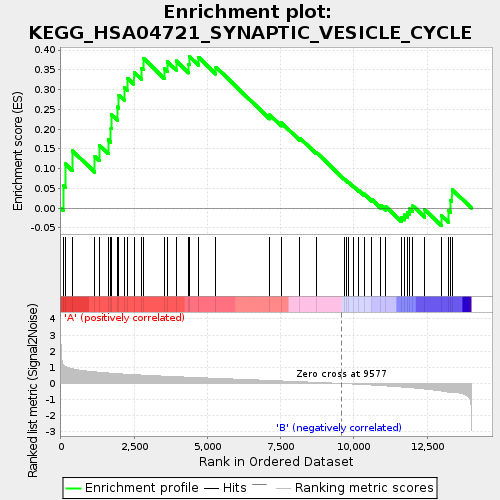

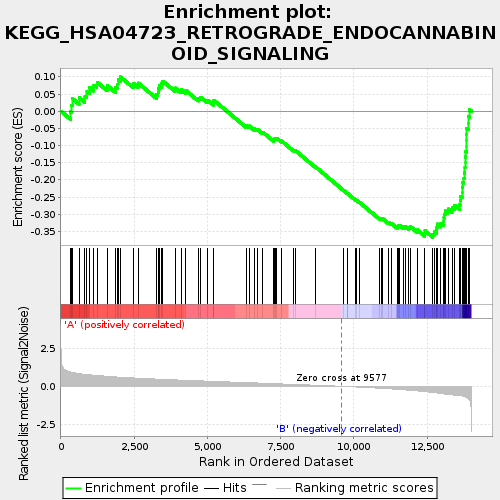

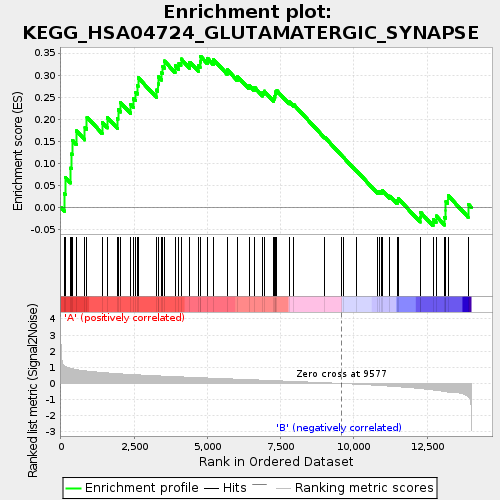

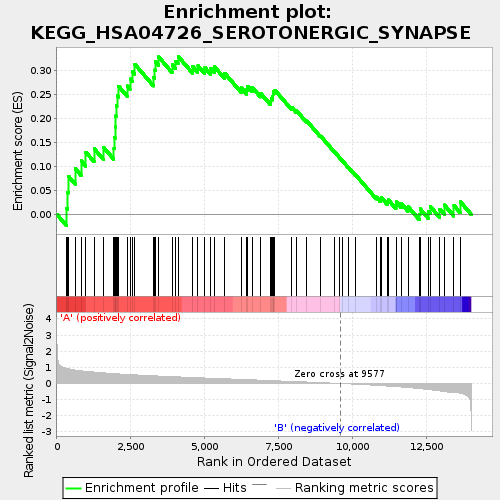

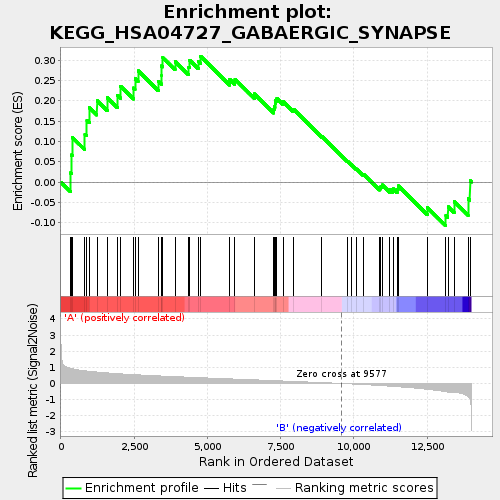

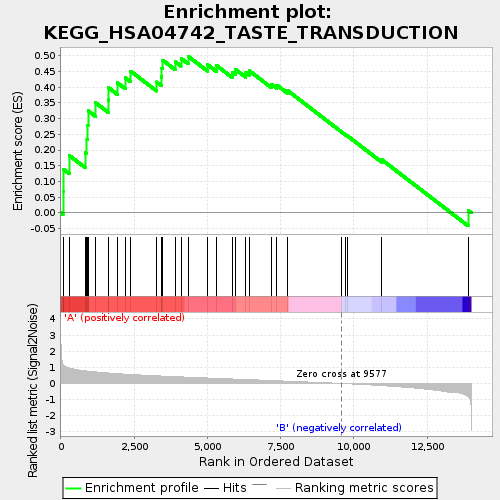

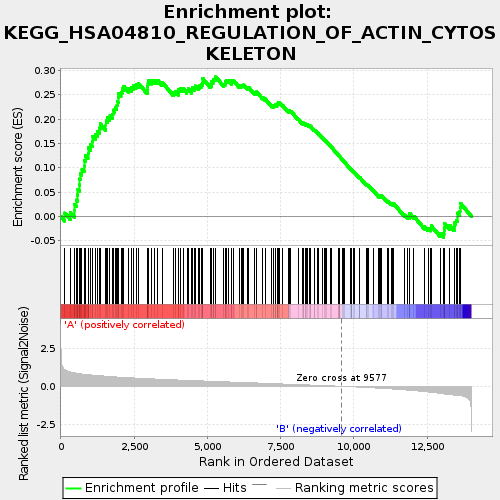

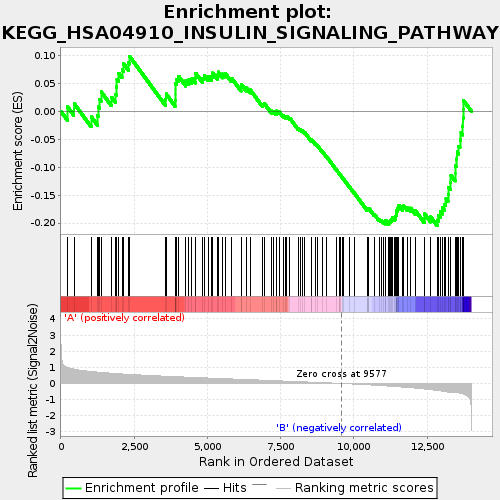

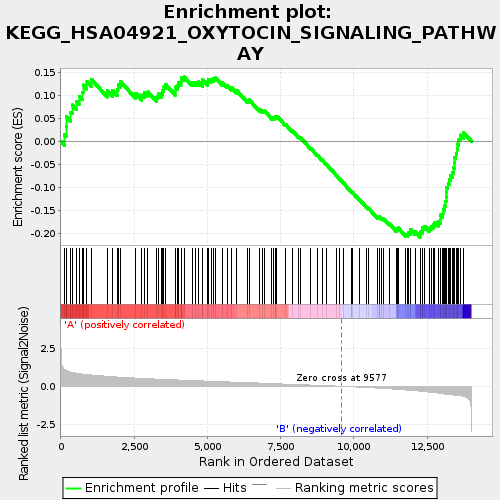

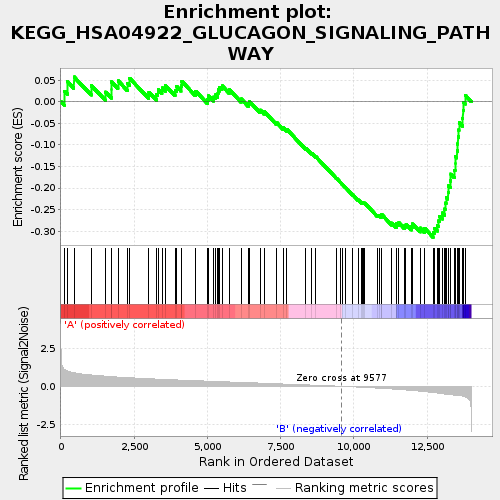

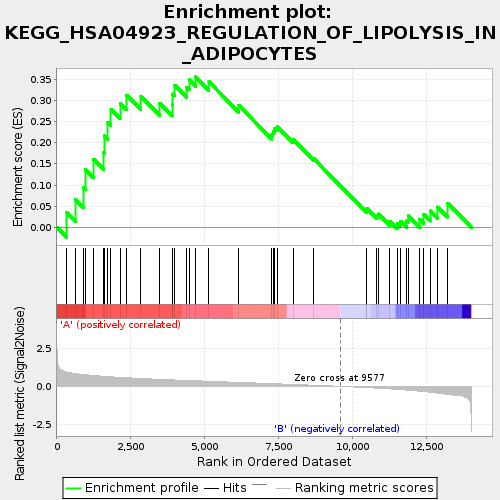

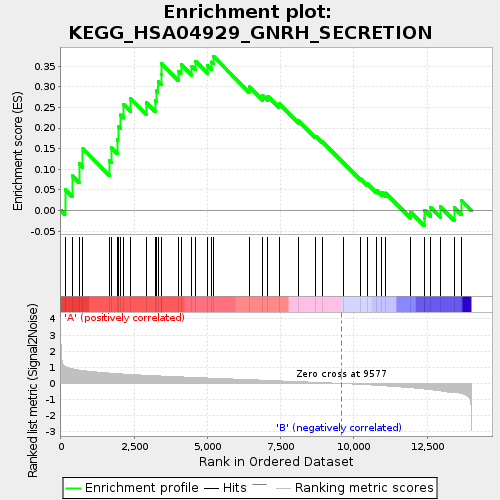

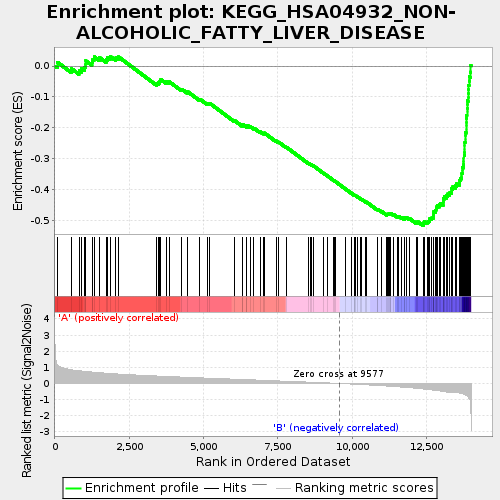

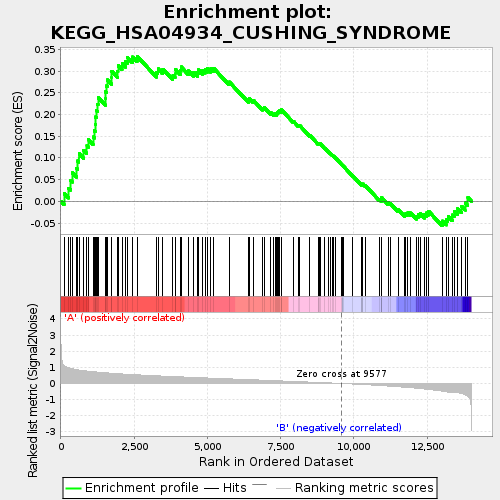

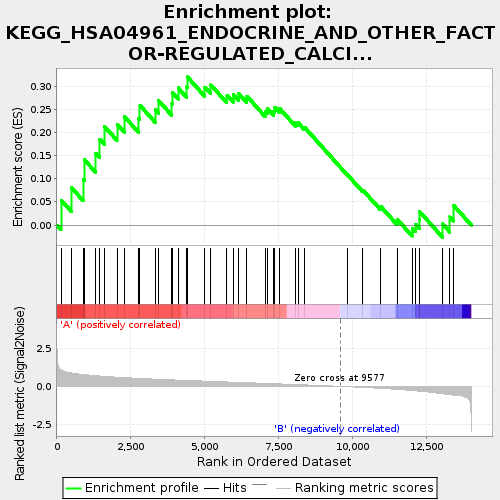

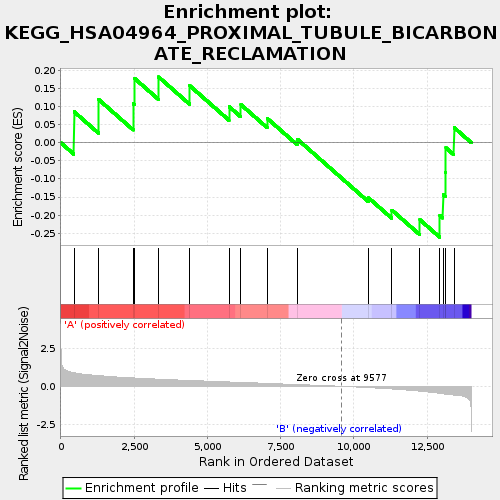

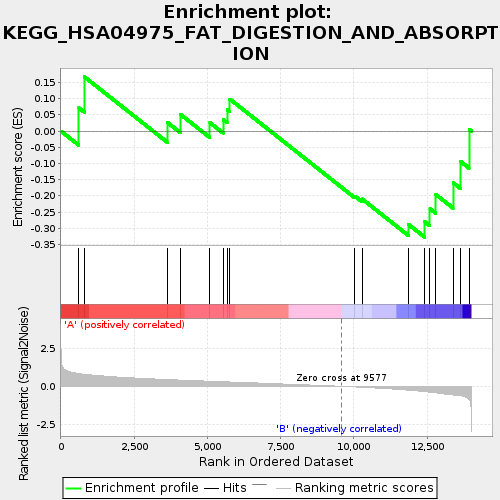

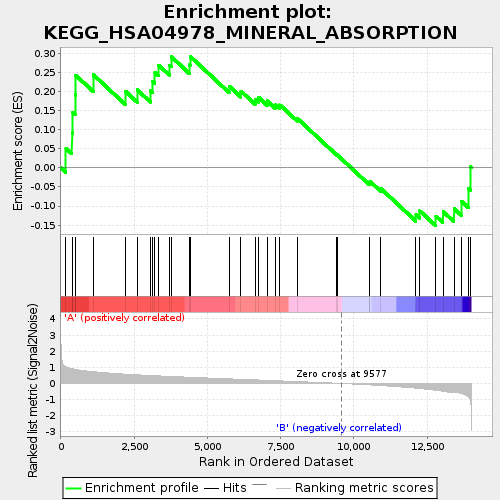

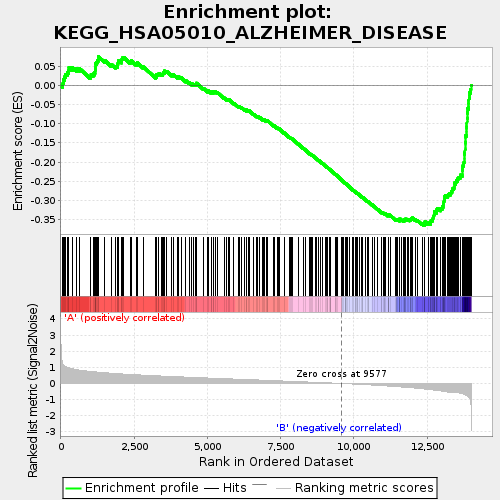

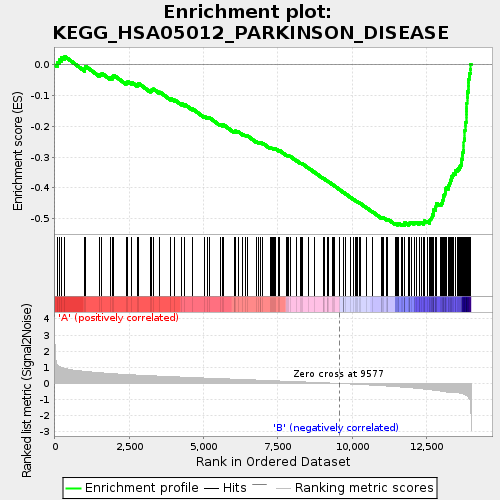

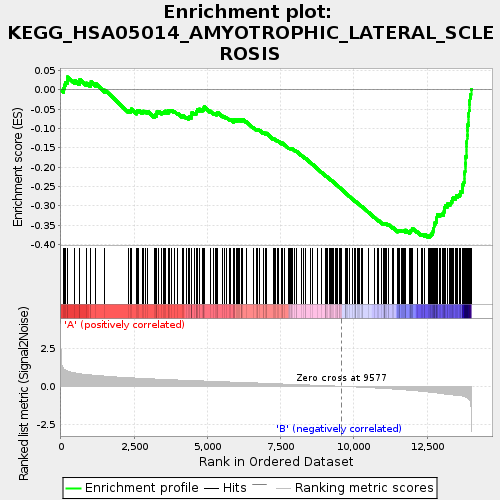

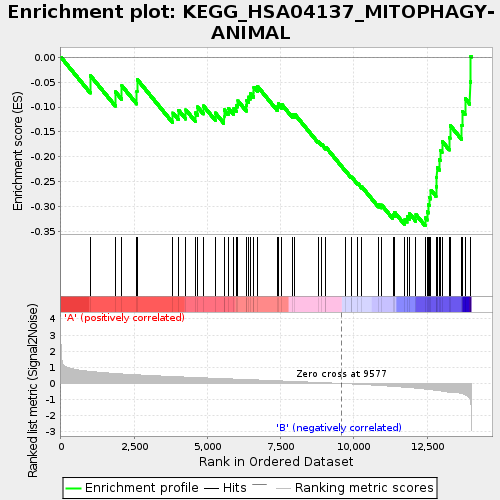

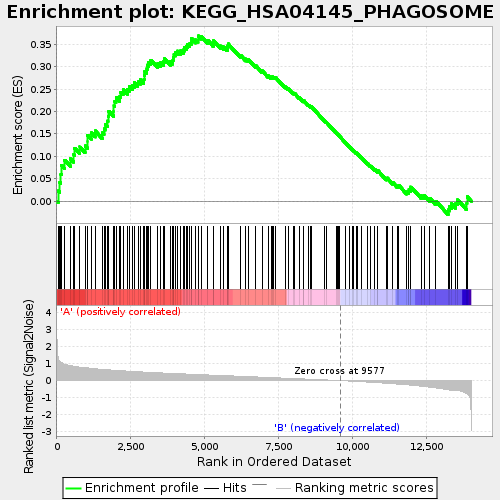

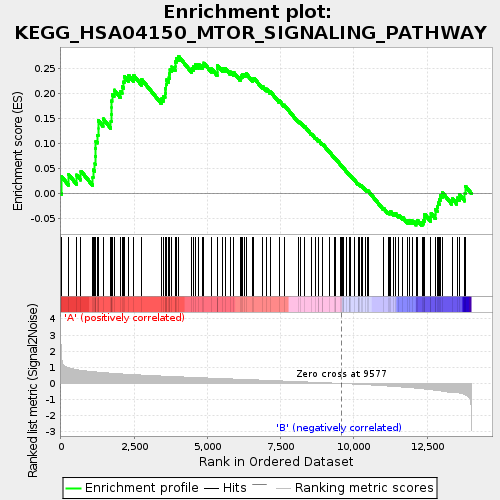

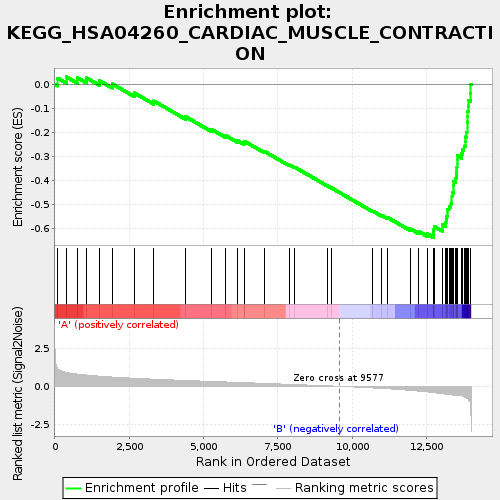

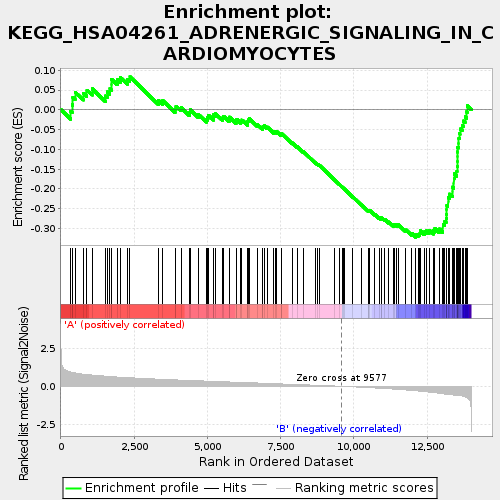

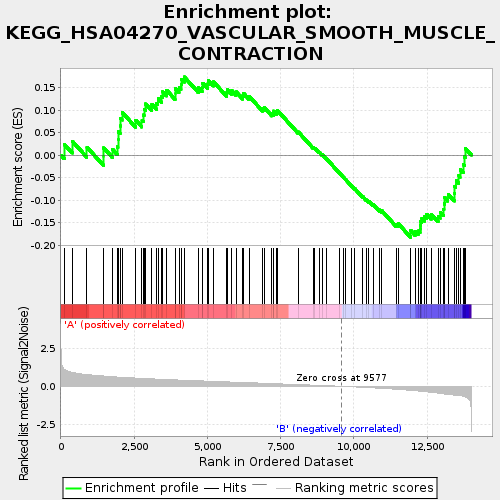

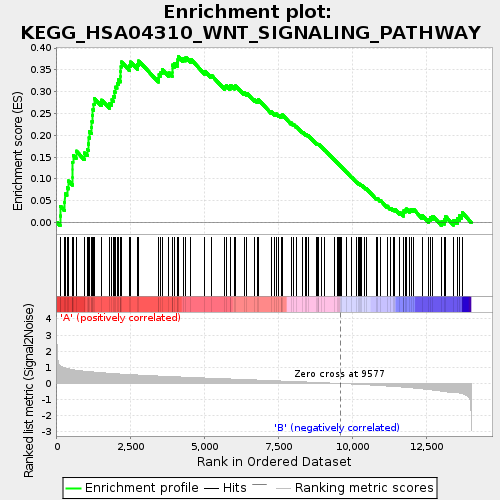

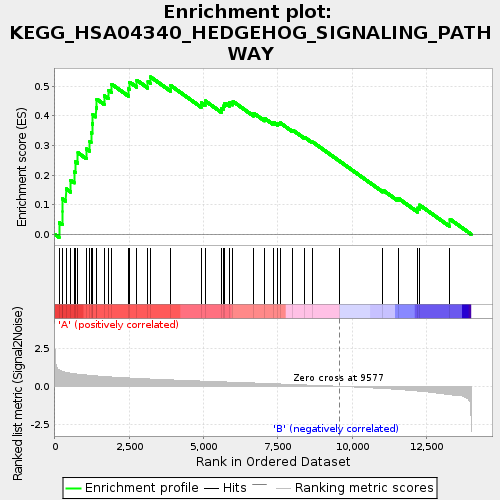

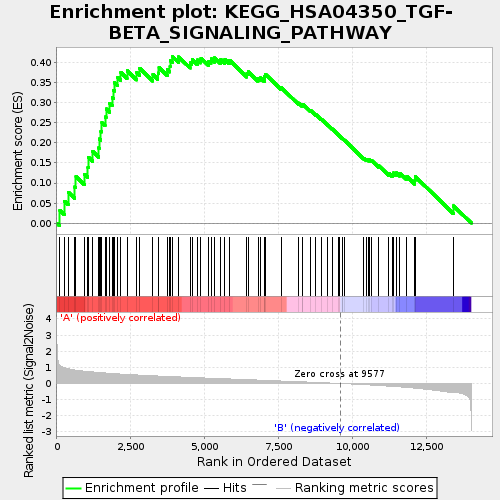

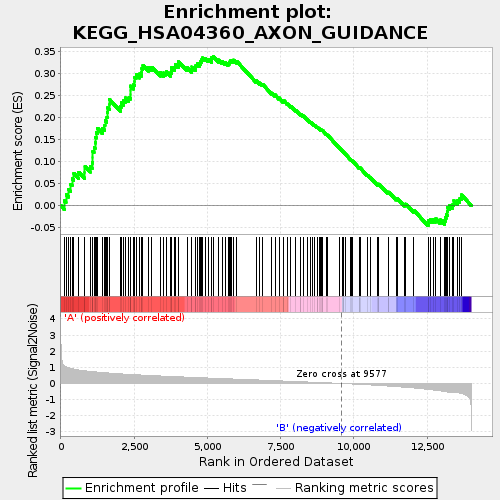

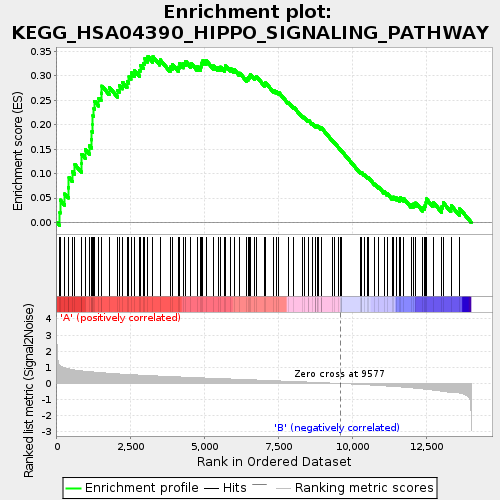

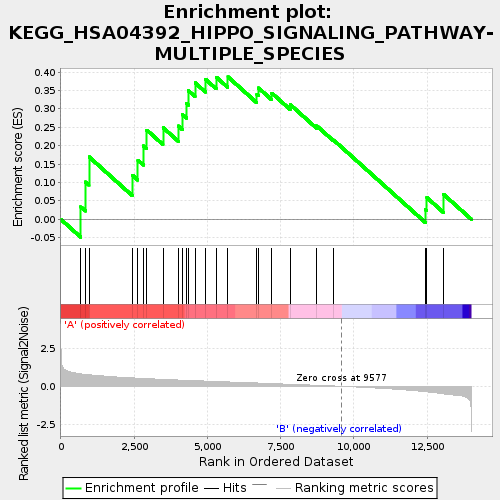

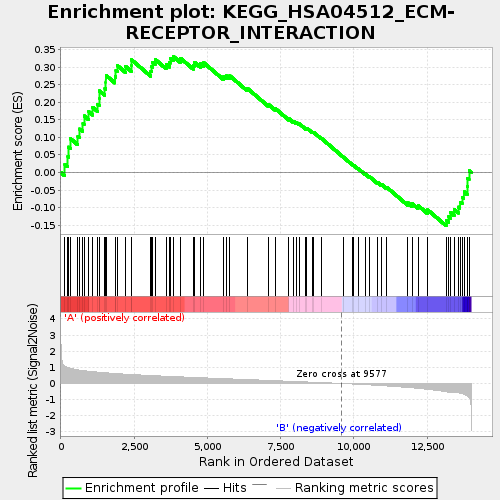

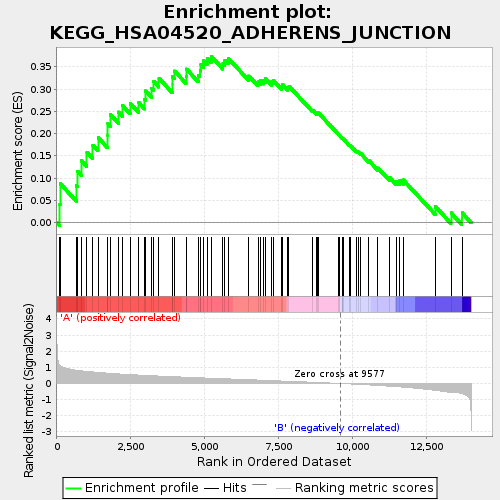

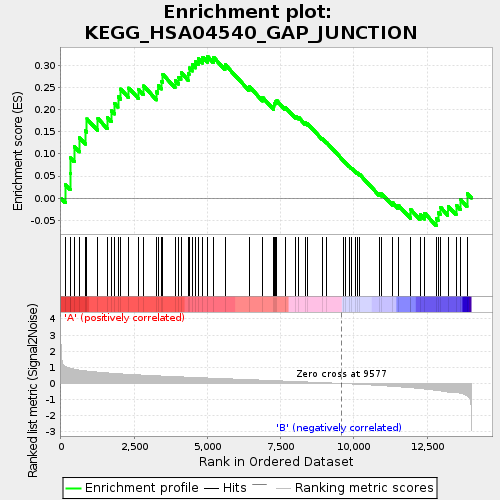

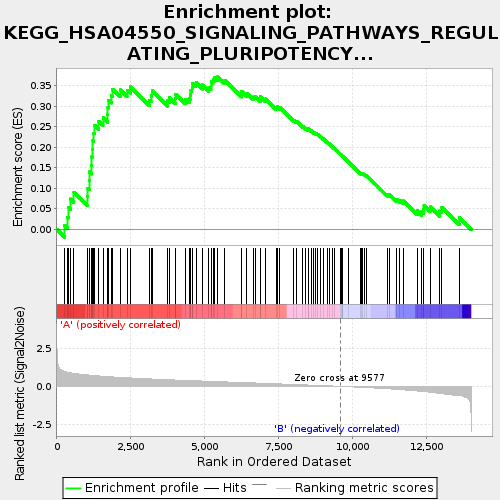

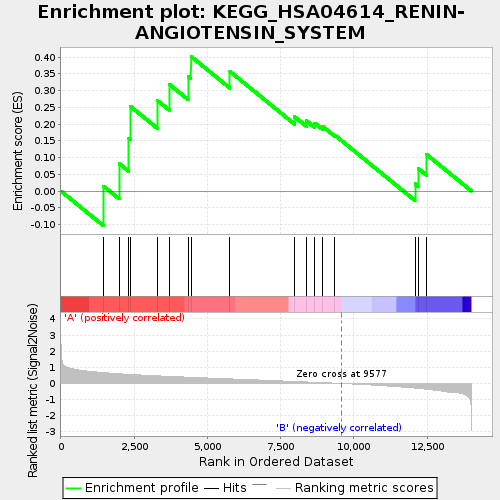

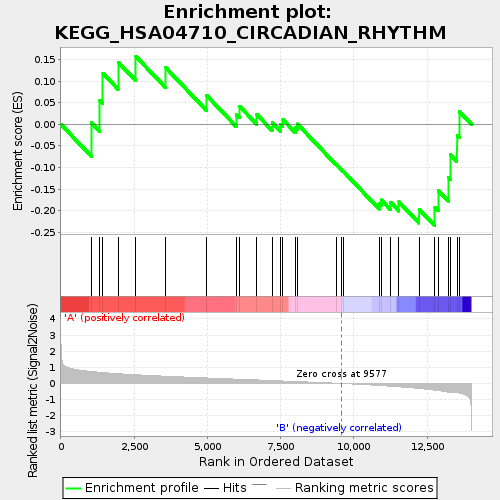

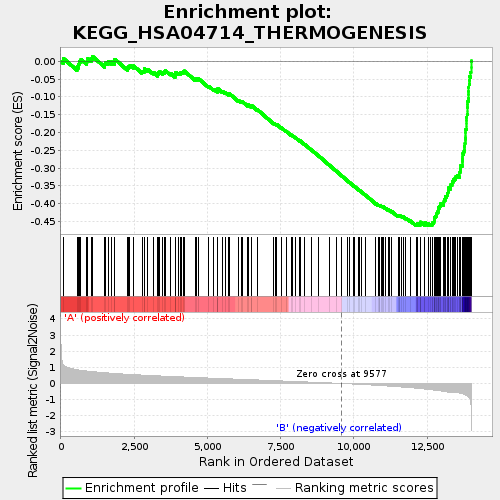

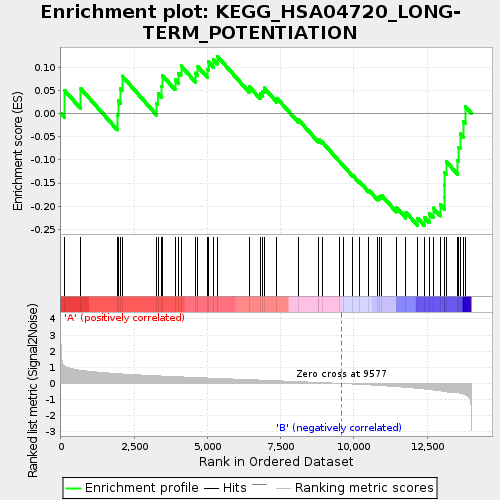

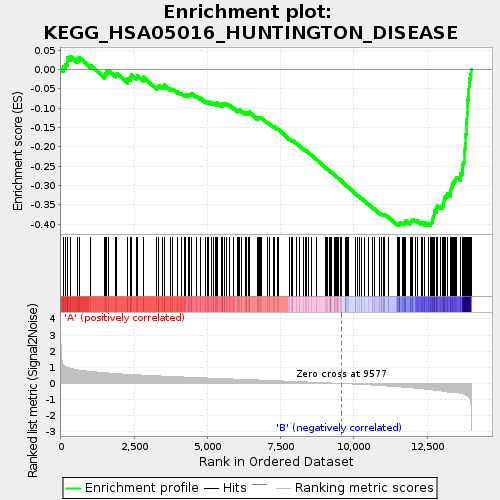

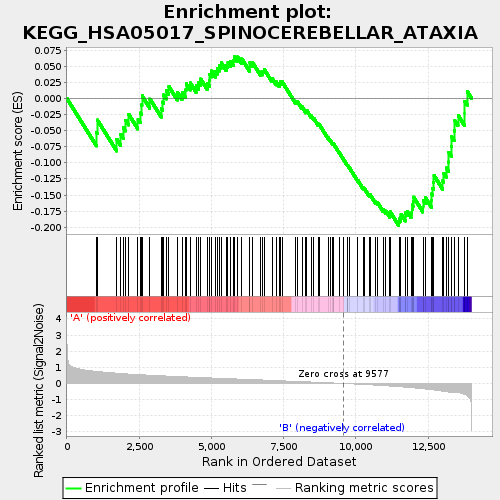

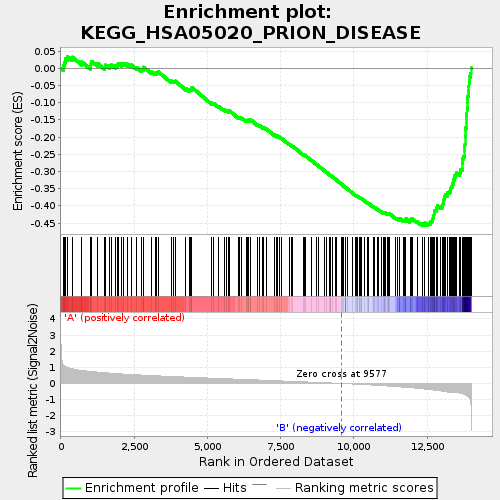

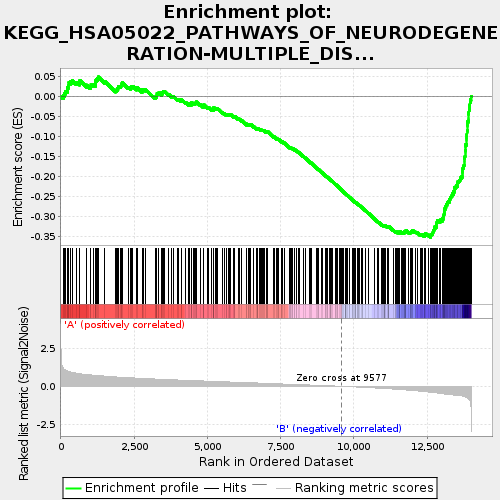

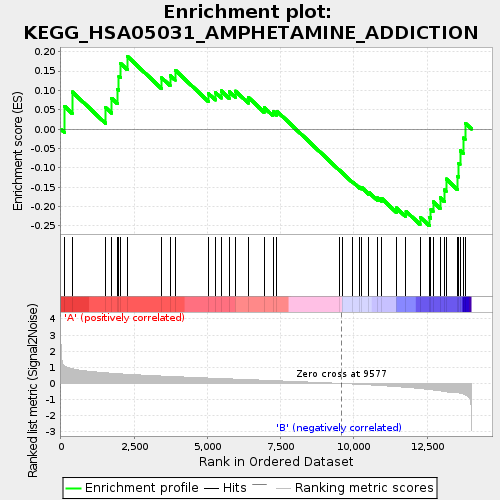

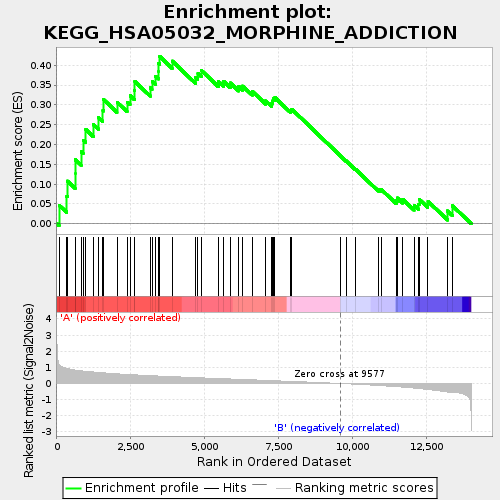

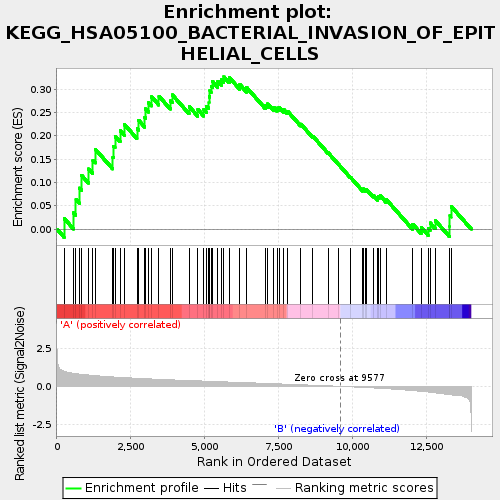

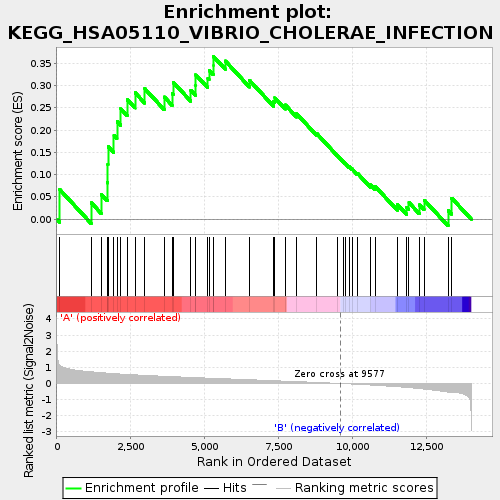

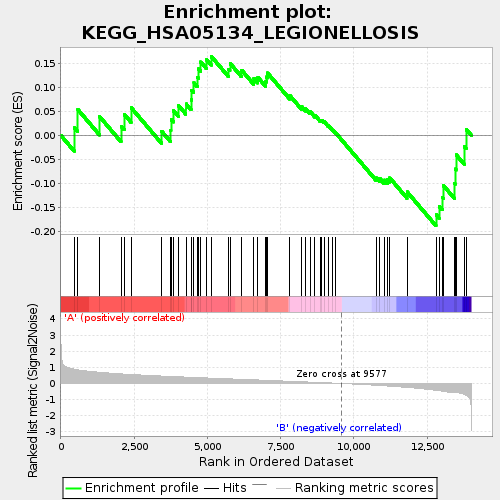

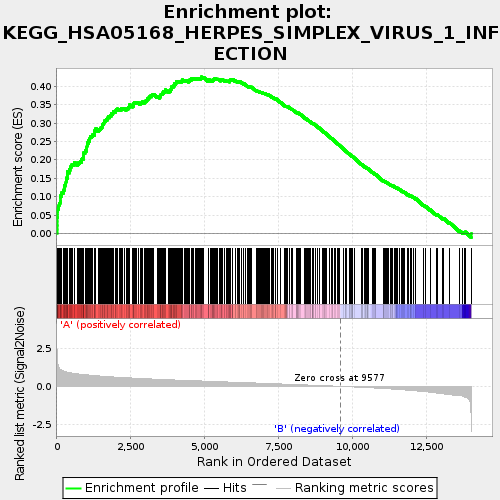

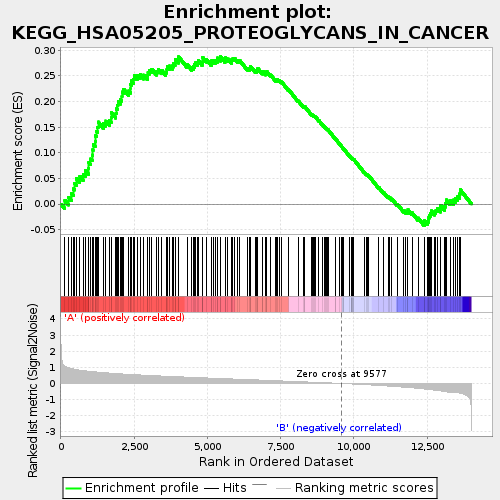

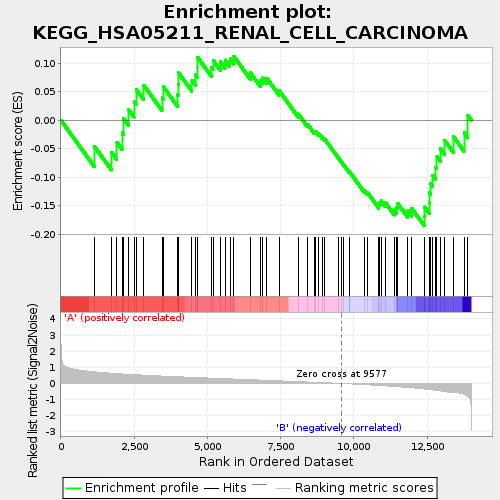

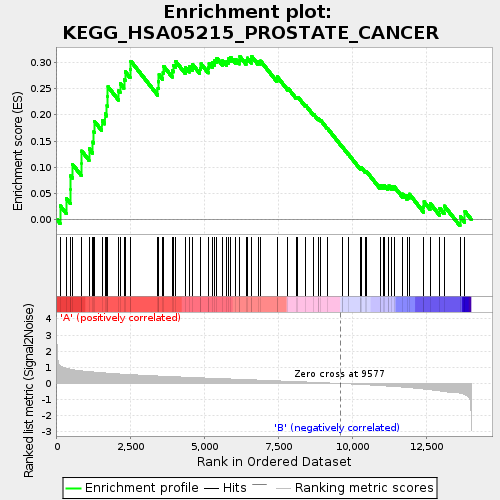

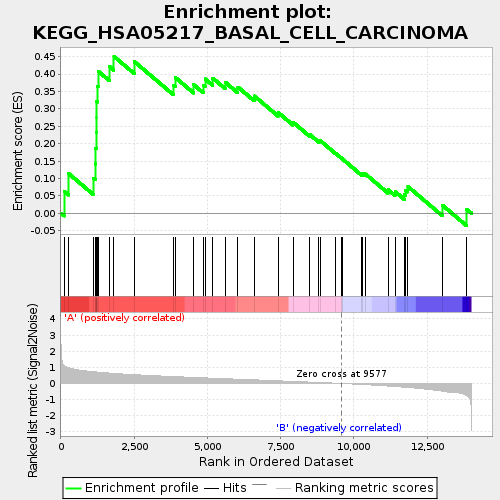

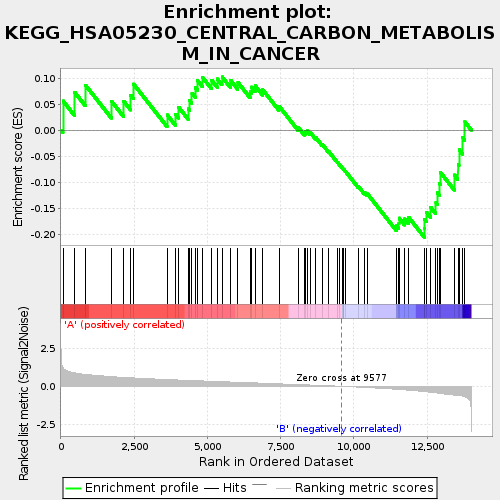

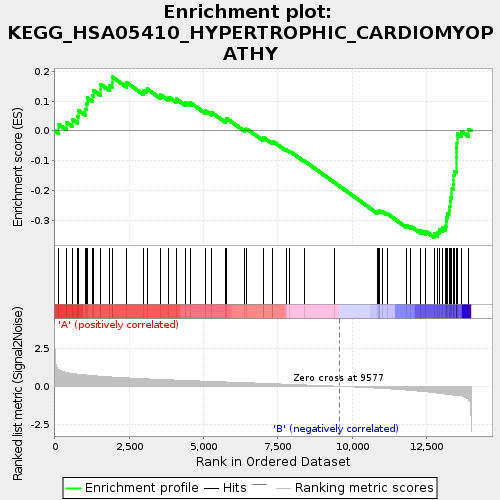

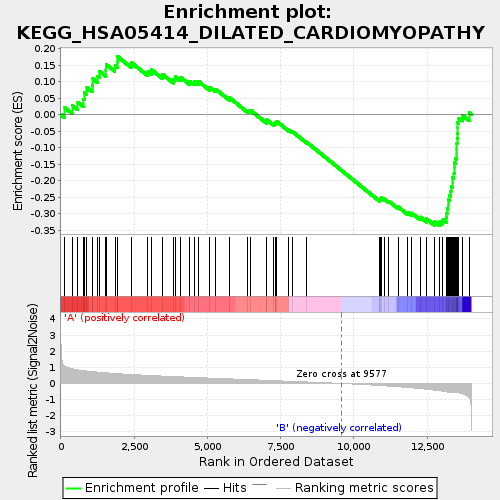

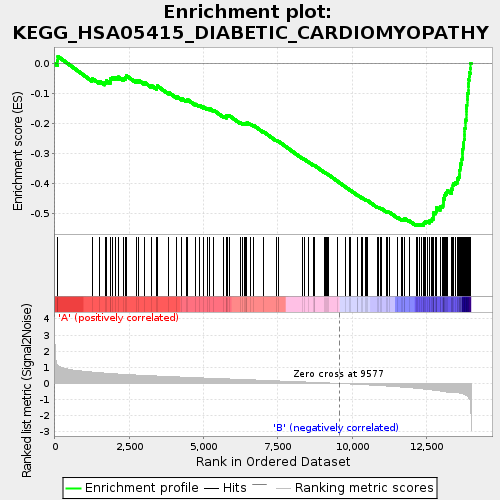

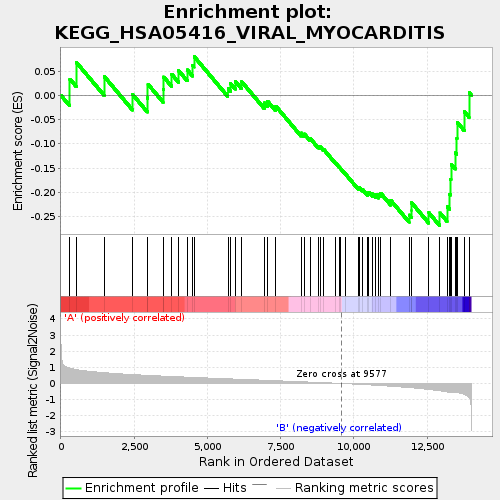

Supplement: Supplementary 5 — Gene set enrichment analysis of IN vs. CO. [file 1154808.f5.docx]

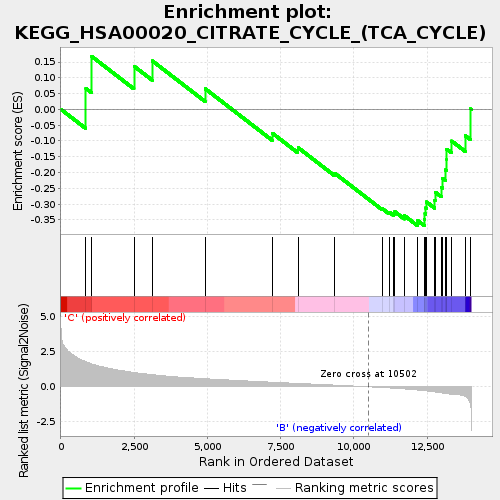

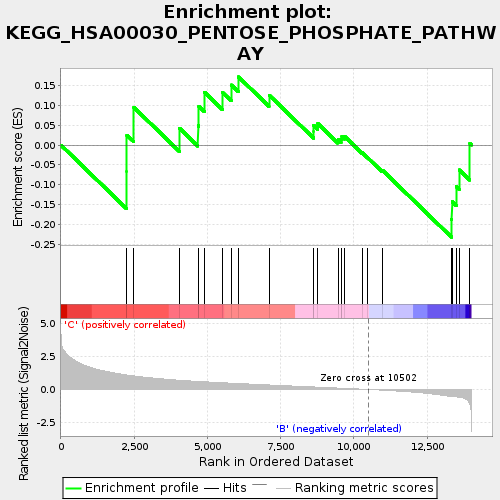

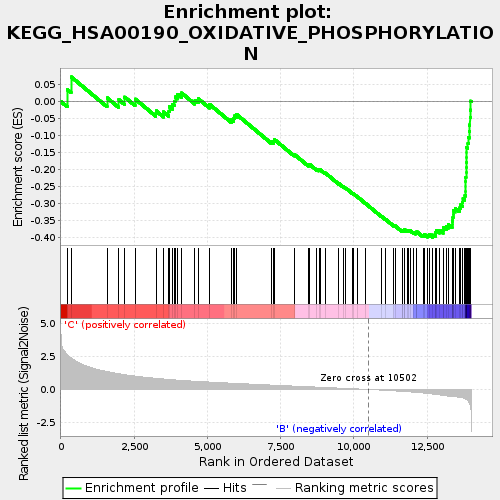

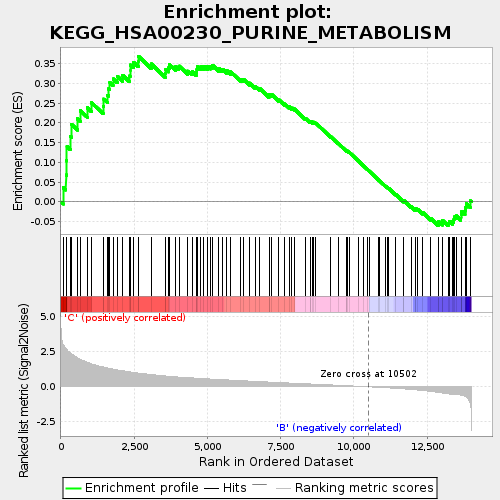

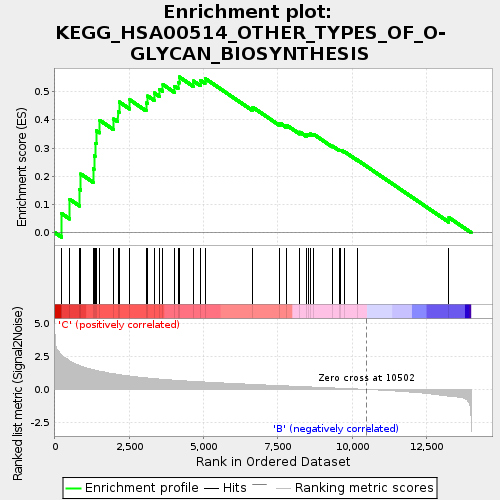

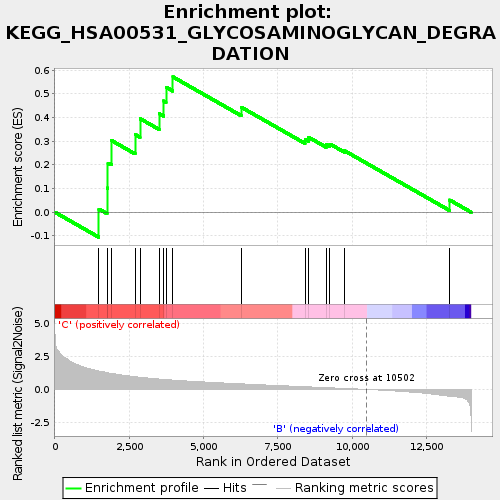

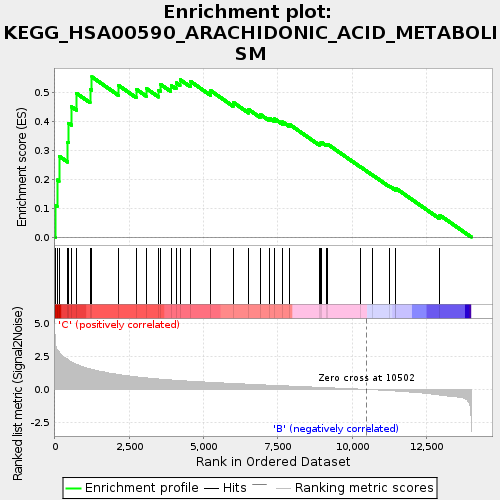

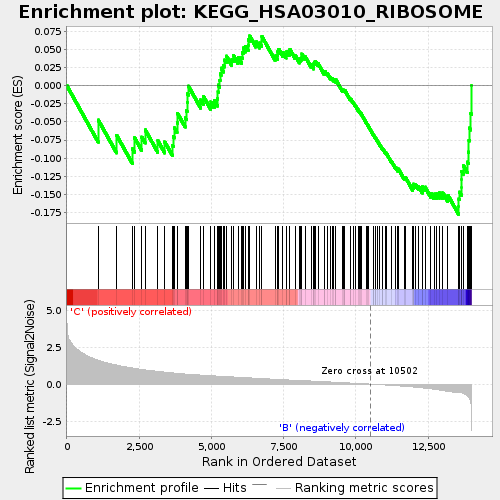

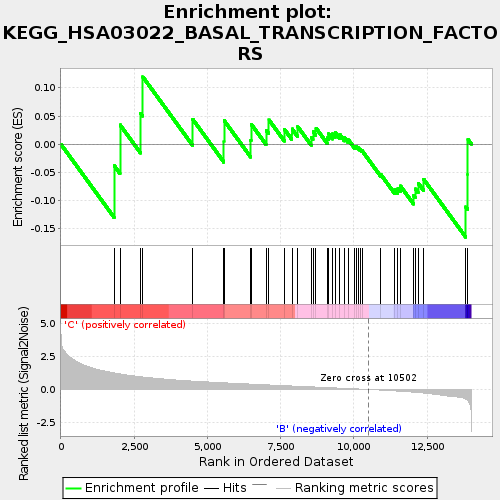

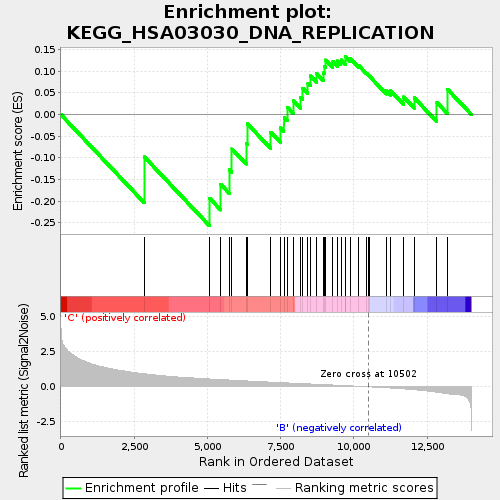

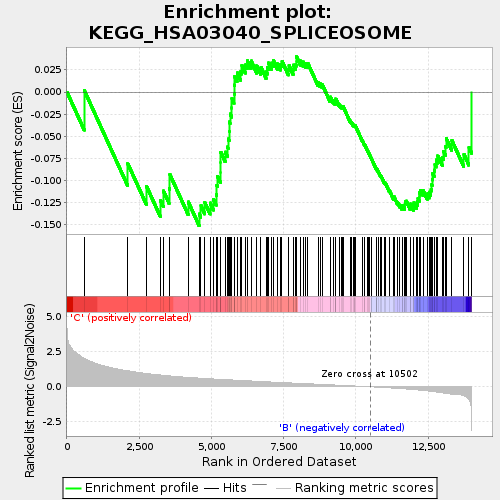

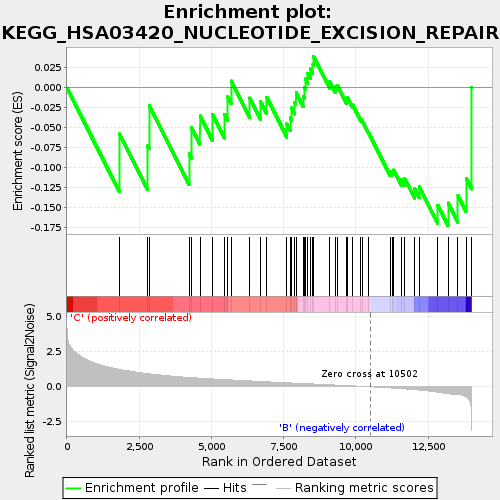

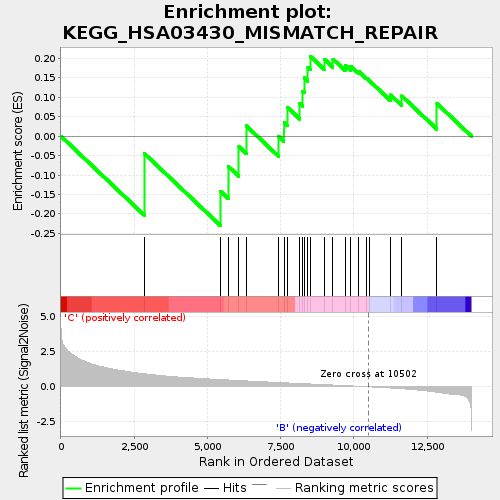

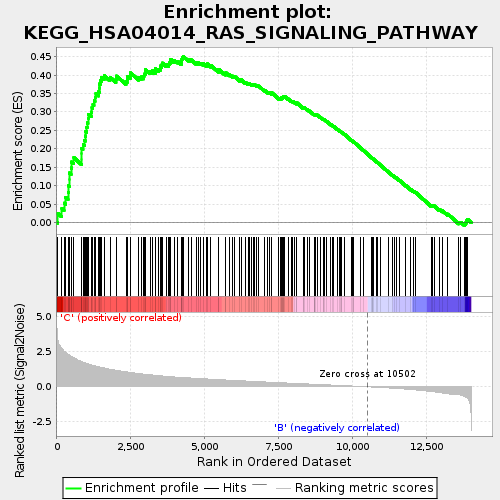

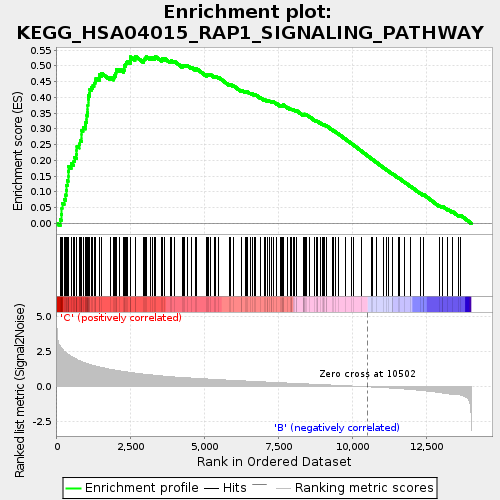

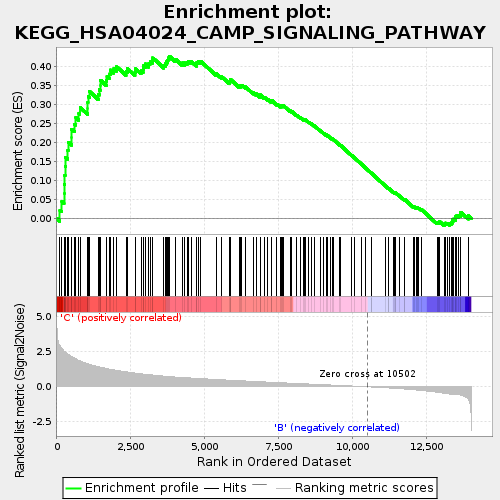

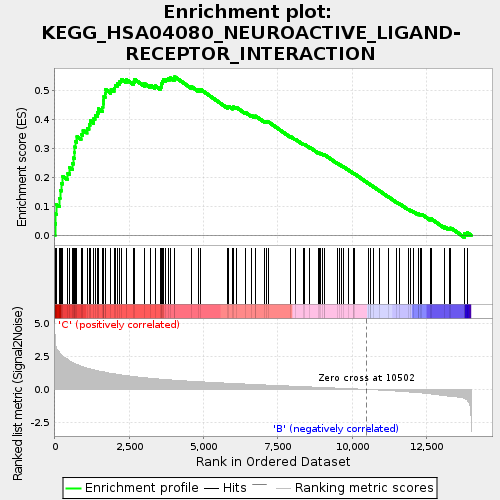

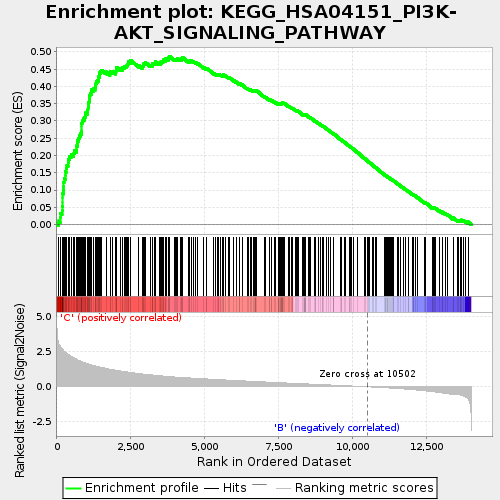

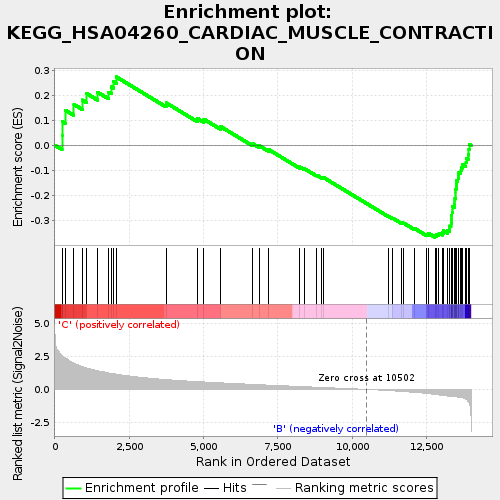

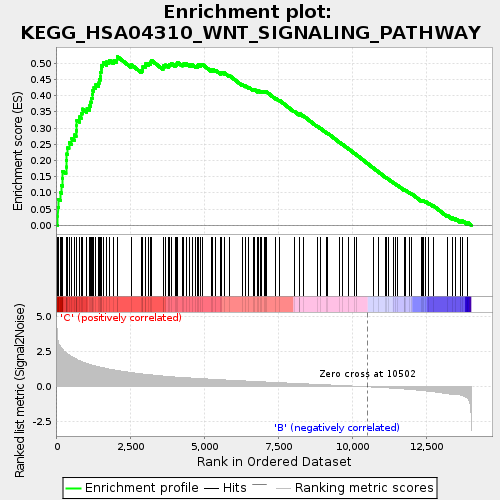

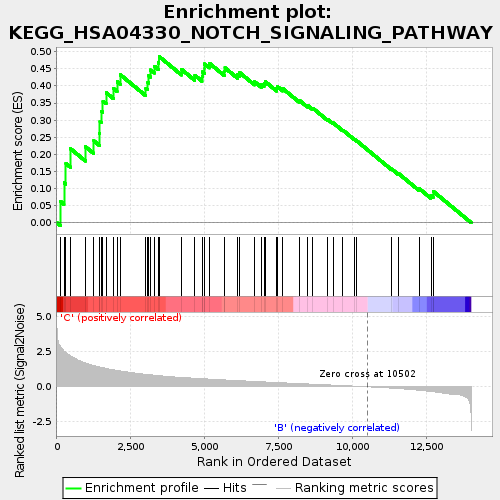

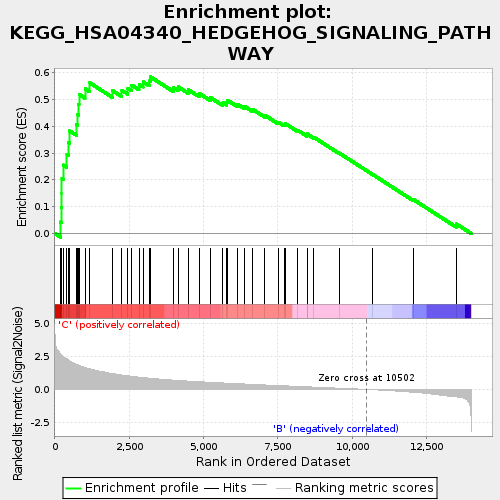

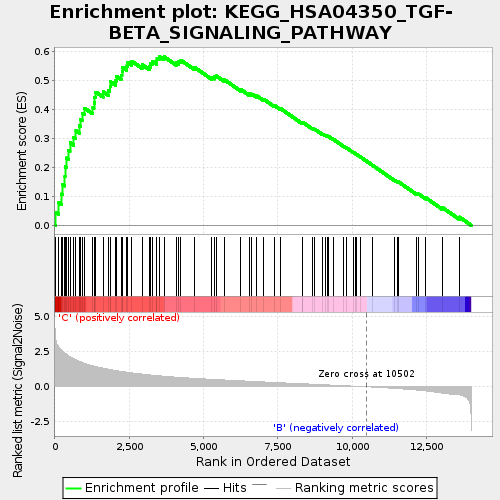

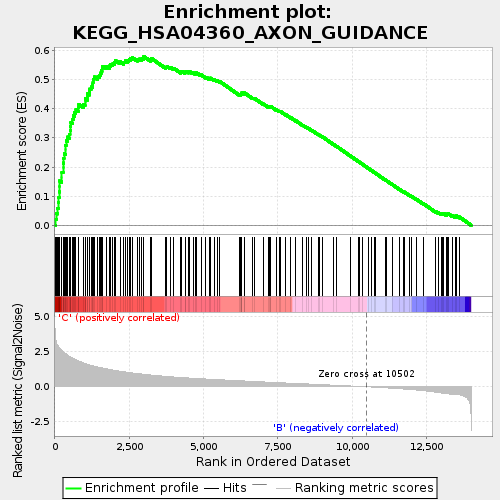

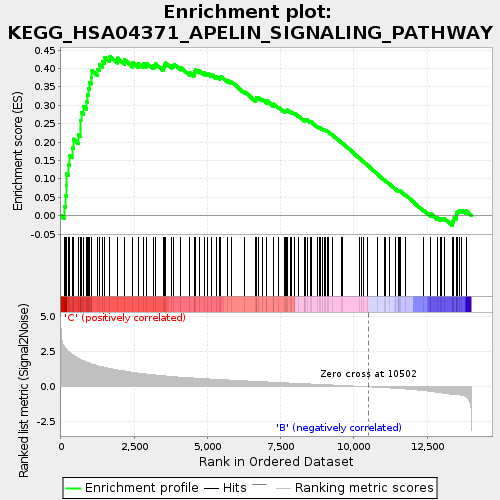

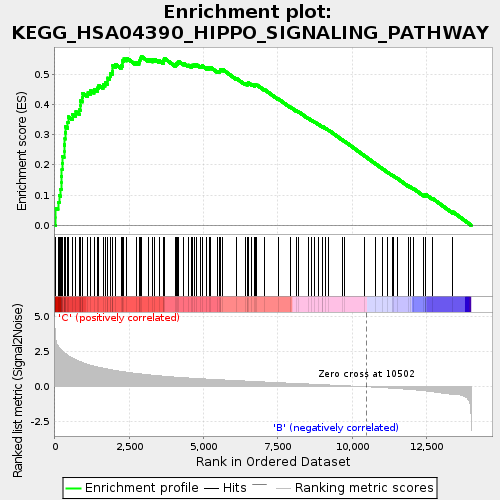

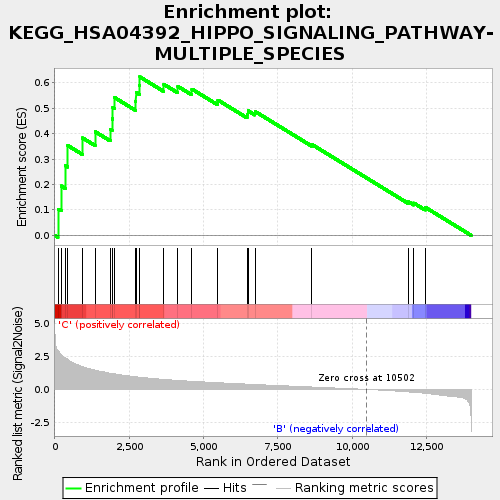

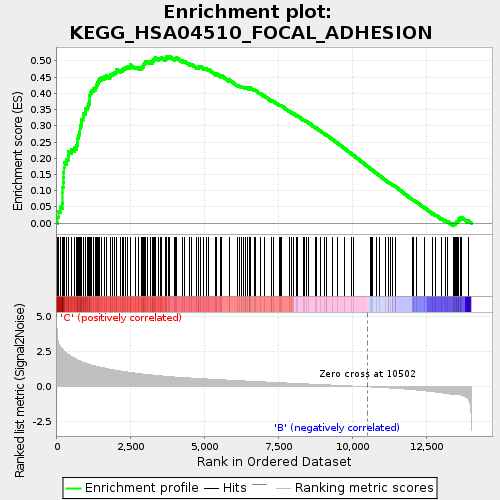

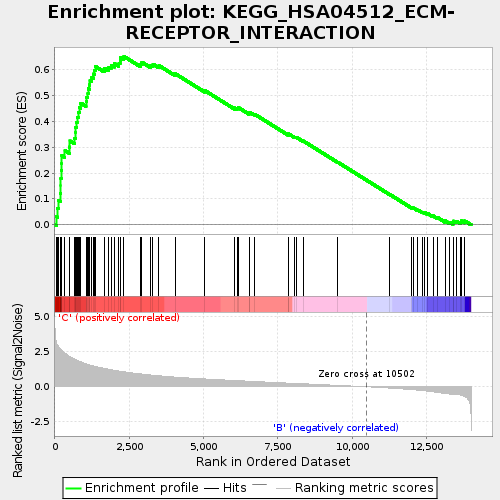

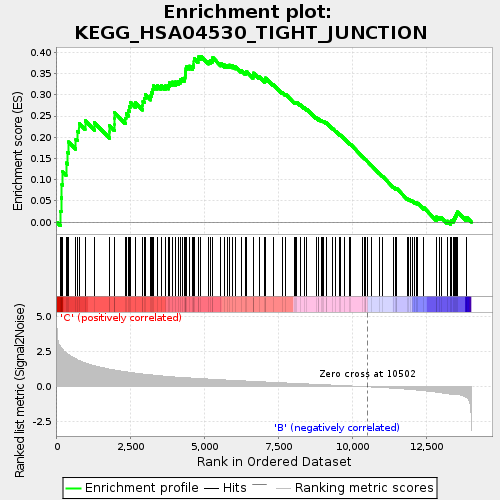

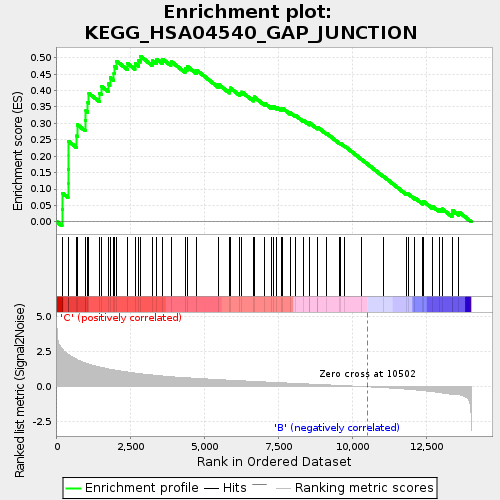

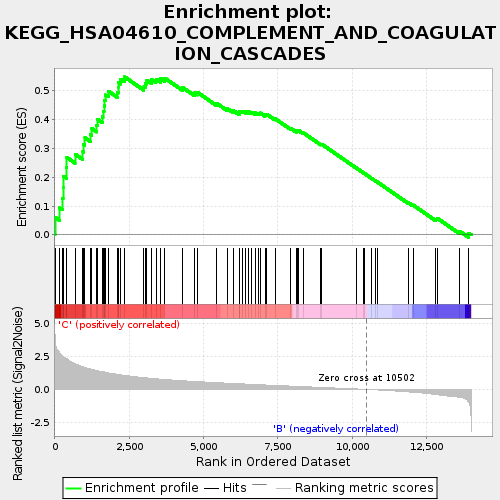

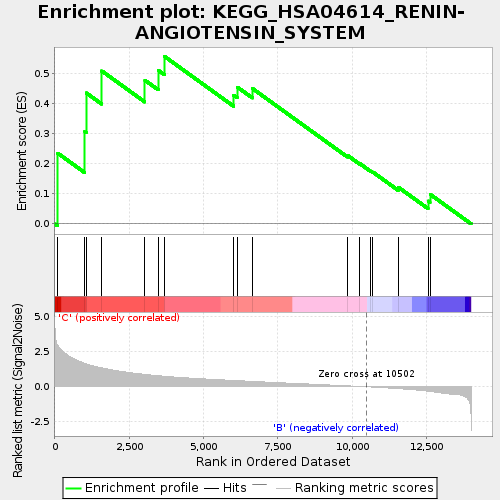

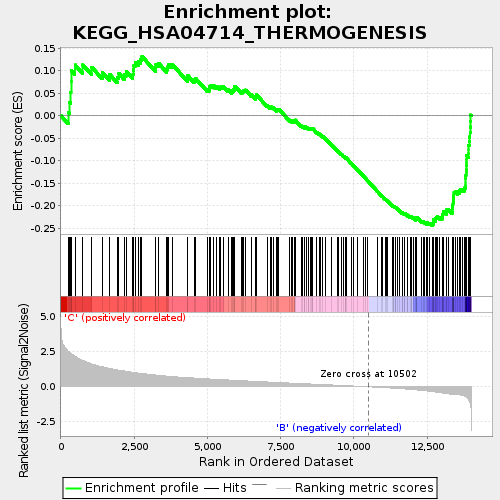

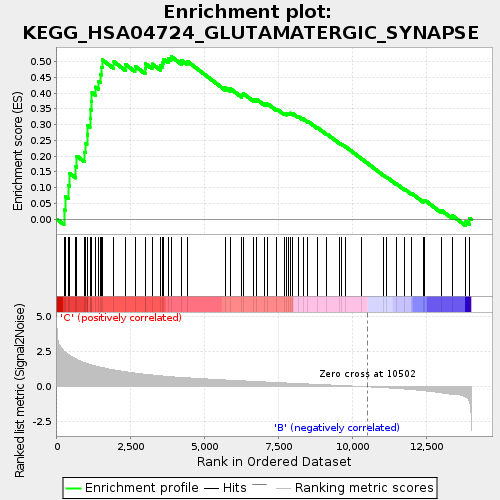

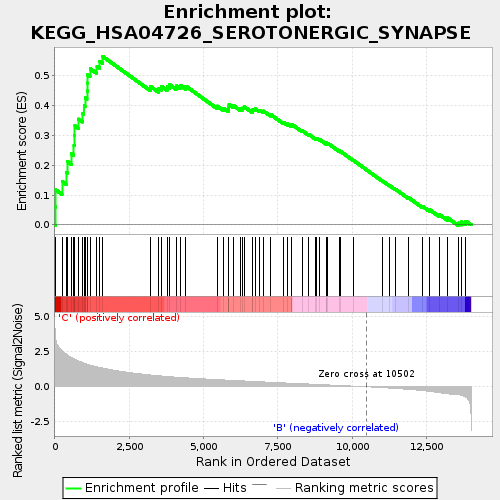

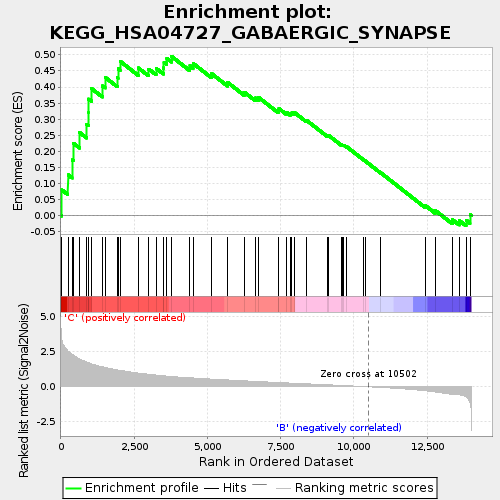

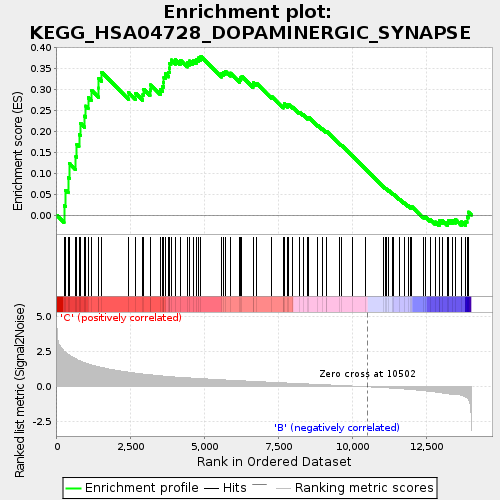

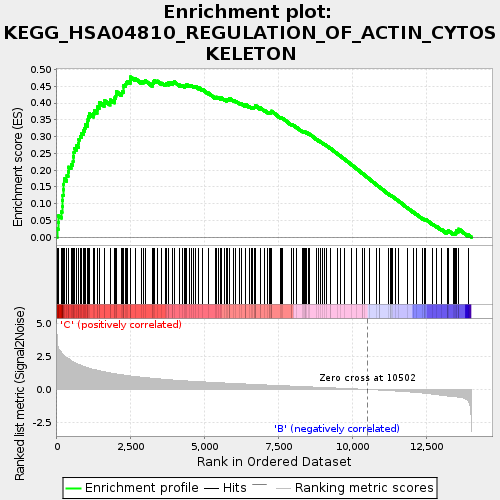

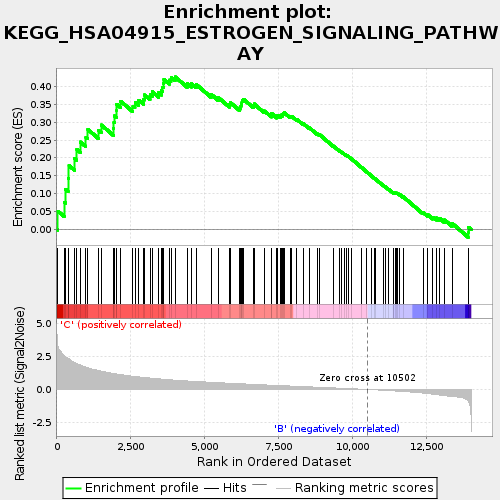

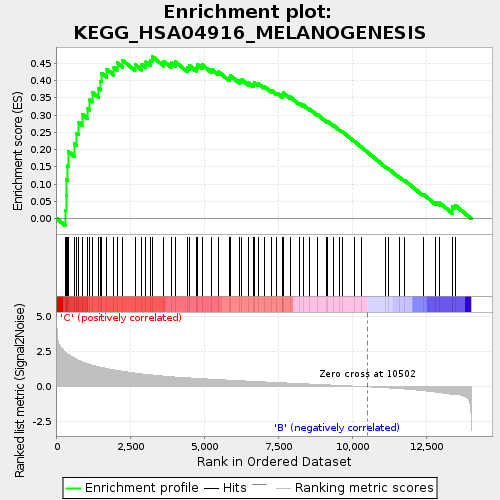

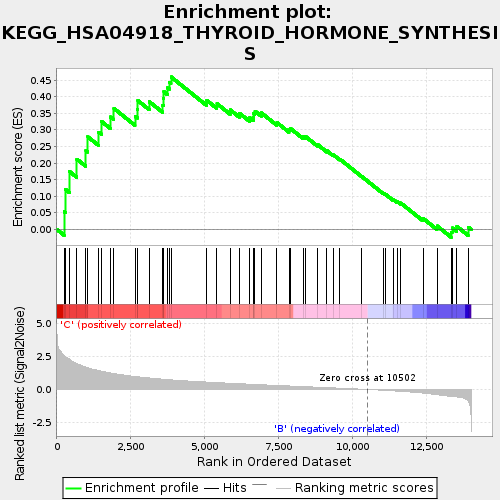

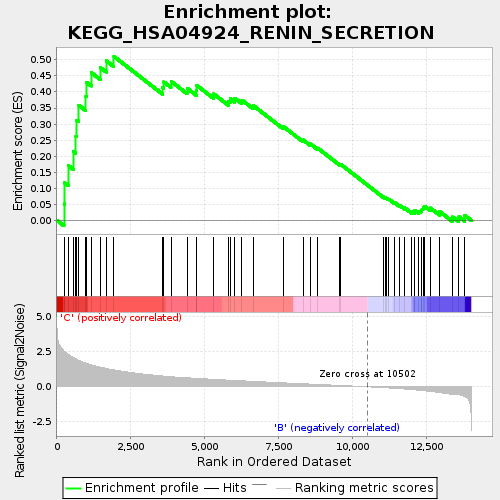

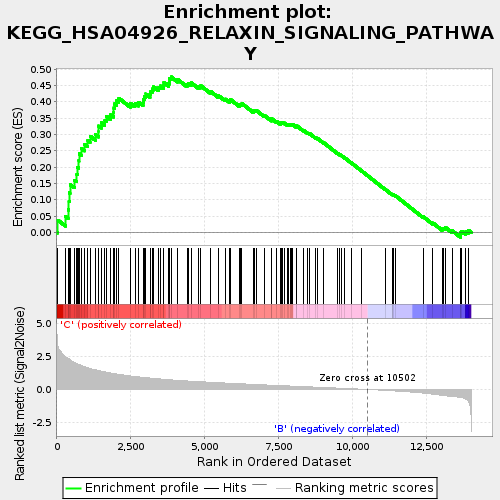

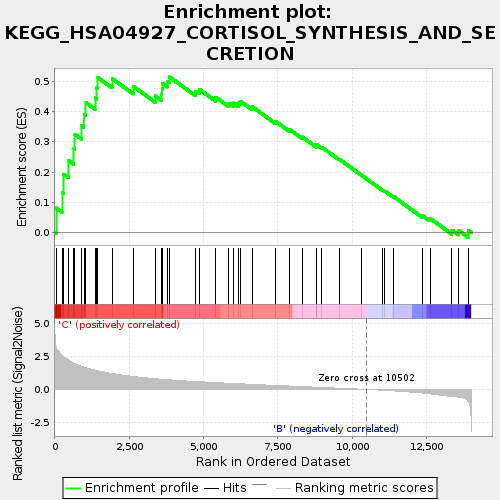

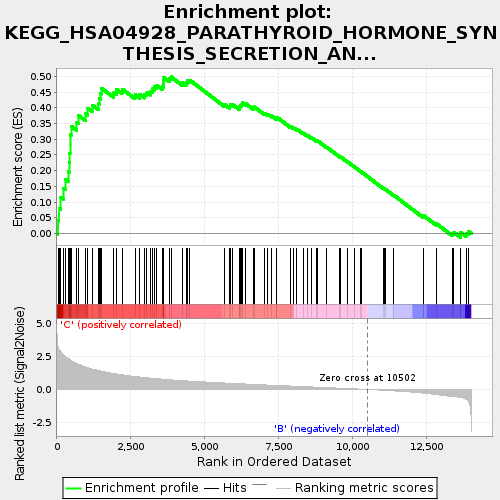

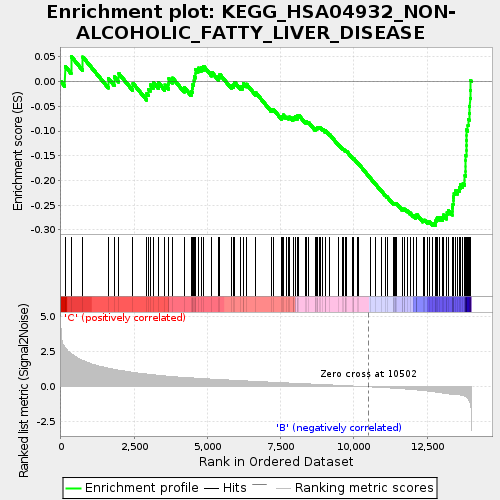

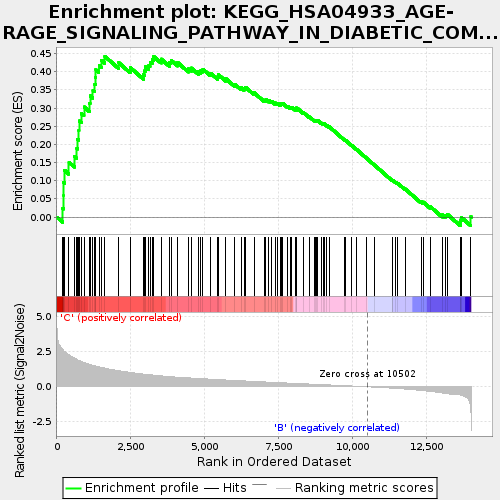

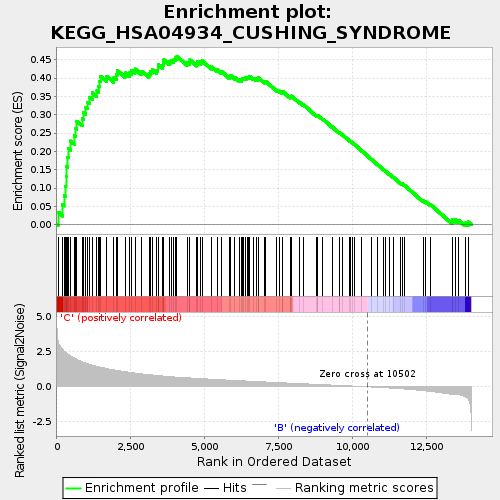

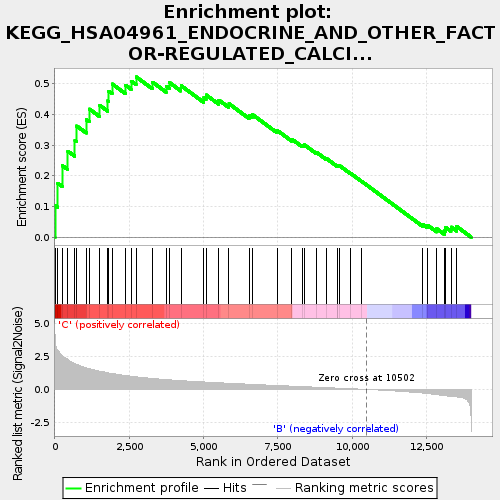

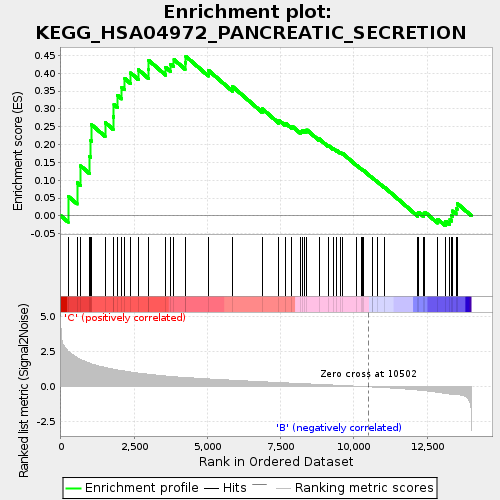

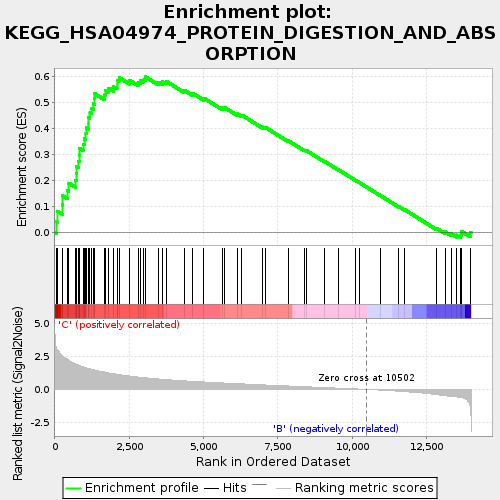

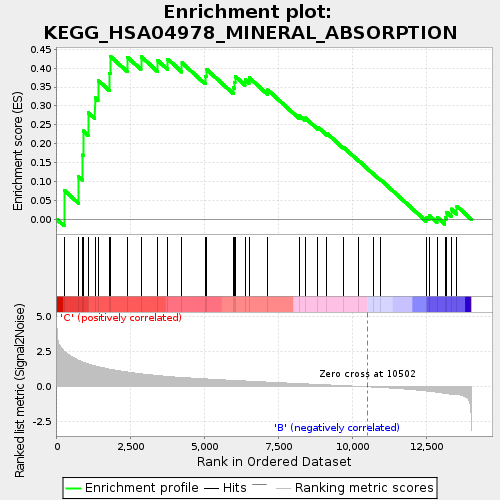

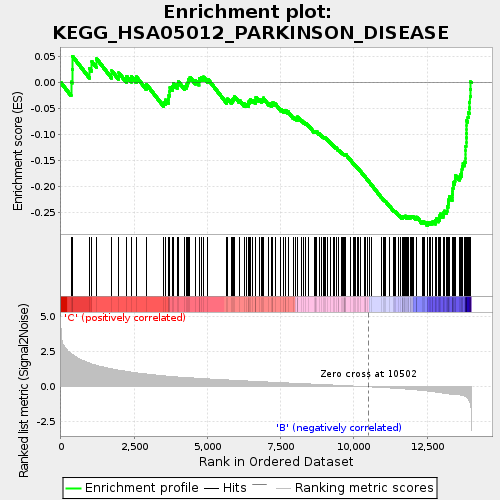

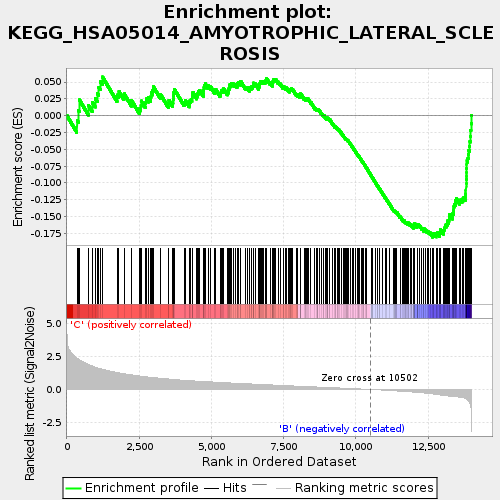

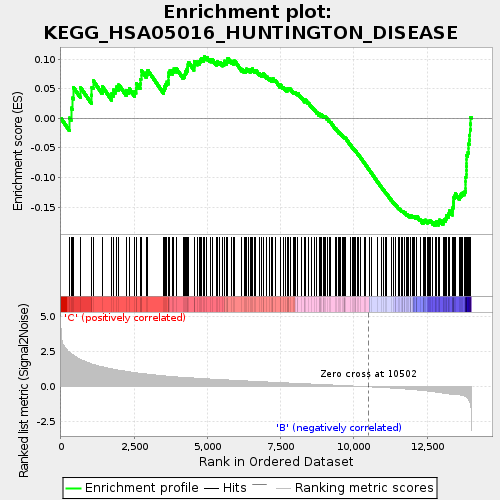

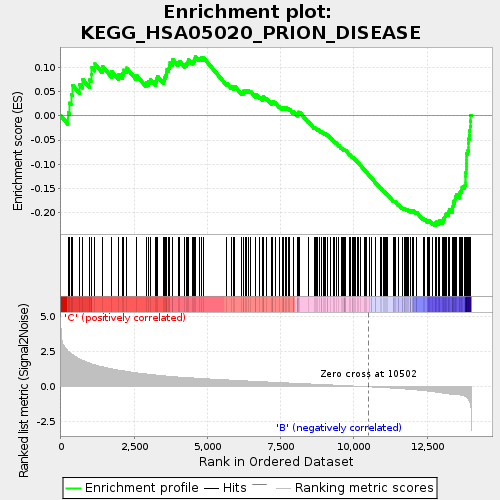

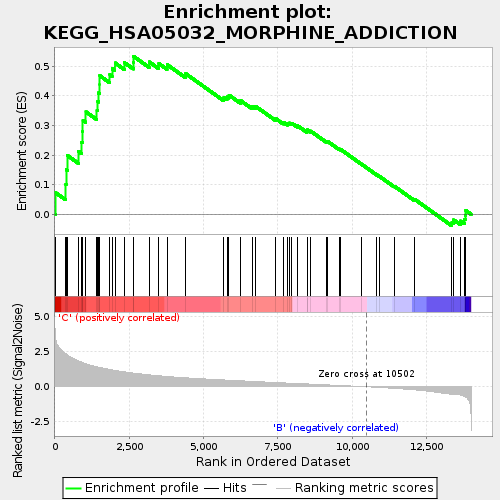

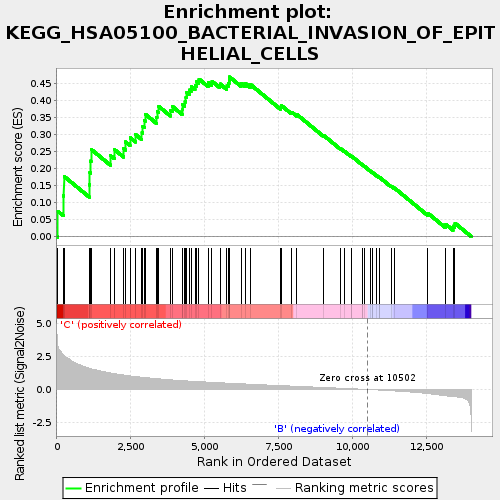

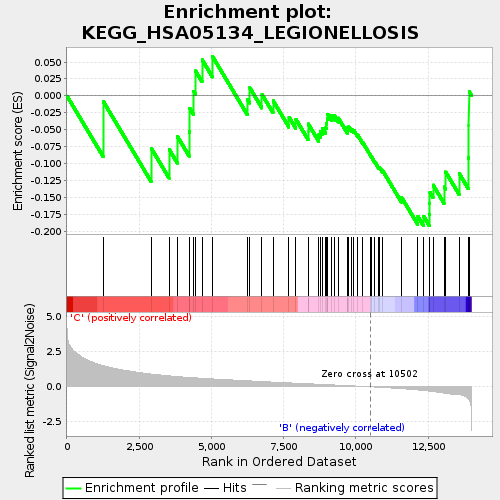

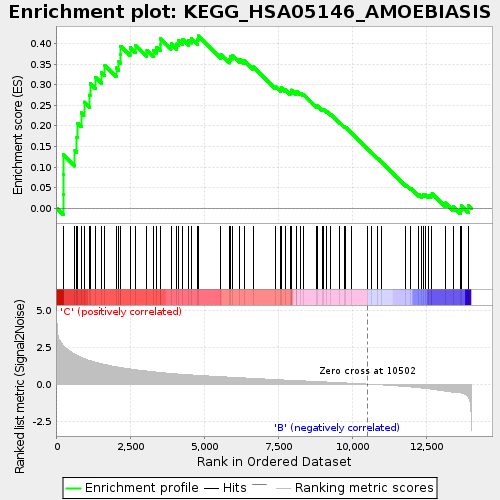

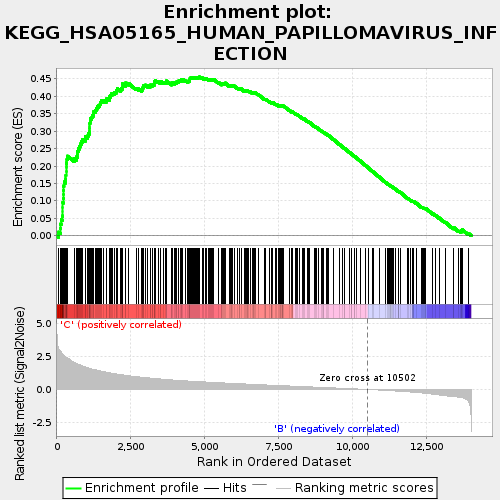

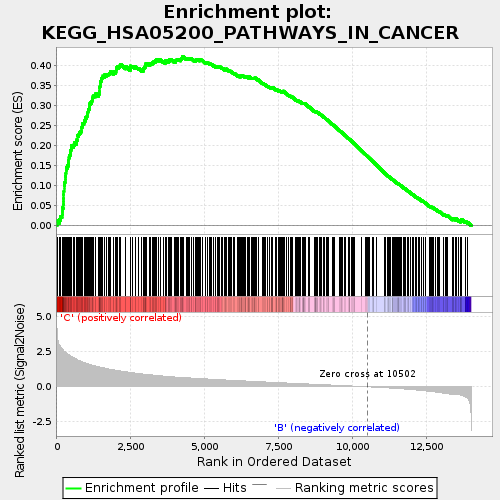

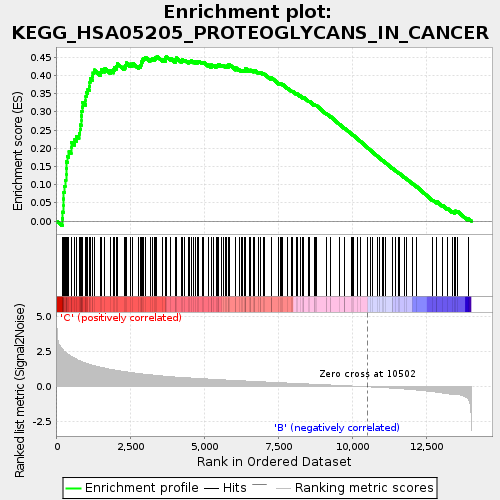

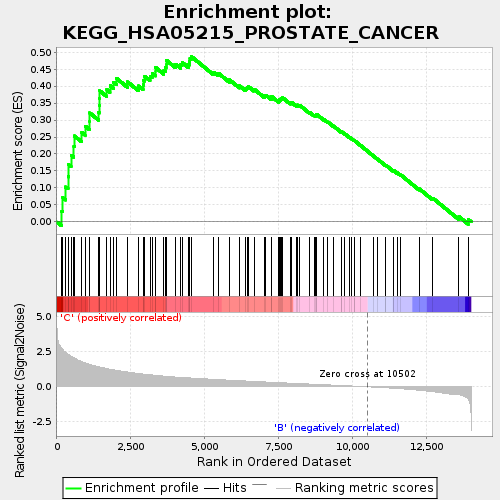

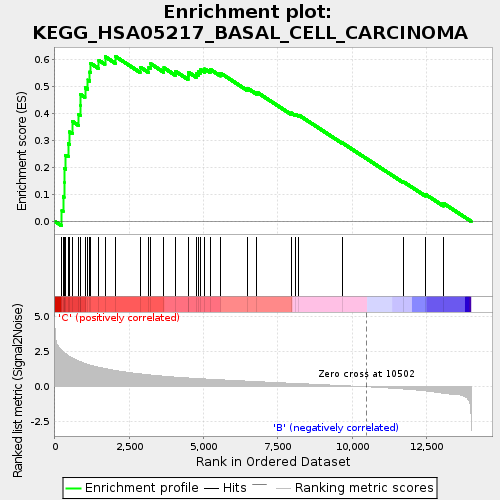

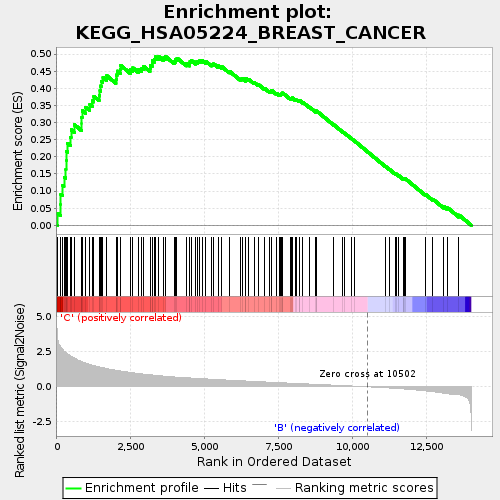

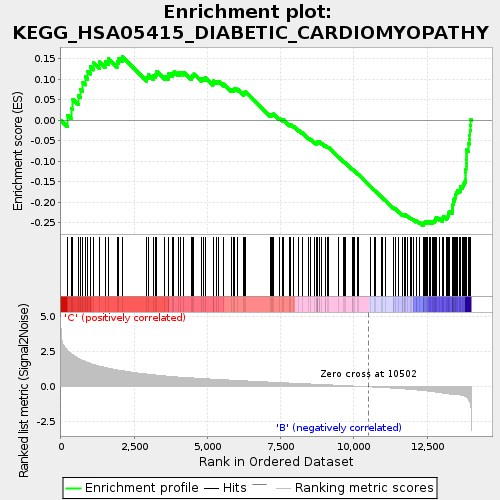

Supplement: Supplementary 6 — Gene set enrichment analysis of NI vs. CO. [file 1154808.f6.docx]
